# PLEKHG5 is stabilized by HDAC2-related deacetylation and confers sorafenib resistance in hepatocellular carcinoma

**DOI:** 10.1038/s41420-023-01469-z

**Published:** 2023-05-29

**Authors:** Yu Sha, Mingang Pan, Yunmeng Chen, Liangjun Qiao, Hengyu Zhou, Dina Liu, Wenlu Zhang, Kai Wang, Luyi Huang, Ni Tang, Jianguo Qiu, Ailong Huang, Jie Xia

**Affiliations:** 1 Key Laboratory of Molecular Biology on Infectious Diseases, Ministry of Education, Chongqing Medical University, Chongqing, 400016 China; 2 Henan University of Chinese Medicine, Zhengzhou, 450000 China; 3 College of Basic Medicine, Chongqing Medical University, Chongqing, 400016 China; 4 College of Nursing, Chongqing Medical University, Chongqing, 400016 China; 5 Department of Hepatobiliary Surgery, The First Affiliated Hospital of Chongqing Medical University, Chongqing, 400016 China

**Keywords:** Hepatocellular carcinoma, Cancer therapeutic resistance

## Abstract

Sorafenib is the first FDA-approved first-line targeted drug for advanced HCC. However, resistance to sorafenib is frequently observed in clinical practice, and the molecular mechanism remains largely unknown. Here, we found that PLEKHG5 (pleckstrin homology and RhoGEF domain containing G5), a RhoGEF, was highly upregulated in sorafenib-resistant cells. PLEKHG5 overexpression activated Rac1/AKT/NF-κB signaling and reduced sensitivity to sorafenib in HCC cells, while knockdown of PLEKHG5 increased sorafenib sensitivity. The increased PLEKHG5 was related to its acetylation level and protein stability. Histone deacetylase 2 (HDAC2) was found to directly interact with PLEKHG5 to deacetylate its lysine sites within the PH domain and consequently maintain its stability. Moreover, knockout of HDAC2 (HDAC2 KO) or selective HDAC2 inhibition reduced PLEKHG5 protein levels and thereby enhanced the sensitivity of HCC to sorafenib in vitro and in vivo, while overexpression of PLEKHG5 in HDAC2 KO cells reduced the sensitivity to sorafenib. Our work showed a novel mechanism: HDAC2-mediated PLEKHG5 posttranslational modification maintains sorafenib resistance. This is a proof-of-concept study on targeting HDAC2 and PLEKHG5 in sorafenib-treated HCC patients as a new pharmaceutical intervention for advanced HCC.

## Introduction

Hepatocellular carcinoma (HCC) is the sixth most commonly diagnosed cancer and the third leading cause of cancer-related mortality globally according to the World Health Organization’s Cancer Today statistics (https://gco.iarc.fr/today/home). Surgery and locoregional therapies are used in the treatment of early/intermediate-stage HCC, while systemic therapies are often used to treat advanced HCC [1]. The multitarget tyrosine kinase inhibitor sorafenib, which has antiangiogenic and antiproliferative effects, was the first FDA-approved systemic therapeutic drug for HCC [2]. However, the median survival advantage of sorafenib therapy in these patients is less than 3 months due to the high incidence of drug resistance [3]. Although new first-line systemic treatments such as lenvatinib and FOLFOX4 have been approved for HCC, clinical trials have confirmed that their effects on overall survival are seldom better than those of sorafenib, and sorafenib is still the standard of care for HCC systemic therapy [4, 5]. Thus, to improve the survival of HCC patients, new treatment options, such as combinational therapy targeting multiple signaling pathways, need to be explored to overcome drug resistance.

Sorafenib resistance can be acquired by activating alternative pathways through receptor tyrosine kinases (RTKs) to replace the loss of MAPK/Erk signaling in response to sorafenib-related Raf inhibition. Recent studies have demonstrated that p38-MAPK signaling [6], PI3K/AKT signaling [7] and NF-κB signaling [8] are activated in HCC cells and during the acquisition of sorafenib resistance. In addition, analyses of cancer stem cells and the tumor microenvironment illustrate that angiogenesis, inflammation, fibrosis, hypoxia, autophagy and viral reactivation are closely involved in the processes of sorafenib resistance [9–11]. However, none of these pathways fully account for the complex mechanism of sorafenib resistance, and patients with advanced recurrent HCC still have few choices for life-prolonging therapy.

The Rho family of GTPases is part of the Ras superfamily and plays important roles in different cellular processes [12–14]. Rho GTPases act as molecular switches, cycling between a GTP-bound active form and a GDP-bound inactive form. Their activity is increased by guanine nucleotide exchange factors (GEFs), which promote the release of bound GDP and subsequent binding of the more abundant GTP, and decreased by GTPase-activating proteins (GAPs), which stimulate the hydrolysis of GTP [14]. RhoGEFs and their related active forms, Rho GTPases, have been demonstrated to be required to enhance AKT phosphorylation and NF-κB and p38-MAPK activation [15–17]. Based on these crucial roles, Rho GTPases contribute to various pathological processes of cancer progression, including tumor initiation, growth and metastasis. For example, Rac1 is known to be closely related to cancer progression and drug resistance [18]. However, both Rho GTPases and Ras GTPases have been previously considered “undruggable” due to their globular structure with few small-molecule binding pockets, high affinity for GTP or GDP binding, and the micromolar level of GTP available in cells [19, 20]. Thus, targeting RhoGEFs by competitive inhibition of the molecular interactions between GEFs and their binding partners is thought to be the current choice for more potent Rho GTPase inhibitors in cancer therapy [21].

RhoGEFs are directly responsible for the activation of Rho GTPases in response to diverse extracellular or intracellular signaling pathways. Since then, approximately 80 RhoGEFs have been found, and they have been divided into two families according to their structure [22]. The majority of RhoGEFs are DBL-family GEFs, which contain a DBL-homology (DH) domain associated with a pleckstrin homology (PH) domain. The other is DOCK family GEFs, which are characterized by the presence of DHR1 and DHR2 domains. RhoGEFs are often regulated by posttranslational modifications, including phosphorylation, ubiquitylation and acetylation, to precisely regulate distinct stages of cellular processes [22]. Specifically, RhoGEFs have been found to frequently exhibit abnormal activation in different types of malignancies, including HCC, colorectal cancer, breast cancer, and pancreatic cancer, and to contribute to cancer cell proliferation, invasion, metastasis and even drug resistance [23, 24].

Here, we report that sorafenib resistance is substantially dependent on pleckstrin homology and RhoGEF domain containing G5 (PLEKHG5), a DBL-family RhoGEF related to Rho GTPase signaling. The underlying molecular mechanisms that mediate the sensitivity of HCC cells to sorafenib may be derived from HDAC2-related deacetylation of PLEKHG5. Moreover, an HDAC2 inhibitor sensitized HCC cells to sorafenib treatment by promoting acetylation-related degradation of PLEKHG5.

## Results

### PLEKHG5 is upregulated in sorafenib-resistant HCC cells

**Fig. 1:**
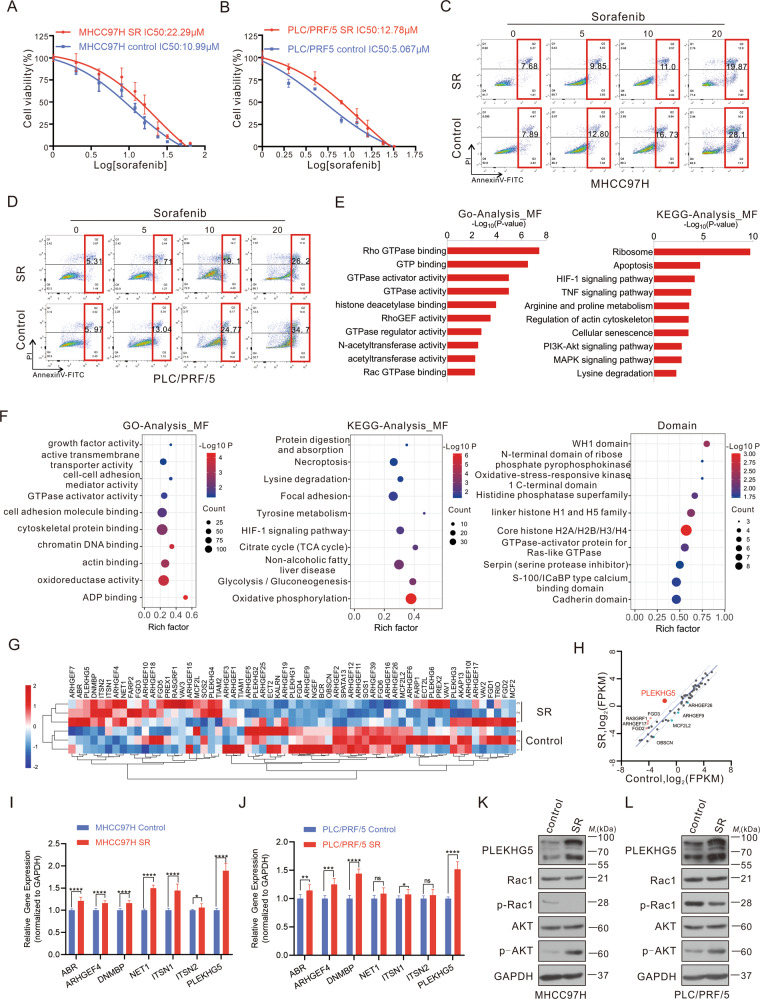
PLEKHG5 is upregulated in sorafenib-resistant HCC cells. **A** IC50 values of sorafenib for MHCC97H and MHCC97H-SR cells determined by CCK-8 assay. **B** IC50 values of sorafenib for PLC/PRF/5 and PLC5/PRF/5-SR cells determined by CCK-8 assay. **C** Apoptosis of MHCC97H and MHCC97H SR cells after 48 h treating with different doses of sorafenib detected by annexin V staining followed with flow cytometry assay. **D** Apoptosis of PLC/PRF/5 and PLC5/PRF/5 SR cells after 48 h treating with different doses of sorafenib detected by annexin V staining followed with flow cytometry assay. **E** Gene ontology and Kyoto Encyclopedia of Genes and Genomes analysis of different expressed genes between sorafenib sensitive and resistant cells. **F** Gene ontology, Kyoto Encyclopedia of Genes and Genomes and structural domain analysis of different expressed proteins between sorafenib sensitive and resistant cells. **G** Heat map of RhoGEFs obtained from RNA-sequencing data of MHCC97H SR and MHCC97H cells. **H** Correlation of RhoGEFs expression levels obtained from RNA-sequencing data of MHCC97H SR and MHCC97H cells. **I** qRT-PCR analysis of indicated mRNA levels in sorafenib sensitive and resistant MHCC97H cells. **J** qRT-PCR analysis of indicated mRNA levels in sorafenib sensitive and resistant PLC/PRF/5 cells. **K** Western blot analysis of indicated protein levels in sorafenib sensitive- and resistant- MHCC97H cells. **L** Western blot analysis of indicated protein levels in sorafenib sensitive and resistant PLC/PRF/5 cells. Error bars represent the mean ± SD from the biological triplicates, *P < 0.05, **P < 0.01, ***P < 0.001, ****P < 0.0001, one-way ANOVA analysis.

To explore the potential mechanisms of sorafenib resistance, MHCC97H and PLC/PRF/5 cells were used to establish sorafenib-resistant (SR) cell lines by continuous administration of low-dose sorafenib. After incubation for more than 6 months, the IC50 of sorafenib in both MHCC97H and PLC/PRF/5 cells increased more than 2-fold (Fig. 1A–B). The apoptosis levels of MHCC97H SR and PLC/PRF/5 SR cells at different doses of sorafenib were significantly decreased compared to their control counterparts (Fig. 1C–D; Fig. S1A). These results demonstrated the successful establishment of sorafenib-resistant HCC cells.

The differentially expressed gene (DEG) analysis was performed to identify the changes in transcript levels between sorafenib-resistant cells and their control counterparts. By RNA sequencing, a total of 2057 DEGs were detected (Fig. S1B-C). Interestingly, Gene Ontology (GO) analysis revealed that these DEGs were enriched in terms such as Rho GTPase activity, HDAC activity, and RhoGEF activity (Fig. 1E left panel). Moreover, KEGG analysis showed that sorafenib resistance was related to the PI3K/AKT and MAPK signaling pathways, which have been reported to be directly regulated by Rho GTPase activation (Fig. 1E, right panel) [6, 7, 15, 17].

To gain a comprehensive understanding of the mechanism of sorafenib resistance, tandem mass tag (TMT) quantitative proteomic analysis was used to identify differentially expressed proteins between sorafenib-resistant cells and their control counterparts. GO analysis revealed that these differentially expressed proteins were associated with GTPase activator activity and ADP binding (Fig. 1F, left panel). KEGG analysis revealed that sorafenib resistance was related to lysine degradation (Fig. 1F middle panel). Considering that protein‒protein interactions often occur in structural domains and that changes in amino acids or modifications within structural domains may cause changes in key protein functions, structural domain prediction is important for assessing the potential biological role played by differentially expressed proteins in sorafenib-resistant cells. The results showed that the structural domains of differentially expressed proteins were enriched in GTPase-activator protein for Ras-like GTPase domain and core histone H2A/H2B/H3/H4 domain (Fig. 1F right panel).

Considering the RNA-seq and TMT results, we hypothesized that sorafenib resistance is associated with the expression of Rho GTPases and Rho GTPase activators. Considering that RhoGEFs activate Rho GTPases and that RhoGEF inhibition is a more promising strategy than direct RhoGTPase inhibition, the expression levels of different RhoGEFs were validated. PLEKHG5 was identified as the most abundantly expressed among 61 RhoGEFs in sorafenib-resistant cells, as confirmed by qRT‒PCR and western blotting (Fig. 1G–L). Furthermore, we also observed dephosphorylation of Rac1, which indicated activation of Rac1 signaling [25] (schematically shown in Fig. S1D), and phosphorylation of AKT (p-AKT) and NF-κB/p65 (p-p65), accompanied by apparently elevated PLEKHG5 expression (Fig. 1K–L; Fig. S1E). In conclusion, sorafenib resistance may be related to PLEKHG5 upregulation and AKT/NF-kB signaling activation.

### PLEKHG5 is upregulated in HCC and correlated with the prognosis of HCC patients

**Fig. 2:**
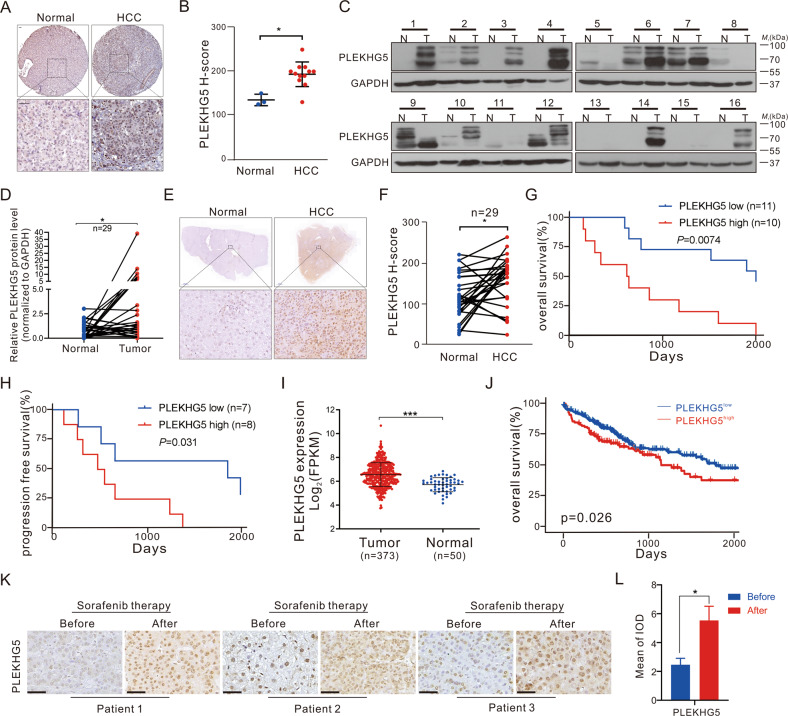
PLEKHG5 is upregulated in HCC and correlated with the prognosis of HCC patients. **A, B** Representative IHC images and H-score of PLEKHG5 protein expression in HCC tumor tissues and adjacent nontumor tissues from The Human Protein Atlas database. **C, D** Relative quantification of PLEKHG5 protein levels in HCC patients which were performed by western blot. Totally 32 pairs of HCC/ adjacent tissues were tested, and 29 pairs of PLEKHG5-detectable tissues were used for quantitative analysis. GAPDH served as the loading control. **E, F** IHC images and H-score of PLEKHG5 in HCC tissue of HCC patient cohort. **G, H** Kaplan-Meier estimation of PLEKHG5 OS and PFS based on the PLEKHG5 expression levels in the HCC patient cohort. **I** PLEKHG5 mRNA levels in HCC and paired non-tumor tissues from TCGA-LIHC dataset. **J** Overall survival curves of TCGA-LIHC analysis were performed by using ucscxenashiny tool in Hiplot (https://hiplot.com.cn), a comprehensive web platform for scientific data visualization. **K, L** Immunohistochemistry of PLEKHG5 in HCC tissue of patients from (**C**). The positive intensity was quantified by ImageJ Pro Plus. Error bars represent the mean ± SD, *P < 0.05, **P < 0.01, ***P < 0.001, ****P < 0.0001, one-way ANOVA analysis.

To explore the role of PLEKHG5 in HCC, we analyzed PLEKHG5 protein and mRNA expression levels in different databases. In The Human Protein Atlas database, IHC showed that PLEKHG5 protein levels are higher in liver cancer tissues than in corresponding nontumor tissues (Fig. 2A–B). Furthermore, we detected PLEKHG5 protein levels in 32 HCC patients by western blotting and found that approximately 65% of sample pairs showed significantly increased PLEKHG5 expression in tumor tissues compared with paired normal tissues, while PLEKHG5 was undetectable in most of the other samples (Fig. 2C–D; Fig. S2A). This result was further confirmed by IHC analyses with 29 paired HCC and adjacent nontumor tissues (Fig. 2E–F). We next examined the prognostic significance of PLEKHG5 expression in the HCC patient cohort by IHC. Importantly, high PLEKHG5 expression was associated with poor overall survival (OS) and progression-free survival (PFS) in the HCC patient cohort (Fig. 2G–H).

To further confirm the role of PLEKHG5 in HCC, we analyzed PLEKHG5 mRNA expression in The Cancer Genome Atlas (TCGA) database, and the results revealed that the PLEKHG5 mRNA level was upregulated in HCC patients, and increased with the progression of HCC (Fig. 2I; Fig. S2B). Kaplan‒Meier analysis revealed the overall survival benefits in HCC patients with low PLEKHG5 levels, and a similar result was found in HCC patient cohort (Fig. 2J). Collectively, these results indicate that PLEKHG5 is progressively upregulated during HCC development, and high expression of PLEKHG5 is correlated with an unfavorable prognosis in HCC patients.

Although the vast majority of HCC patients did not undergo surgery after recurrence, three of 32 HCC patients received sorafenib treatment and experienced recurrence. We compared PLEKHG5 protein levels before and after sorafenib treatment through immunohistochemistry and found that PLEKHG5 expression in these patients was significantly higher after relapse than before sorafenib treatment (Fig. 2K–L), which confirmed the involvement of PLEKHG5 in sorafenib resistance.

### PLEKHG5 expression correlates with HCC cell growth and promotes HCC cell sorafenib resistance

**Fig. 3:**
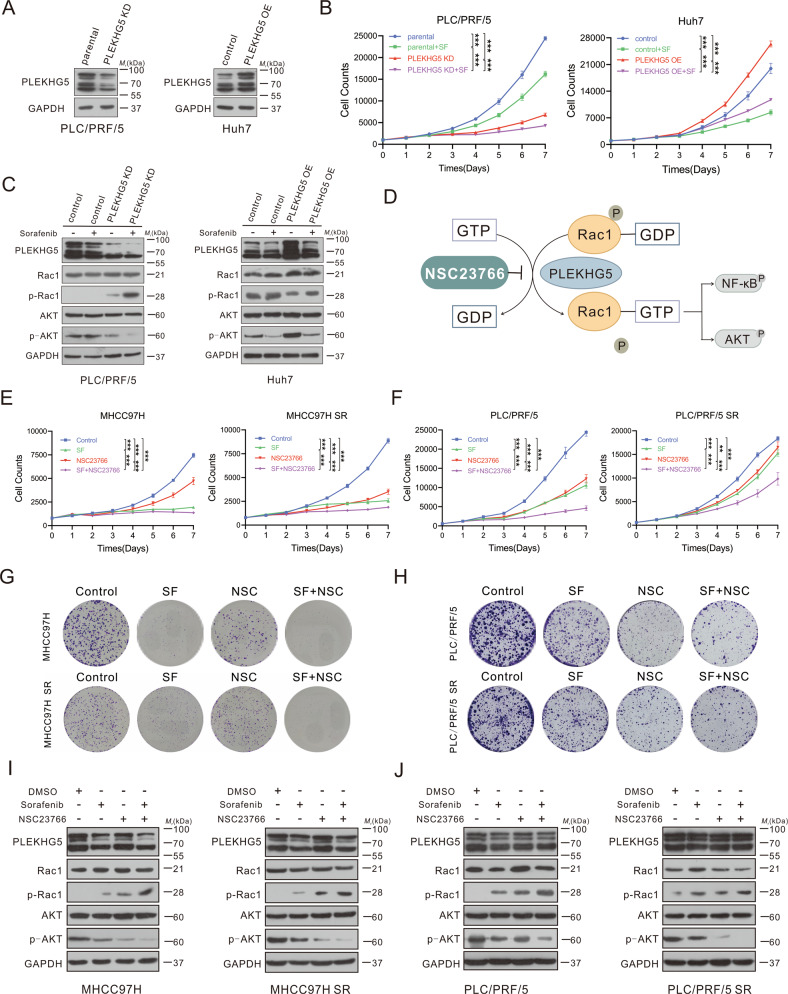
PLEKHG5 expression correlates with HCC cell growth and promotes HCC cell sorafenib resistance. **A** Detection of the efficiency of PLEKHG5 knockdown in PLC/PRF/5 cells and overexpression in Huh7 cells by Western blotting. GAPDH served as the loading control. **B** Continuous cell counts of PLEKHG5-KD, PLEKHG5-OE and their corresponding control cells treated with/without sorafenib by MTS assay. **C** Western blot analysis of the protein levels of PLEKHG5, Rac1, p-Rac1, AKT, and p-AKT in PLEKHG5-KD or PLEKHG5-OE cells treated with/without sorafenib for 48 h. GAPDH served as the loading control. **D** Schematic diagram of the mechanism of NSC23766 on the interaction between PLEKHG5 and Rac1 activity. **E**–**H** MTS assay and colony formation assays of sorafenib sensitive or resistant cells treated with sorafenib and/or NSC23766. **I**, **J** Western blot analysis of the protein levels of PLEKHG5, Rac1, p-Rac1, AKT, and p-AKT in sorafenib sensitive or resistant cells after treating with sorafenib and/or NSC23766 for 48 h. GAPDH served as the loading control. Error bars represent the mean ± SD, *P < 0.05, **P < 0.01, ***P < 0.001, ****P < 0.0001, one-way ANOVA analysis.

To investigate the biological effects of PLEKHG5 in sorafenib resistance, PRC/PLF/5 PLEKHG5 knockdown (PLEKHG5-KD) cells and Huh7 PLEKHG5-overexpressing (PLEKHG5-OE) cells were constructed (Fig. 3A). PLEKHG5 knockdown significantly inhibited cell proliferation and enhanced cell sensitivity to sorafenib, while PLEKHG5 overexpression inhibited the antiproliferative effect of sorafenib (Fig. 3B). We next detected the downstream targets and signaling pathways of PLEKHG5 by western blotting. The results showed that knockdown of PLEKHG5 increased the phosphorylation of Rac1 and dephosphorylation of downstream AKT and NF-κB/p65 compared with that in the control group, and treatment with sorafenib further enhanced these changes in phosphorylation. On the other hand, sorafenib reversed the trend of PLEKHG5 overexpression as well as the related changes in the phosphorylation levels of Rac1, AKT and NF-κB/p65 (Fig. 3C; Fig. S3A). In summary, PLEKHG5 promoted Rac1/AKT/NF-κB signaling to induce sorafenib resistance in HCC cells.

Next, we used the Rac1 inhibitor NSC23766 to disrupt the interaction between PLEKHG5 and Rac1 and confirm the role of PLEKHG5 in promoting sorafenib resistance (Fig. 3D). Interestingly, NSC23766 reversed the compensatory increase in AKT and NF-κB signaling in sorafenib resistance (Fig. S3B). In addition, NSC23766 in combination with sorafenib further inhibited cell proliferation and decreased p-AKT and p-p65 levels (Fig. 3E–J; Fig. S3C–E). Therefore, we confirmed that PLEKHG5 plays an important role in HCC progression and sorafenib resistance and may be a new effective therapeutic target in HCC therapies.

### HDAC2 interacts with PLEKHG5 and associates with its acetylation

**Fig. 4:**
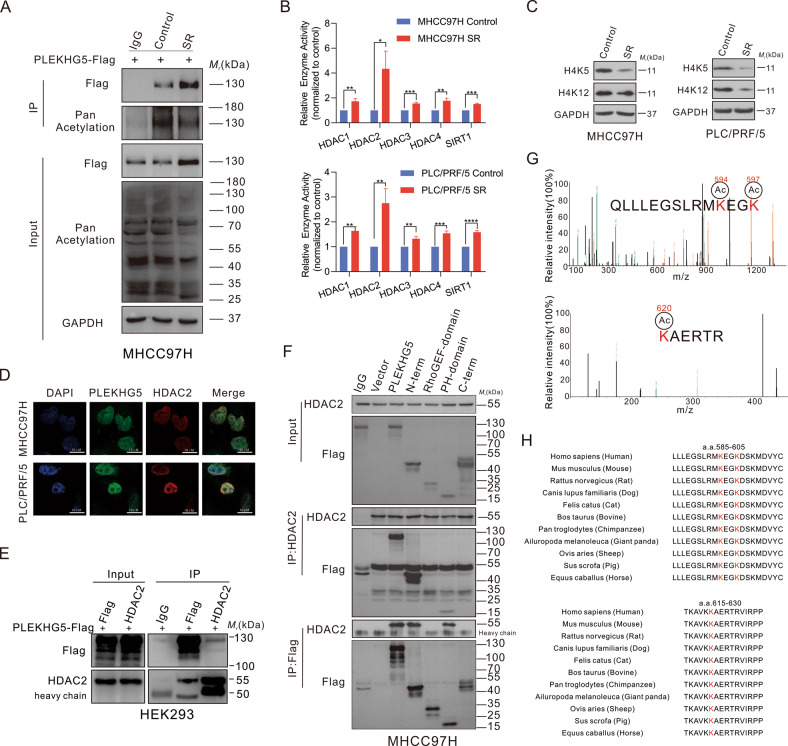
HDAC2 interacts with PLEKHG5 and associates with its acetylation. **A** IB analysis of WCL and anti-Flag IP derived from MHCC97H sorafenib sensitive and resistant cells transfected with PLEKHG5-Flag. **B** Enzyme activity of HDAC1, HDAC2, HDAC3, HDAC4 and SIRT1 detected by HDAC Activity Assay Kit. **C** Western blot analysis of the protein levels of H4K5 and H4K12 in MHCC97H, PLC/PRF/5 sorafenib sensitive and resistant cells. GAPDH served as the loading control. **D** Immunofluorescence (IF) verified the co-localization between HDAC2 (red) and PLEKHG5 (green) in MHCC97H and PLC/PRF/5 cells. **E** Co-IP analysis of HDAC2 and PLEKHG5-Flag in WCL of HEK293 cells transfected with PLEKHG5-Flag. **F** Co-IP assay of HDAC2 and Flag in WCL of MHCC97H cells transfected with indicated PLEKHG5 peptide Flag (PLEKHG5-Flag-WT, N-term-Flag, RhoGEF domain-Flag, PH domain-Flag or C-term-Flag). **G** Mass-spectrometry detection of Lys594, Lys597, and Lys620 acetylation followed by PLEKHG5 immunoprecipitation. **H** Multi-species conserved analysis of Lys594, Lys597, and Lys620 in pH domain. Error bars represent the mean ± SD from the biological triplicates, *P < 0.05, **P < 0.01, ***P < 0.001, ****P < 0.0001, one-way ANOVA analysis.

Given that RhoGEF activity may depend on its posttranslational modification [26] and that DEGs were involved in HDAC activity (Fig. 1E), which indirectly regulates protein posttranslational modifications through deacetylation of ε-amino lysine [27], we next evaluated the acetylation levels of PLEKHG5 in sorafenib-resistant cells. The expression of PLEKHG5 was increased, but the acetylation level was decreased in sorafenib-resistant cells (Fig. 4A; Fig. S4A). Since GO analysis revealed that DEGs were involved in HDAC activity (Fig. 1E), we further confirmed HDAC activity using enzyme activity kits. Surprisingly, HDAC2 enzyme activity was significantly increased, and lysine H4K5 and H4K12, targets of HDAC2, showed significant decreases in acetylation levels in sorafenib-resistant cells, suggesting that sorafenib resistance is related to increased HDAC2 enzyme activity (Fig. 4B–C). Thus, we hypothesized that the acetylation of PLEKHG5 might be related to HDAC2 activity. Surprisingly, HDAC2 and PLEKHG5 colocalized and bound with each other (Fig. 4D–E; Fig. S4B). This binding event seemed to involve the pleckstrin homology (PH) domain of PLEKHG5, which plays roles in interacting with other proteins (Fig. 4F; Fig. S4C–D) [28, 29]. We then performed mass spectrometric analysis and confirmed that there were three highly conserved lysine sites (K594, K597, and K620) in the PH domain that were modified by acetylation (Fig. 4G–H).

### HDAC2 deacetylates PLEKHG5 and maintains its protein stability

**Fig. 5:**
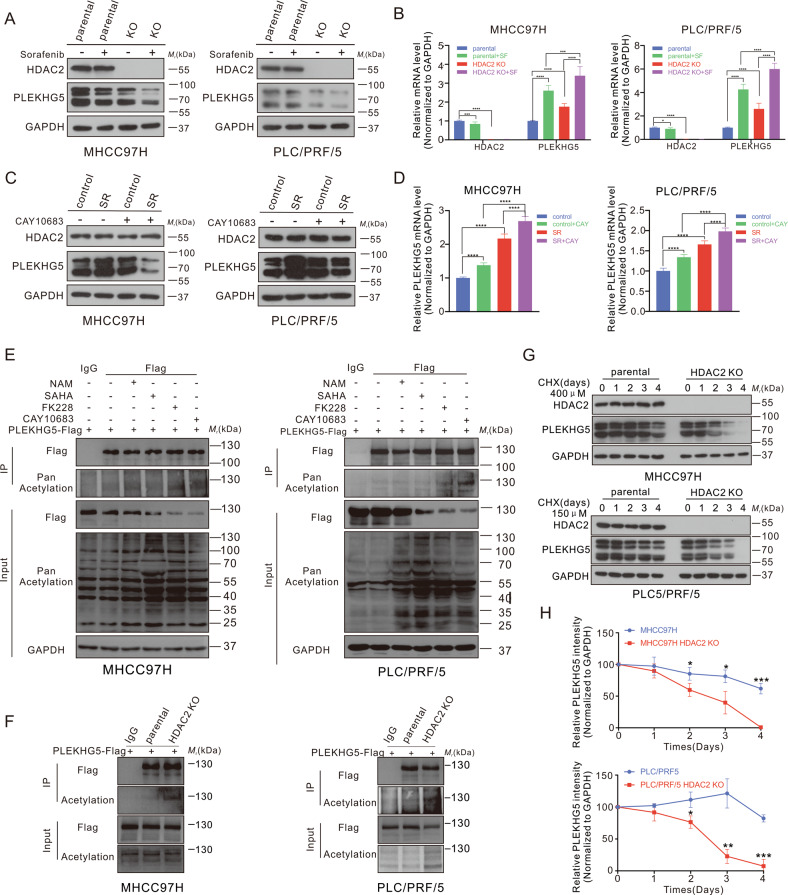
HDAC2 deacetylates PLEKHG5 and maintains its protein stability. **A** Western blot analysis of the protein levels of HDAC2 and PLEKHG5 in MHCC97H, PLC/PRF/5 parental and HDAC2 KO cells treated with or without sorafenib for 48 h. GAPDH served as the loading control. **B** qRT-PCR analysis of HDAC2 and PLEKHG5 levels in MHCC97H, PLC/PRF/5 parental and HDAC2 KO cells treated with or withoutsorafenib for 48 h. **C** Western blot analysis of the protein levels of HDAC2 and PLEKHG5 in MHCC97H, PLC/PRF/5 sorafenib sensitive and resistant cells treated with or without CAY10683 for 48 h. GAPDH served as the loading control. **D** qRT-PCR analysis of PLEKHG5 levels in MHCC97H, PLC/PRF/5 sorafenib sensitive and resistant cells treated with or without CAY10683 for 48 h. **E** IB analysis of WCL and anti-Flag IP derived from MHCC97H and PLC/PRF/5 cells transfected with PLEKHG5-Flag in the presence or absence of the NAM, SAHA, FK228, or CAY10683. **F** IB analysis of WCL and anti-Flag IP derived from parental and HDAC2 KO cells transfected with PLEKHG5-Flag. **G**, **H** Western blot analysis of the protein levels of HDAC2 and PLEKHG5 in parental and HDAC2 KO cells treated with CHX. Relative protein intensity of PLEKHG5 was quantified by ImageJ. GAPDH served as the loading control. Error bars represent the mean ± SD from the biological triplicates, *P < 0.05, **P < 0.01, ***P < 0.001, ****P < 0.0001, one-way ANOVA analysis.

To further investigate the relationship between HDAC2 and PLEKHG5 in HCC, HDAC2 knockout (HDAC2 KO) cells were constructed (Fig. S5A–B). To our surprise, the level of PLEKHG5 protein, but not mRNA, was significantly reduced in HDAC2 KO cells (Fig. 5A–B; Fig. S5C). Santacruzamate A (CAY10683) treatment confirmed that PLEKHG5 protein levels were affected by HDAC2 inhibition (Fig. 5C–D). However, the HDAC2 protein level was not affected by PLEKHG5 (Fig. S5D). These results suggest that PLEKHG5 is downstream of HDAC2, but HDAC2 does not regulate PLEKHG5 transcription. Thus, we presumed that HDAC2 affects PLEKHG5 protein levels by regulating its acetylation. To assess whether HDAC2 is the potential physiological deacetylase for PLEKHG5, we treated cells with the pan-SIRT inhibitor nicotinamide (NAM), pan-HDAC inhibitor vorinostat (SAHA), selective HDAC2 inhibitor CAY10683, and HDAC1/2 inhibitor romidepsin (FK228). We found that CAY10683 and FK228, which could target HDAC2, increased the acetylation of PLEKHG5 (Fig. 5E). Consistent with this finding, knockout of HDAC2 increased PLEKHG5 acetylation, indicating that HDAC2 is the bona fide deacetylase of PLEKHG5 (Fig. 5F). Thus, we presumed that HDAC2 regulates PLEKHG5 protein levels by posttranslational regulation (for example, by affecting its protein stability). Interestingly, when cycloheximide (CHX) was used to inhibit protein synthesis, PLEKHG5 protein stability was significantly reduced due to the knockout of HDAC2 in MHCC97H and PLC/PRF/5 cells (Fig. 5G–H). To evaluate the correlation between PLEKHG5 stability and its acetylation at lysine sites, we mutated the three lysine sites (K) to glutamine (Q) in both MHCC97H and PRF/PLC/5 cells to mimic acetylation and found that the protein stability of PLEKHG5 was significantly reduced (Fig. S5E–F). Concordantly, the stability of PLEKHG5 was significantly reduced when all three lysine sites were mutated to arginine (R) to mimic deacetylation in HDAC2 KO cells (Fig. S5G–H). These results suggest that HDAC2 interacts with PLEKHG5 in the PH domain and deacetylates K594, K597, and K620 to maintain the stability of PLEKHG5.

### Knockout of HDAC2 enhances the sensitivity of HCC to sorafenib in vitro and in vivo

**Fig. 6:**
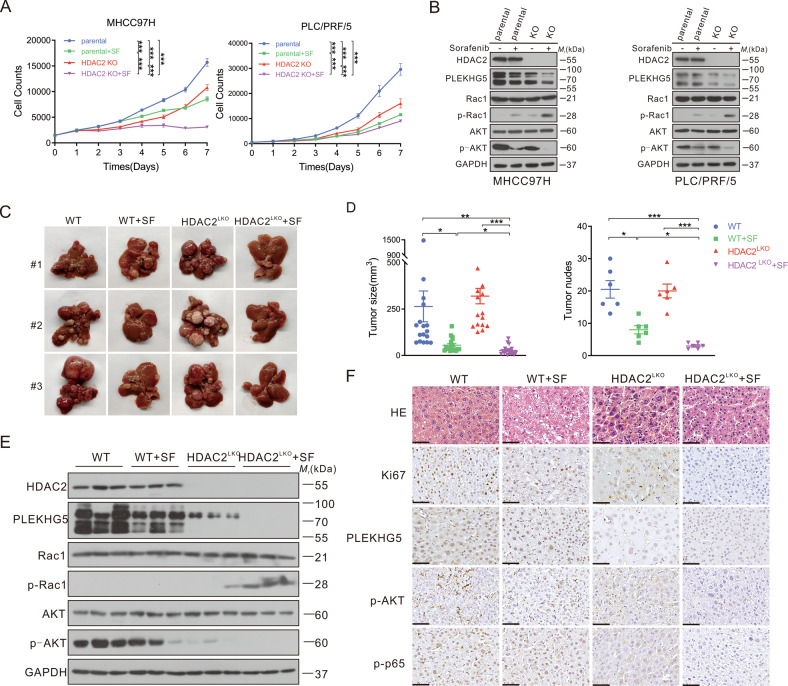
Knockout of HDAC2 enhances the sensitivity of HCC to sorafenib in vitro and in vivo. **A** Continuous cell counts of parental and HDAC2 KO cells treated with/without sorafenib by MTS assay. **B** Western blot analysis of the protein levels of HDAC2, PLEKHG5, Rac1, p-Rac1, AKT, and p-AKT in parental and HDAC2 KO cells treated with or without sorafenib for 48 h. GAPDH served as the loading control. **C** Gross appearances of murine livers of WT and HDAC2^LKO^ mice treated with DEN intraperitoneal injection and followed with/without sorafenib therapy. **D** Measurement of HCC tumor size and number in each group from (**C**). **E** Western blot analysis of the protein levels of HDAC2, PLEKHG5, Rac1, p-Rac1, AKT, and p-AKT in each group from (**C**). GAPDH served as the loading control. **F** H&E staining and immunohistochemistry of Ki67, PLEKHG5, p-AKT, and p-p65 of each group from (**C**). Bar represents 200 μM. Error bars represent the mean ± SD, *P < 0.05, **P < 0.01, ***P < 0.001, ****P < 0.0001, one-way ANOVA analysis.

Previous research proved that high HDAC2 expression confers drug resistance toward the topoisomerase II inhibitor etoposide in pancreatic ductal adenocarcinoma cells [30]. Thus, we hypothesized that the high activity of HDAC2 in HCC might promote sorafenib resistance by maintaining PLEKHG5 stability. Indeed, the knockout of HDAC2 significantly repressed cell growth and enhanced sensitivity to sorafenib in HCC cells (Fig. 6A; Fig. S6A). PLEKHG5 protein was significantly decreased after HDAC2 knockout, while p-Rac1 expression was increased. As a result, p-AKT and p-p65 levels decreased (Fig. 6B; Fig. S6B–C). Moreover, growth inhibition and apoptosis induction driven by HDAC2 knockout were reversed by PLEKHG5 overexpression in both MHCC97H and PLC/PRF/5 cells (Fig. S6D–F), further confirming that increasing HDAC2 activity promotes sorafenib resistance through upregulation of PLEKHG5.

To gain a better understanding of HDAC2 in sorafenib resistance, liver-specific HDAC2 knockout C57BL/6 mice (HDAC2^LKO^) were used for in vivo validation. The mouse model of HCC in situ was successfully established by intraperitoneal injection of DEN and CCl4. Interestingly, HDAC2 deficiency in hepatocytes did not affect tumorigenesis. Sorafenib treatment led to further inhibition of tumor growth in HDAC2^LKO^ mice compared with wild-type mice (Fig. 6C–D). The immunohistochemistry (IHC) and western blotting results revealed that acute sorafenib treatment slightly affected the levels of PLEKHG5, p-Rac1, p-AKT, and p-p65, while the loss of HDAC2 resulted in significant changes (Fig. 6E–F; Fig. S6G). Additionally, the Ki67 index further confirmed that the knockout of HDAC2 could enhance the role of sorafenib in HCC therapy (Fig. 6F).

### Selective HDAC2 inhibition attenuates sorafenib resistance in HCC

**Fig. 7:**
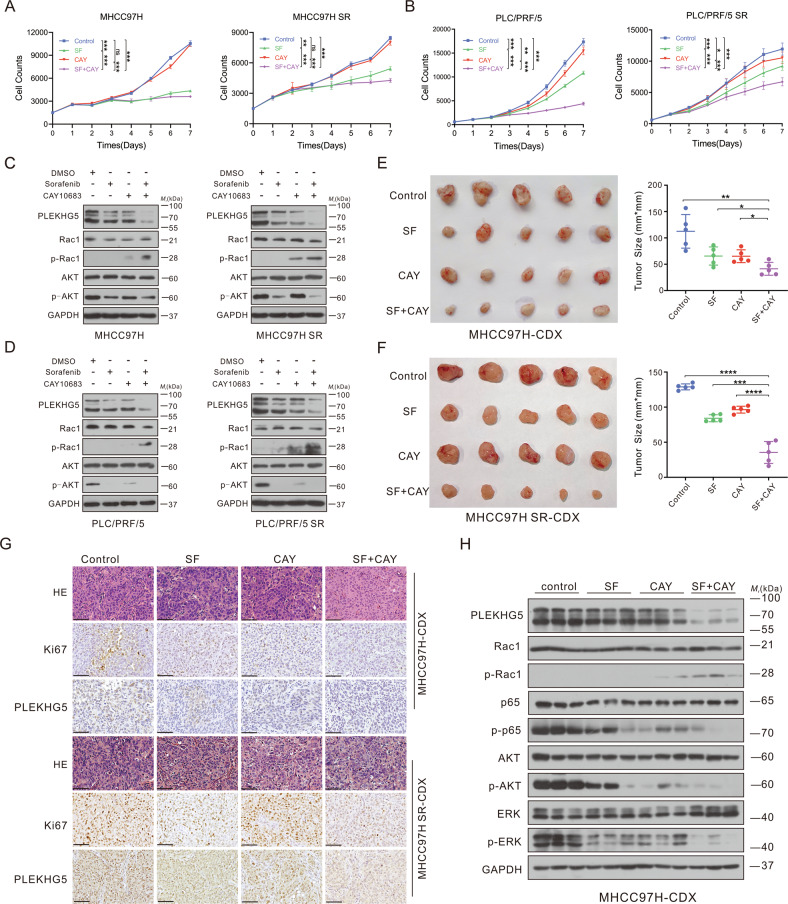
Selective HDAC2 inhibition attenuates sorafenib resistance in HCC. HCC cells were divided into 4 groups, one group served as control, and the others were treated with 1‰ DMSO, sorafenib, CAY10683 and CAY10683 combined with sorafenib, respectively. **A** Continuous cell counts of MHCC97H and MHCC97H SR cells treated with sorafenib and/or CAY10683 by MTS assay. **B** Continuous cell counts of PLC/PRF/5 and PLC/PRF/5 SR cells treated with sorafenib and/or CAY10683 by MTS assay. **C** Western blot analysis of the protein levels of PLEKHG5, Rac1, p-Rac1, AKT and p-AKT in MHCC97H and MHCC97H SR cells treated with sorafenib and/or CAY10683. **D** Western blot analysis of the protein levels of PLEKHG5, Rac1, p-Rac1, AKT and p-AKT in PLC/PRF/5 and PLC/PRF/5 SR cells treated with sorafenib and/or CAY10683. **E** Subcutaneous xenograft tumor formation with MHCC97H cells followed by treatment with intraperitoneal injection of drug solvent or sorafenib or CAY10683. Measurement of xenograft tumor size in each group. **F** Subcutaneous xenograft tumor formation with MHCC97H SR cells followed by treatment with intraperitoneal injection of drug solvent or sorafenib or CAY10683. Measurement of xenograft tumor size in each group. **G** H&E staining and immunohistochemistry of Ki67 and PLEKHG5 of each group from MHCC97H and MHCC97H SR cells-derived xenograft. Bar represents 200 μM. **H** Western blot analysis of the protein levels of PLEKHG5, Rac1, p-Rac1, p65, p-p65, AKT, p-AKT, ERK and p-ERK in each group from MHCC97H-CDX. Error bars represent the mean ± SD, *P < 0.05, **P < 0.01, ***P < 0.001, ****P < 0.0001, one-way ANOVA analysis.

There are no known inhibitors of PLEKHG5, whereas several HDAC inhibitors (HDACis) have been approved for clinical trials, even as second-line clinical agents [31]. Considering this and our findings that HDAC2 maintains PLEKHG5 stability, we hypothesized that selective HDAC2 inhibition might overcome sorafenib resistance in HCC. Thus, we evaluated the therapeutic effect of CAY10683 to determine whether it can be used in combination with sorafenib to treat sorafenib-resistant HCC cells. CAY10683 treatment alone barely inhibited cell proliferation and induced apoptosis. However, combination treatment with CAY10683 and sorafenib significantly inhibited cell growth and increased apoptosis (Fig. 7A–B; Fig. S7A–C). Consistent with the results in HDAC2 KO cells, obvious inhibition of PLEKHG5 and activation of Rac1/AKT/NF-κB signaling were observed after combined treatment (Fig. 7C–D; Fig. S7D–E).

Then, the therapeutic effect of CAY10683 combined with sorafenib was evaluated in two subcutaneous xenograft tumor models in nude mice. Notably, the combination of CAY10683 and sorafenib greatly slowed tumor growth compared to that in the sorafenib-treated group in both the sorafenib-sensitive cell-derived xenograft (CDX) model and the sorafenib-resistant CDX model (Fig. 7E–F). The tumor cell proliferation marker Ki67 was detected by IHC, and CAY10683 treatment alone did not reduce the proliferating HCC cell ratio in vivo, while administration of CAY10683 and sorafenib distinctly inhibited HCC cell proliferation in sorafenib-sensitive and -resistant CDX models (Fig. 7G). Additionally, the IHC and western blotting results showed that CAY10683 usage resulted in a significant decrease in PLEKHG5, p-AKT, and p-p65 levels and an increase in p-Rac1 levels (Fig. 7G–H Fig. S7F). Thus, our studies provide a molecular mechanism and rationale for combining HDAC2 inhibition with sorafenib treatment as an effective combinational strategy for advanced HCC that is resistant to sorafenib.

## Discussion

Due to the lack of effective therapies for HCC, the search for more reliable biological targets, especially those that can be used for the treatment of sorafenib-resistant HCC, is an important and urgent task. In the present work, we found that Rho GTPases, which have been proven to be upstream of AKT, NF-κB, and p38/MAPK signaling [23], and their activator RhoGEFs were upregulated in our sorafenib-resistant cell models. Our additional analysis and other recent publications on RNA-seq of SR cells [32] showed that PLEKHG5 is a consistently upregulated RhoGEF, indicating that it might be the initial inducer of sorafenib resistance. PLEKHG5 was commonly upregulated in human HCC, revealing that excess PLEKHG5 was related to poor survival and was an unfavorable prognostic indicator for HCC patients.

Based on its RhoGEF domain, PLEKHG5 is predicted to facilitate the activation of CDC42, Rac1 and RhoA [33]. Among these, the activated form of Rac1 has been reported to play a role in initiating AKT/mTOR [34], NF-κB [35], and MAPK signaling [36]. Consistent with our hypothesis, inhibition of Rac1 activity by knockdown or NSC23766 treatment was associated with increased Rac1 phosphorylation and reversed AKT/NF-κB signaling in both sorafenib-resistant and sorafenib-sensitive HCC cells, consequently enhancing the antiproliferative effect of sorafenib. Here, we identified a novel mechanism of sorafenib resistance driven by PLEKHG5-related Rac1 activation and provided a promising therapeutic target for advanced HCC.

RhoGEFs are reported to be phosphorylated, ubiquitinated, and acetylated, allowing productive interactions with substrate GTPases [26, 33, 37]. However, the regulatory mechanism for PLEKHG5 posttranslational modification remains incompletely understood. In our research, IP-MS was used to explore posttranslational modifications of PLEKHG5, and three conserved lysine sites in the PH domain were detected to be both acetylated and ubiquitinated. Interestingly, Loredana D’Amato [37] and Eun Hyeon Song [26] et al. found that the expression and activity of RhoGEFs are closely related to their acetylation level, which is consistent with our findings that PLEKHG5 acetylation was decreased in sorafenib-resistant cells, indicating that the activity of PLEKHG5 may be related to its acetylation. It is known that acetylation always positively or negatively correlates with ubiquitination, which is highly related to protein stability. Therefore, we focused on the acetylation modification of PLEKHG5. The PH domain mainly functions in interacting with other proteins, and previous research has shown that RhoGEF expression is regulated by HDACis [37], indicating that HDACs might respond to PH domain acetylation and RhoGEF expression. Our results that the selective HDAC2 inhibitors CAY10683 and FK228 unexpectedly increased the acetylation of PLEKHG5, that HDCA2 directly binds with PLEKHG5, and that knockout of HDAC2 correlated with a high acetylation level and instability of PLEKHG5 support the notion that HDAC2 is a bona fide deacetylase for PLEKHG5. Furthermore, although HDAC2 was detected to bind not only to the PH domain but also to the N-terminal domain, we mainly analyzed the interaction between HDAC2 and the PH domain because our IP-MS results did not show acetylation in the N-terminal domain. Based on the results from acetylation-mimetic lysine to glutamine and deacetylation-mimetic lysine to arginine mutants, HDAC2 was confirmed to regulate lysine deacetylation of the PH domain to affect PLEKHG5 stability.

Over the past decade, a large amount of evidence has illustrated the critical roles of HDACs in cell proliferation and cancer progression. However, HDAC inhibitor monotherapy showed less therapeutic effect combination therapies with HDACis in both HCC and melanoma, which might be attributed to the idea that certain signaling pathways required to activate cell death pathways are only activated by HDACis in drug-resistant cells but not in parental cells [38, 39]. Indeed, inhibition of HDAC2 alone was ineffective for HCC therapy in vitro and in vivo according to our previous and present results [40]. Consistent with Robbie Carson et al., who verified that HDAC inhibitors combined with MEK inhibitors could effectively overcome resistance to MEK inhibition in BRAF-mutant colorectal cancer [41], we found that knockout and pharmacological inhibition of HDAC2 combined with sorafenib could reverse sorafenib resistance. Therefore, we defined HDAC2 as a new potential target for the treatment of liver cancer that acquired sorafenib resistance.

**Fig. 8:**
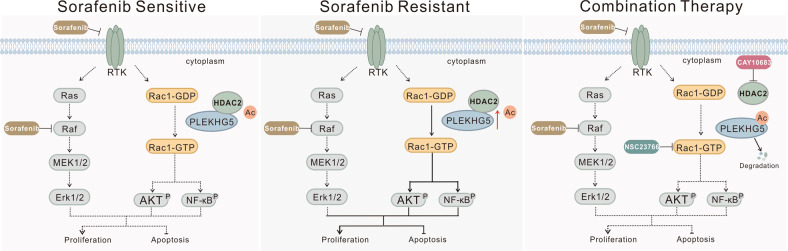
Model for the combination treatment of HCC resistant to sorafenib. HCC cells with normal Raf-MAPK signaling are sensitive to sorafenib. Sorafenib resistance develops through the increasing HDAC2 activity and upregulation of master regulator PLEKHG5, and leads to enhanced signaling through Rac1 activation related AKT pathway, NF-KB pathway, or MAPK pathway. Switching therapy from sorafenib to CAY10683 combined with sorafenib in sorafenib-resistant cells induces PLEKHG5 acetylation and degradation. The decreased PLEKHG5 then acts on Rac1 inactivation and followed with suppression of AKT pathway, NF-KB pathway, or MAPK pathway, and finally leads to a massive apoptosis that has a lethal effect on the cells.

In conclusion, we found that PLEKHG5 expression was increased in human HCC and was further increased in sorafenib-resistant HCC (Fig. 8) PLEKHG5-related drug-resistance effects were mediated through regulation of the Rac1/AKT/NF-κB signaling cascades. HDAC2 plays roles in deacetylation of the PH domain of PLEKHG5 and maintains its protein stability. Inhibiting HDAC2 could induce PLEKHG5 acetylation-related degradation, thus reversing sorafenib resistance in HCC.

## Materials and methods

### Reagents and antibodies

All reagents and antibodies used in this research are listed in Supplementary table 1.

### Cell lines

The human HCC cell lines (PLC/PRF/5, Huh7 and MHCC97H) were used in the study. PLC/PRF/5 and Huh7 cells were purchased from the US. ATCC with cell authentication. MHCC97H cell was obtained from the Liver Cancer Institute, Zhongshan Hospital, Fudan University, Shanghai, China. Cells were cultured in Dulbecco’s modified Eagle’s medium (HyClone, UT) supplemented with 10% fetal bovine serum (Natocor, Cordoba, Argentina), 100 IU penicillin, and 100 mg/ml streptomycin (HyClone, UT). All cells were incubated in a humidified atmosphere at 37 °C containing 5% CO2.

Sorafenib-resistant clones were established by continuous treatment of cells with sorafenib. The dose of sorafenib is initialed by 2 μM and gradually increased every week for more than six months until maximum tolerated dosage was reached. DMSO was used as a placebo controlled.

### Patient samples

The Institute Research Medical Ethics Committee of First Affiliated Hospital of Chongqing Medical University (Chongqing, China) granted approval for this study. At the time of tissue collection, informed consent was obtained from all patients. Fresh and formalin-fixed tissue samples from patients with HCC were collected randomly from the Department of Hepatobiliary Surgery, the First Affiliated Hospital of Chongqing Medical University. These samples were frozen and stored in liquid nitrogen immediately after surgery or fixed with formalin and embedded in paraffin for subsequent experiments. In addition, The Cancer Genome Atlas (TCGA) data of LICH (Liver Hepatocellular Carcinoma) were analyzed with GEPIA (http://gepia.cancer-pku.cn/) or UALCAN (http://ualcan.path.uab.edu/index.html) website.

### Animal model

Nude mice purchased from HUNAN SJA LABORATORY ANIMAL CO., LTD, were used for subcutaneous xenograft models. Liver-specific C57BL/6 HDAC2 knockout (HDAC2^LKO^) mice were kindly supplied by professor Yujun Shi (Sichuan University, Chengdu, China). All mice were housed in standard conditions with a 12-hour light/dark cycle and had access to food and water ad libitum. All mice were randomly grouped, and each group included at least 5-6 mice to ensure the reproducibility of the experiment The studies involving animal experimentation were approved by the Chongqing Medical University Animal Care and Use Committee and followed the National Institutes of Health guidelines on the care and use of animals.

Xenografts were established in 4- to 6-week-old nude mice using MHCC97H or MHCC97H SR cells. Sorafenib (20 mg/kg) and HDAC2 inhibitor CAY10683 (10 mg/kg) intraperitoneal injection were performed twice a week for 4 weeks when the size of the xenograft reached approximately 5 × 5 mm (length × width). Sorafenib and CAY10683 were used at the dosages suggested for in vivo experiments by the official Selleck website (https://www.selleck.cn/).

2-week-old C57BL/6 HDAC2^LKO^ and WT C57BL/6 (12 males and 12 females) mice were used to induce primary HCC by intraperitoneal administration of diethylnitrosamine (DEN, 100 mg/kg) and carbon tetrachloride (CCl4, 1 ml/kg, twice a week for 16 weeks). Since the 17th week, 20 mg/kg of sorafenib or matching placebo treatment was performed by intraperitoneal injection twice a week for 4 weeks. Mice were sacrificed at the end of 20th week, and their livers were collected for western blotting and immunohistochemical staining.

### Statistical analysis

Statistical analysis and data plotting were performed using GraphPad Prism 8. Data are presented as the Means ± SD. The one-way ANOWA followed by Tukey’s multiple comparison tests were used to compare two groups under multiple conditions, and the Gehan-Breslow-Wilcoxon test was used for survival analysis. Statistical significance was defined as *P* < 0.05.

Additional information can be found in the supplementary materials.

## Supplementary information

Supplementary material

Supplementary Figure S1

Supplementary Figure S2

Supplementary Figure S3

Supplementary Figure S4

Supplementary Figure S5

Supplementary Figure S6

Supplementary Figure S7

Western Blot

## Data Availability

The data of this study are available in the article and/or supplementary materials. The data in Fig. 1 were based on publicly available data from the Gene Expression Omnibus (GEO; https://www.ncbi.nlm.nih.gov/geo/query/acc.cgi?acc=GSE176151). Readers are welcome to contact the corresponding author for the raw data used in this work.
