# Supplementary material for: PLEKHG5 is stabilized by HDAC2-related deacetylation and confers sorafenib resistance in hepatocellular carcinoma
Source: Cell Death Discov. 2023 May 29;9:176. doi: 10.1038/s41420-023-01469-z (PMC10227013; doi:10.1038/s41420-023-01469-z)
Supplement: Supplementary file 1 — Supplementary material [file 41420_2023_1469_MOESM1_ESM.docx]

Supplementary Materials for

PLEKHG5 is stabilized by HDAC2-related deacetylation and confers sorafenib resistance in hepatocellular carcinoma

**This file includes:**
Supplementary Materials and Methods

Supplementary Figure Legends

Supplementary Table 1 to 2

**Materials and Methods**

Cell proliferation assays

Cell counting and colony formation assays were used to measure cell proliferation rates. Cells were seeded into 96-well plates at 1×10^3 cells per well and were scanned every 24 h. The quantified time-lapse curves were generated by IncuCyte ZOOM® Live-Cell Imaging system (Essen BioScience, MI). For the colony formation assay, 6 ×10^2 cells per well were seeded into six-well plates and cultured for 2 weeks. Cell colonies were then stained with crystal violet and photographed. Each experiment was performed at least in duplicate and the experiment was repeated at least three times.

Cell apoptosis

Cells were seeded into 6-well plates at a density of 5 ×10^4 cell/well. Cell apoptosis was analyzed by flow cytometry after treatment with sorafenib and/or CAY10683. Annexin-V-FITC Apoptosis Detection Kit (BD Biosciences, CA) was used in apoptosis detection according to the manufacturer’s instructions. The experiment was repeated at least three times.

qRT-PCR

Total RNA extracted from cells with different treatment was reverse-transcribed using a GoScript^TM^ Reverse transcriptase (Promega, Madison, WI, USA) according to the manufacturer’s instructions. Quantification of target genes was performed by SYBR Green qPCR (Bimake, USA) on a CFX Real-Time PCR Detection System (Bio-Rad, CA). All primers used in this study are listed in supplemental table 2. Each experiment was performed in triplicate and the experiment was repeated at least three times.

Tandem Mass Tags (TMT) quantitative proteomic analysis

MHCC97H and MHCC97H SR cells were dissociated in SDT buffer(4%SDS，100mM Tris-HCl，1mM DTT，pH7.6). Protein digestion by trypsin was performed according to filter-aided sample preparation (FASP) procedure described by Matthias Mann. 100 μg peptide mixture of each sample was labeled using TMT reagent according to the manufacturer’s instructions (Thermo Scientific). LC-MS/MS analysis was performed on a Q Exactive mass spectrometer (Thermo Scientific) that was coupled to Easy nLC (Proxeon Biosystems, now Thermo Fisher Scientific) for 60/90 min. The MS raw data for each sample were searched using the MASCOT engine (Matrix Science, London, UK; version 2.2) embedded into Proteome Discoverer 1.4 software for identification and quantitation analysis. The proteomics analysis, including protein digestion, TMT labelling, fractionation, LC-MS/MS analysis, protein identification, and protein quantitation was performed by Shanghai Applied Protein Technology Co., Ltd. (MS).

RNA sequencing

For RNA sequencing, the libraries were generated using NEBNext® UltraTM RNA Library Prep Kit for Illumina® (NEB, USA) following manufacturer’s recommendations. The clustering of the index-coded samples was performed on a cBot Cluster Generation System using TruSeq PE Cluster Kit v3-cBot-HS (Illumia). After cluster generation, the library preparations were sequenced on an Illumina Novaseq platform and 150 bp paired-end reads were generated. Reads were mapped using Hisat2 v2.0.5 and analyzed by featureCounts v1.5.0-p3. Raw data were deposited in gene expression omnibus (GEO) under accession codes GSE176151.

Western blotting

Denatured cells lysates and liver tissues homogenate were fresh prepared and processed with Bio-Rad Gel Electrophoresis Systems (Bio-Rad). Proteins were transferred to PVDF membranes, and incubated with indicated antibodies. Protein bands were visualized using a chemiluminescence kit (Millipore, MA) and quantitated by densitometry using Image J software.

Immunohistochemistry (IHC) analysis

Paraffin sections of HCC tumor tissues were cut at 4 μm. After the slides were deparaffinized in xylene and a graded ethanol series. Heat-induced antigen retrieval and blocked with goat serum were carried out. The sections were then incubated with indicated antibodies. ABC Peroxidase Staining Kit (Thermo Fisher Scientific, USA) and DAB Detection kit were used to visualize indicated proteins. Finally, slides were counterstained with Hematoxylin.

Immunofluorescence (IF) analysis

Cells were plated on coverslips and treated as indicated. Following treatment, the cells were fixed in ice-cold methanol and permeabilized in 0.1% Triton X-100. Immunofluorescence was performed using standard techniques, and the immunostained cells were visualized with a laser scanning confocal microscope (Leica TCS SP8, Solms, Germany).

CRISPR-Cas9 system

The CRISPR/Cas9 plasmids lentiCRISPR-v2, pMD2.G, and psPAX2 are kind gifts from Prof. Ding Xue, School of Life Sciences, Tsinghua University, Beijing, China. The E-CRISP online tool (http://www.e-crisp.org/ECRISP/designcrispr.html) was used to design the HDAC2/PLEKHG5-targeting sequences. The LentiCRISPR v2 vector containing HDAC2 or PLEKHG5 sgRNA sequence, envelope plasmid pMD2.G, and packaging plasmid psPAX2 were cotransfected into HEK293T cells to generate lentiviruses using Lipofectamine 8000 (Beyotime, Shanghai, China) according to the manufacturer’s introduction. HCC cells were infected with the lentivirus, followed by puromycin selection to establish stable knockout cell lines. Single-cell clones stably expressing sgRNA were cultured and verified via DNA sequencing and western blotting.

Plasmid DNA construction and transfection

The pSEB-C3F plasmid was kindly provided by Dr T-C He, University of Chicago, USA. To construct a Flag-labeled PLEKHG5 plasmid, PLEKHG5 was amplified by PCR and cloned into the expression vector. The plasmid containing a K-R or K-Q mutation in the PH domain of PLEKHG5 was constructed in the same way. For overexpression, the specified plasmid was transfected instantaneously into cells using Lipofectamine 8000 reagent. Forty-eight hours after transfection, cells were collected for further applications. Primers are listed in supplementary table 2.

Coimmunoprecipitation (Co-IP)

Cells transfected with pSEB‐PLEKHG5‐3Flag or control vector were resuspended in lysis buffer (Beyotime, Shanghai, China) after indicated treatments. The Pre-cleared extracts were incubated overnight with protein A/G magnetic beads coupled with primary antibodies. Immunoprecipitates were eluted in 2× Laemmli buffer and then subjected to immunoblotting analysis using the indicated antibodies.

Immunoprecipitation-mass spectrometry (IP-MS)

PLEKHG5 was immunoprecipitated from MHCC97H cells transfected with PLEKHG5-Flag and subjected to 8% SDS-PAGE for Coomassie blue staining. Mass spectrometry analysis of PLEKHG5 acetylation was performed by Shanghai Applied Protein Technology Co., Ltd. (MS).

HDAC2 enzyme activity

HDAC2 was immunoprecipitated from MHCC97H, MHCC97H SR, PLC/PRF/5, and PLC/PRF/5 SR cells. HDAC2 protein was eluted from protein A/G magnetic beads method by non-denaturing elution method. Activity of HDAC2 was determined using HDAC Activity Assay Kit (Abnova, USA) according to the manufacture’s protocol. The experiment was repeated at least three times.

**Supplementary Figure Legends**


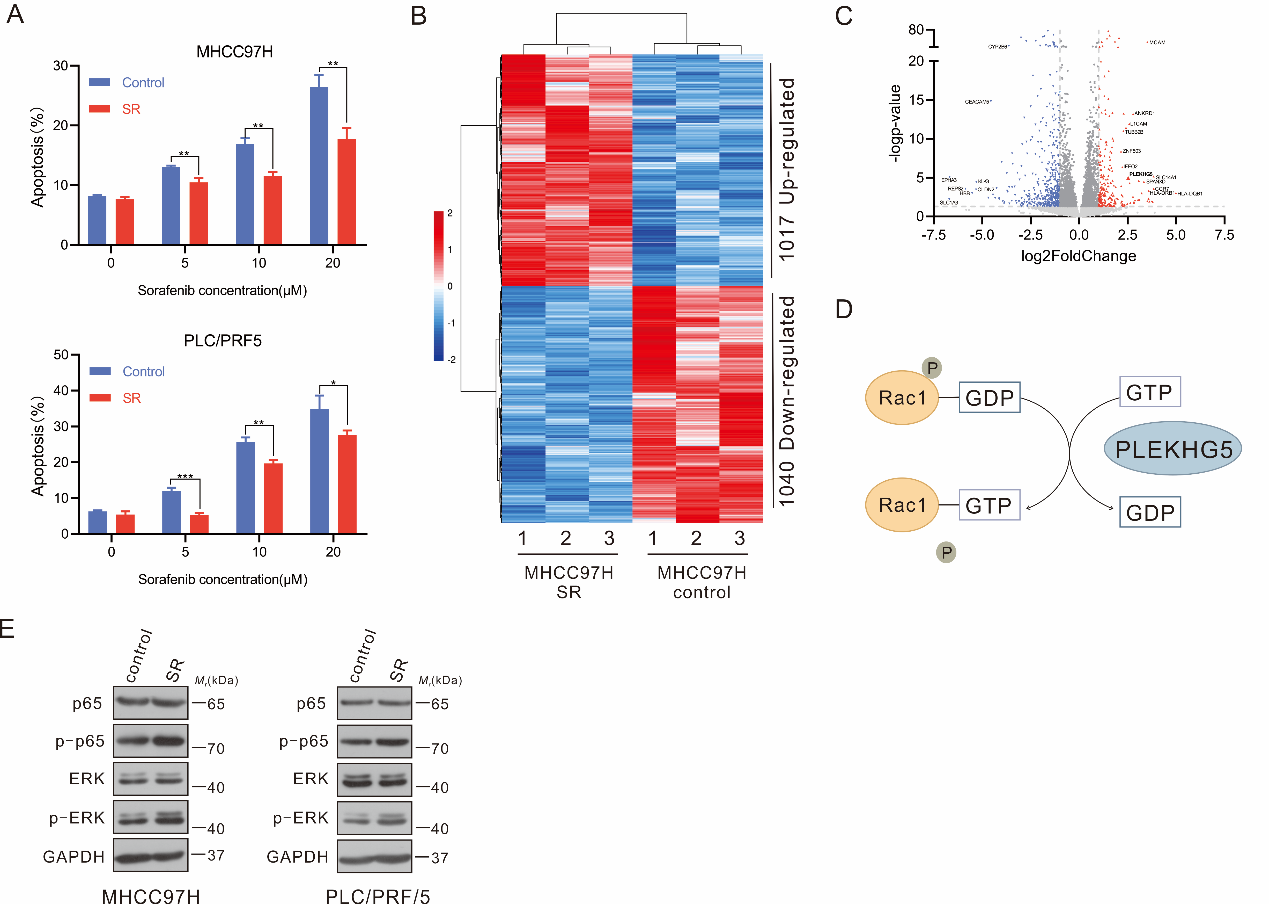


Figure S1: PLEKHG5 is upregulated in sorafenib-resistant HCC cells.

A: Apoptosis of MHCC97H, MHCC97H SR, PLC/PRF/5 and PLC5/PRF/5 SR cells after 48h treating with different doses of Sorafenib detected by annexin V staining followed with flow cytometry assay. B: Heat map of the DEGs in MHCC97H and MHCC97H-SR cells. C: Volcano plot of RNA-sequencing data obtained from MHCC97H SR and MHCC97H cells. D: Schematic diagram of the regulation of Rac1 activation by PLEKHG5. E: Western blot analysis of the protein levels of p65, p-p65, ERK1/2(ERK), phosphorylated ERK1/2(p-ERK) in sorafenib sensitive and resistant cells. GAPDH served as the loading control.

Error bars represent the mean ± SD, *P < 0.05, **P < 0.01, ***P < 0.001, ****P < 0.0001, One-way ANOVA analysis.


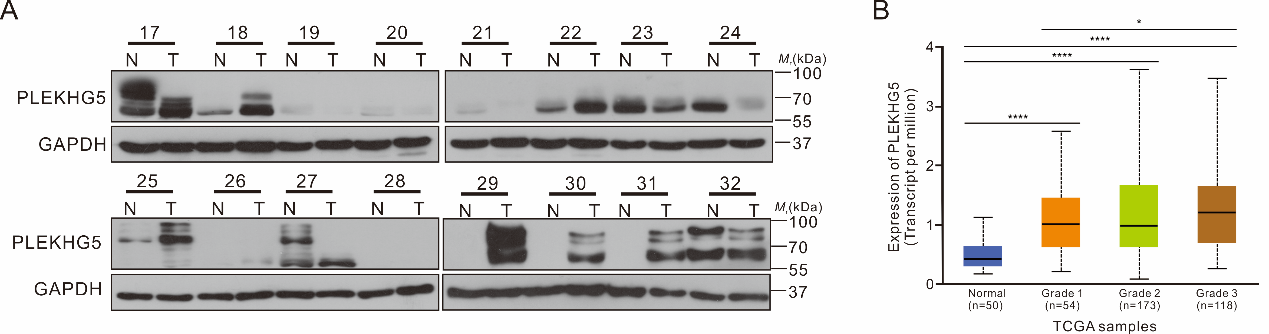


Figure S2: PLEKHG5 is upregulated in HCC and correlated with the prognosis of HCC patients.

A: Western blot analysis of PLEKHG5 protein levels in HCC patients. GAPDH served as the loading control. B: PLEKHG5 mRNA levels in different grades of HCC from TCGA-LIHC dataset.

Error bars represent the mean ± SD, *P < 0.05, **P < 0.01, ***P < 0.001, ****P < 0.0001, One-way ANOVA analysis.


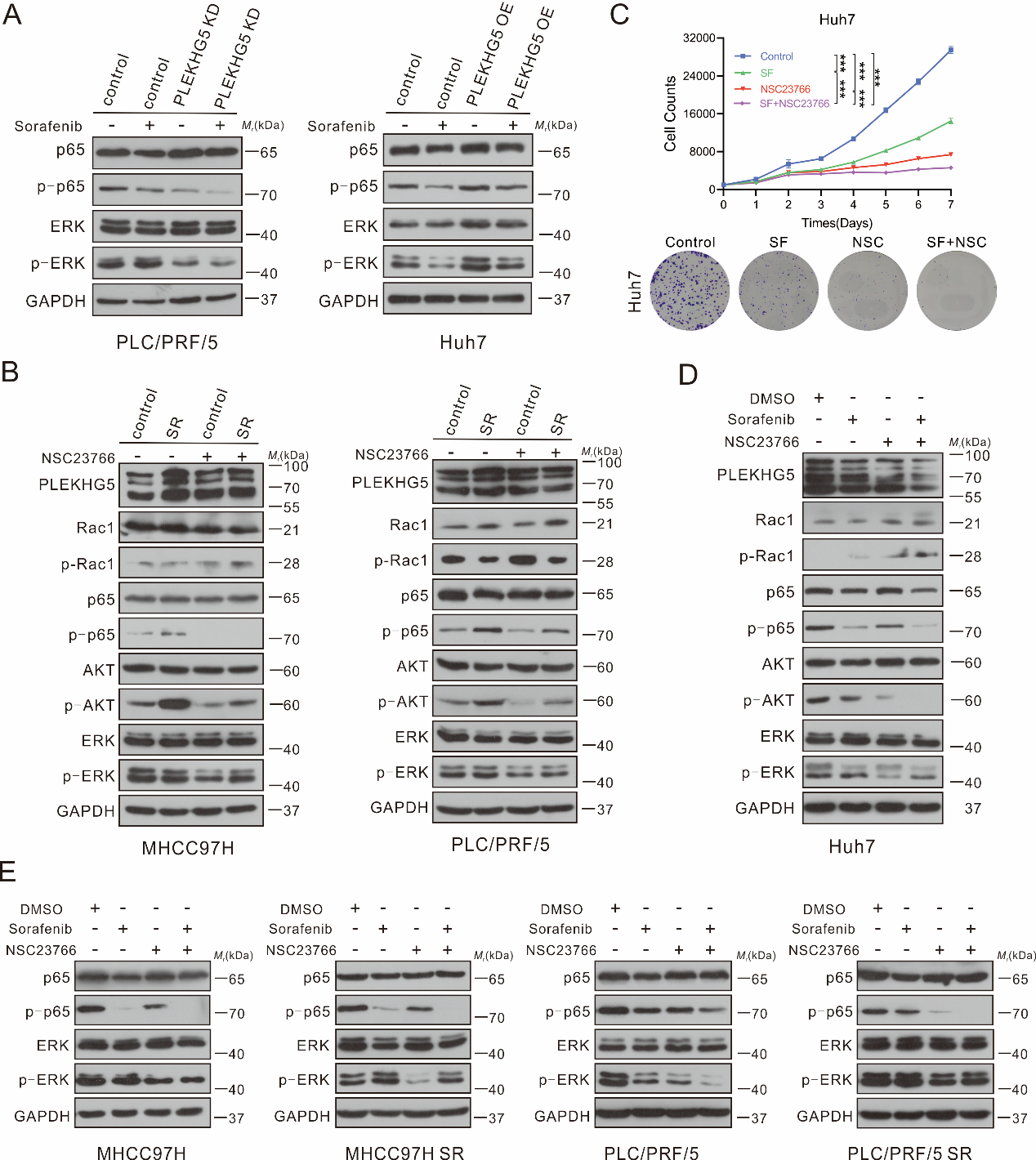


Figure S3: PLEKHG5 expression correlates with HCC cell growth and promotes HCC cell sorafenib resistance.

A: Western blot analysis of the protein levels of p65, p-p65, ERK and p-ERK in PLC/PRF/5 PLEKHG5-OE and Huh7 PLEKHG5-KD cells treated with/without Sorafenib for 48 hours. GAPDH served as the loading control. B: Western blot analysis of the protein levels of PLEKHG5, Rac1, p-Rac1, p65, p-p65, AKT, p-AKT, ERK and p-ERK in sorafenib sensitive or resistant cells treated with/without NCS23766 for 48 hours. GAPDH served as the loading control. C: MTS assay and colony formation assays of Huh7 cells treated with Sorafenib and/or NSC23766. D: Western blot analysis of the protein levels of PLEKHG5, Rac1, p-Rac1, p65, p-p65, AKT, p-AKT, ERK and p-ERK in Huh7 cells treated with/without NCS23766 for 48 hours. GAPDH served as the loading control. E: Western blot analysis of the protein levels of p65, p-p65, ERK and p-ERK in sorafenib sensitive or resistant cells after treating with sorafenib and/or NSC23766.GAPDH served as the loading control.
Error bars represent the mean ± SD from the biological triplicates, *P < 0.05, **P < 0.01, ***P < 0.001, ****P < 0.0001, One-way ANOVA analysis.


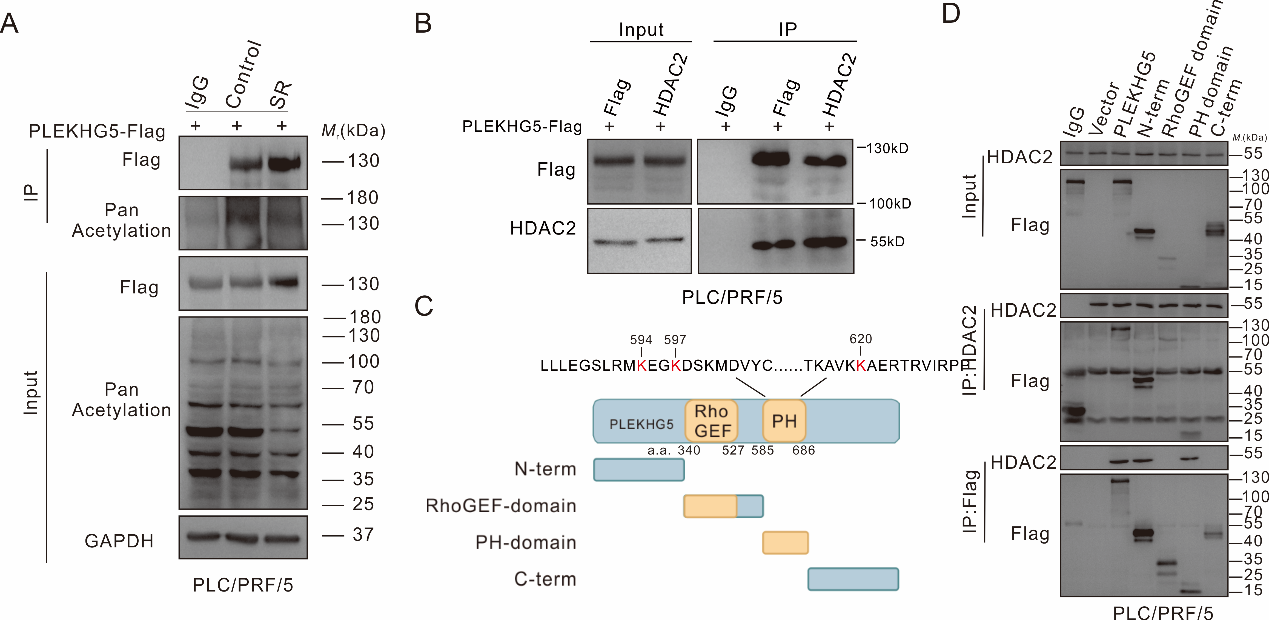


Figure S4: HDAC2 interacts with PLEKHG5 and associates with its acetylation.

A: IB analysis of WCL and anti-Flag IP derived from PLC/PRF/5 sorafenib sensitive and resistant cells transfected with PLEKHG5-Flag. B: Co-IP analysis of HDAC2 and PLEKHG5-Flag in WCL of PLC/PRF/5 cells transfected with PLEKHG5-Flag. C: Schematic of Flag-tagged PLEKHG5 domains. D: Co-IP assay of HDAC2 and Flag in WCL of PLC/PRF/5 cells transfected with indicated PLEKHG5 peptide Flag (PLEKHG5-Flag-WT, N-term-Flag, RhoGEF domain-Flag, PH domain-Flag or C-term-Flag).


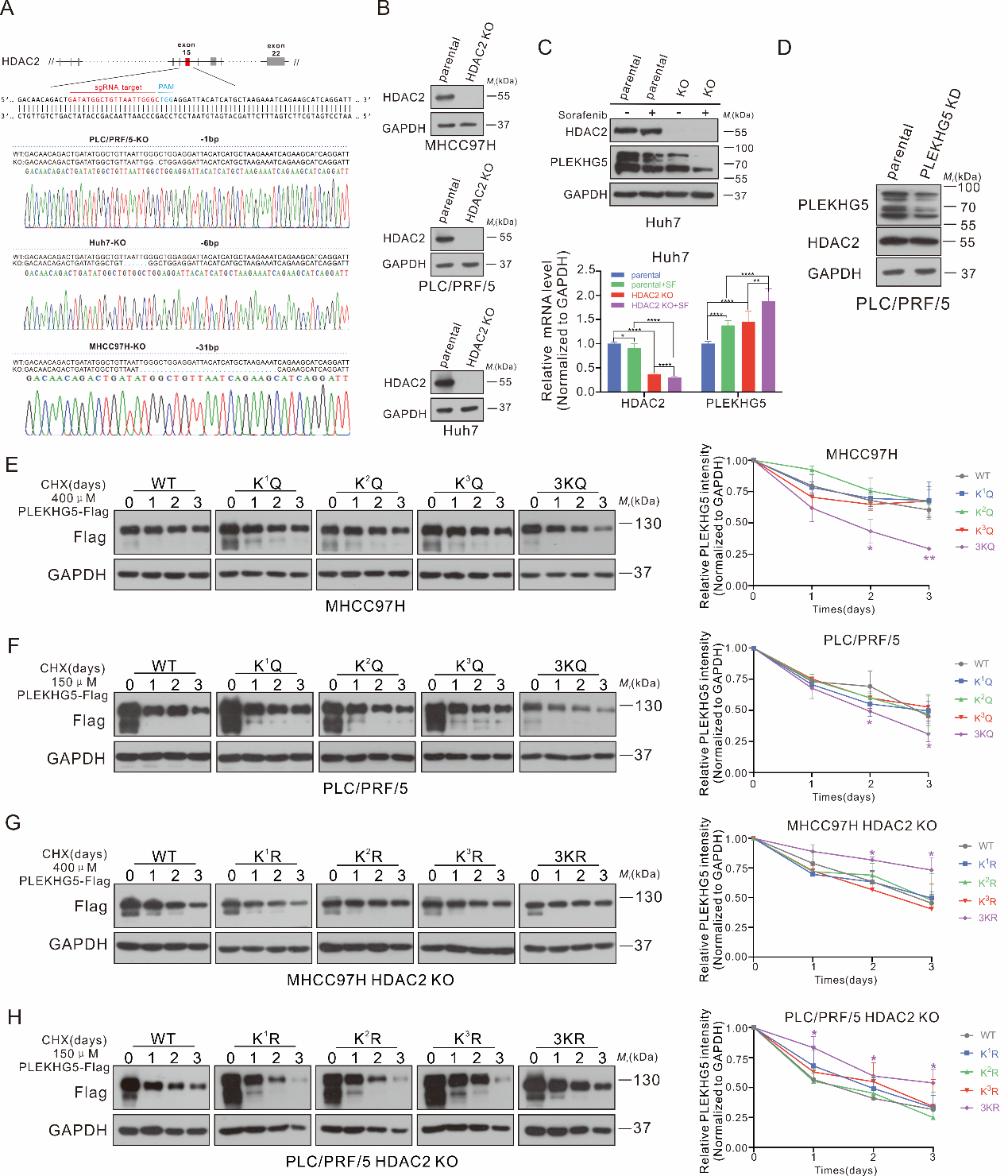


Figure S5: HDAC2 deacetylates PLEKHG5 and maintains its protein stability.
A: HDAC2 genome sequencing of MHCC97H, PLC/PRF/5 and Huh7 HDAC2 KO cells. B: Western blot analysis of HDAC2 protein levels in different HDAC2 KO cells. C: Western blot analysis and qRT-PCR analysis of the levels of HDAC2 and PLEKHG5 in Huh7 parental and HDAC2 KO cells treated with or without sorafenib for 48 hours. GAPDH served as the loading control. D: Western blot analysis of the protein levels of PLEKHG5 and HDAC2 in PLC/PRF/5 parental and PLEKHG5-KD cells. GAPDH served as the loading control. E-F: Western blot analysis of the protein level of PLEKHG5-Flag in MHCC97H and PLC/PRF/5 cells transfected with PLEKHG5-FLAG or its K to Q mutant plasmids for 48 hours. Cycloheximide (CHX) was used to block protein synthesis before cells were harvested. Relative protein intensity of PLEKHG5 was quantified by ImageJ. GAPDH served as the loading control. G-H: Western blot analysis of the protein level of PLEKHG5-Flag in HDAC2 knockout cells transfected with PLEKHG5-Flag or its K to R mutant plasmids for 48 hours. Cycloheximide (CHX) was used to block protein synthesis before cells were harvested. Relative protein intensity of PLEKHG5 was quantified by ImageJ. GAPDH served as the loading control.

Error bars represent the mean ± SD from the biological triplicates, *P < 0.05, **P < 0.01, ***P < 0.001, ****P < 0.0001, One-way ANOVA analysis.


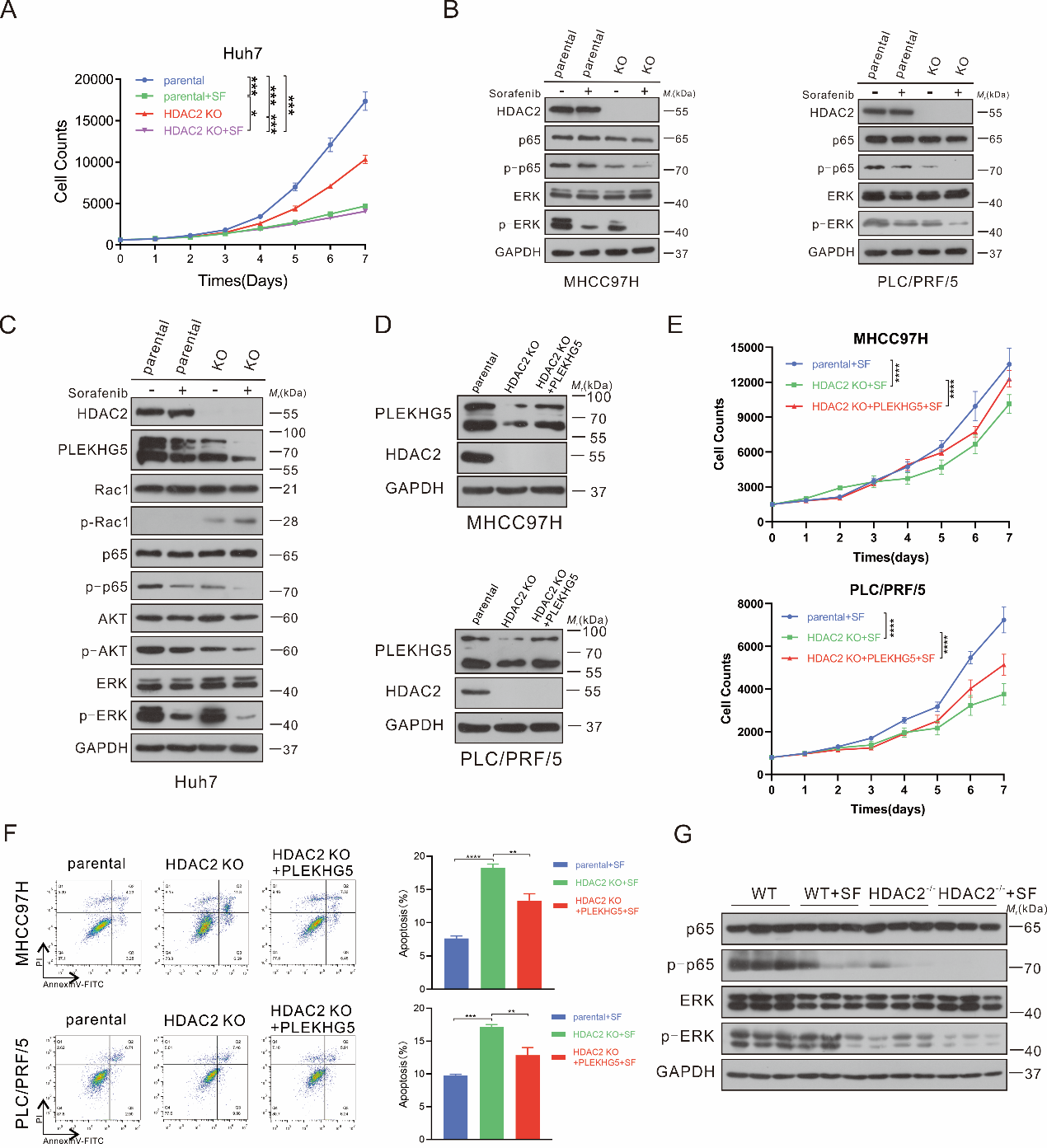


Figure S6: Knockout of HDAC2 enhances the sensitivity of HCC to sorafenib in vitro and in vivo.

A: Continuous cell counts of Huh7 parental and HDAC2 KO cells treated with/without sorafenib by MTS assay. B: Western blot analysis of the protein levels of HDAC2, p65, p-p65, ERK and p-ERK in MHCC97H, PLC/PRF/5 parental and HDAC2 KO cells treated with or without sorafenib for 48 hours. GAPDH served as the loading control. C: Western blot analysis of the protein levels of HDAC2, PLEKHG5, Rac1, p-Rac1, AKT, p-AKT, p65, p-p65, ERK and p-ERK in Huh7 parental and HDAC2 KO cells treated with or without sorafenib for 48 hours. GAPDH served as the loading control. D: Western blot analysis of the protein levels of HDAC2 and PLEKHG5 in MHCC97H, PLC/PRF/5 parental, HDAC2 KO and HDAC2 KO overexpressing PLEKHG5 cells. GAPDH served as the loading control. E: Continuous cell counts of parental, HDAC2 KO and HDAC2 KO overexpressing PLEKHG5 cells treated with sorafenib by MTS assay. F: Apoptosis of parental, HDAC2 KO and HDAC2 KO overexpressing PLEKHG5 cells after 48h treating with Sorafenib by annexin V staining followed with flow cytometry assay. G: Western blot analysis of the protein levels of p65, p-p65, ERK, and p-ERK in each group from (Fig.6C). GAPDH served as the loading control.

Error bars represent the mean ± SD from the biological triplicates, *P < 0.05, **P < 0.01, ***P < 0.001, ****P < 0.0001, One-way ANOVA analysis.


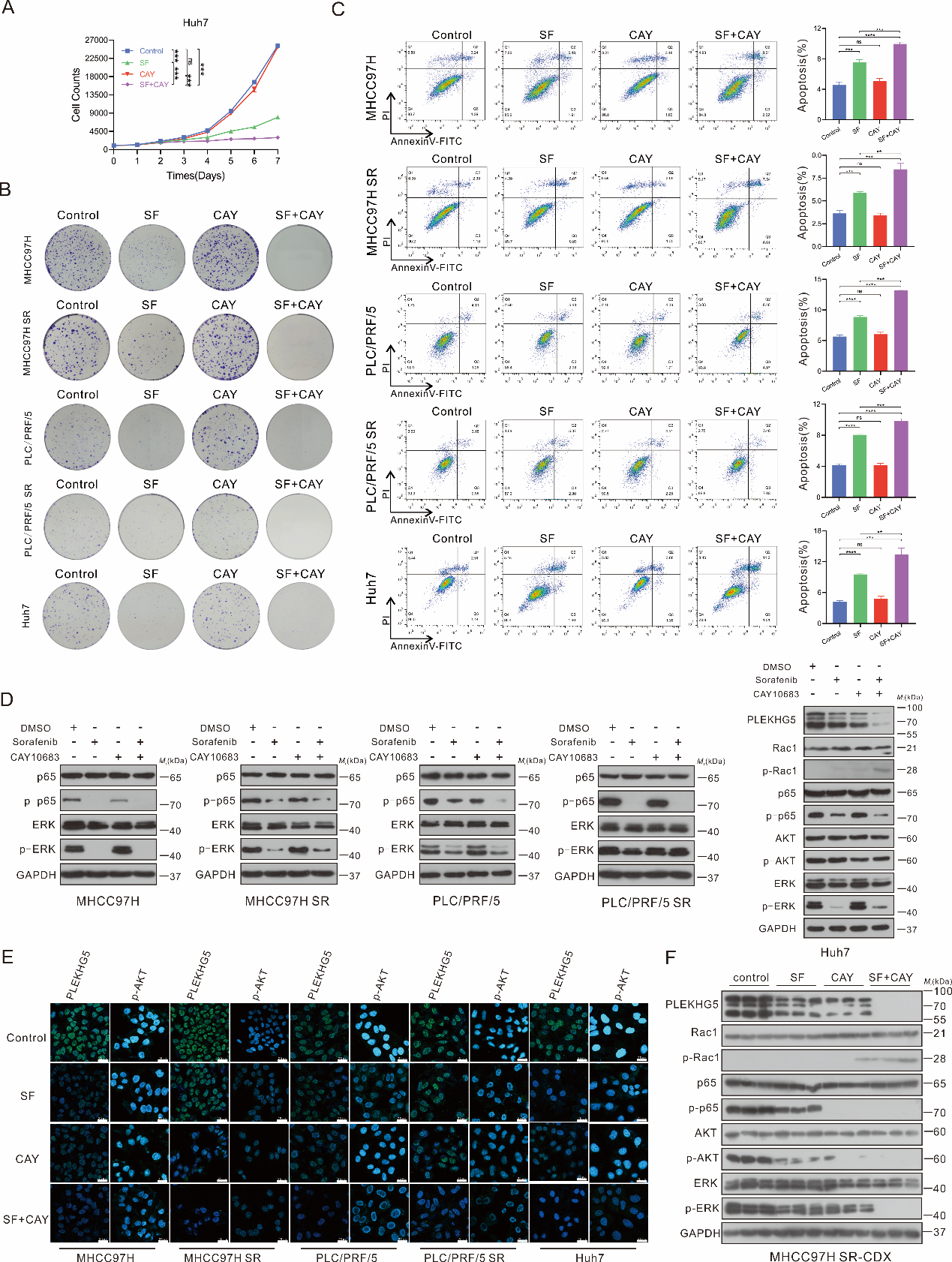


Figure S7: Selective HDAC2 inhibition attenuates sorafenib resistance in HCC.

A: Continuous cell counts of Huh7 cells treated with sorafenib and/or CAY10683 by MTS assay. B: Colony formation assays of MHCC97H, MHCC97H SR, PLC/PRF/5, PLC/PRF/5 SR and Huh7 cells treated with Sorafenib and/or CAY10683. C: Apoptosis of PLC/PRF/5, PLC5/PRF/5 SR and Huh7 cells after 48h treating with Sorafenib and/or CAY10683 by annexin V staining followed with flow cytometry assay. D: Western blot analysis of the protein levels of PLEKHG5, Rac1, p-Rac1, AKT, p-AKT, p65, p-p65, ERK and p-ERK in MHCC97H, MHCC97H SR, PLC/PRF/5, PLC/PRF/5 SR and Huh7 cells treated with sorafenib and/or CAY10683 for 48 hours. GAPDH served as the loading control. E: IF analysis of the protein levels of PLEKHG5 and p-AKT in MHCC97H, MHCC97H SR, PLC/PRF/5, PLC/PRF/5 SR and Huh7 cells treated with sorafenib and/or CAY10683 for 48 hours. Bar represents 25 μM. F: Western blot analysis of the protein levels of PLEKHG5, Rac1, p-Rac1, p65, p-p65, AKT, p-AKT, ERK and p-ERK in each group from MHCC97H SR-CDX.

Error bars represent the mean ± SD from the biological triplicates, *P < 0.05, **P < 0.01, ***P < 0.001, ****P < 0.0001, One-way ANOVA analysis.

**Supplementary Tables**

Supplemental table 1: list of reagents and antibodies

| Catalogue | Antibody Name | Antibody species | MW（KD） | Company | Application and Radio |
| --- | --- | --- | --- | --- | --- |
| TA-08 | GAPDH | Mouse | 37 | ZSGB-BIO | WB 1:2000 |
| #4691 | AKT | Rabbit | 60 | CST | WB 1:1000 |
| #4060 | Phospho-AKT(Ser473) | Rabbit | 60 | CST | WB 1:2000  IHC 1:100 |
| #4695 | p44/42 MAPK  (Erk 1/2) | Rabbit | 42,44 | CST | WB 1:1000 |
| #4370 | Phospho-p44/42 MAPK (Thr202/Tyr204) | Rabbit | 42,44 | CST | WB 1:2000 |
| ab19845 | HDAC1 | Rabbit | ~62 | Abcam | WB 1:1000  IP:3μg |
| ab12169 | HDAC2 | Mouse | 55 | Abcam | WB 1:4000  IHC 1:100 IP：3μg |
| ab7030 | HDAC3 | Rabbit | 49 | Abcam | WB 1:1000  IP:3μg |
| ab12172 | HDAC4 | Rabbit | ~140 | Abcam | WB 1:1000  IP:3μg |
| ab32441 | SIRT1 | Rabbit | ~120 | Abcam | WB 1:10000  IP:3μg |
| Ab32536 | NF-κB p65 | Rabbit | 65 | Abcam | WB 1:10000 |
| ab86299 | NF-kB p65 (phospho S536) | Rabbit | 65 | Abcam | WB 1:2000  IHC 1:500 |
| #4651 | Rac1/cdc42 | Rabbit | 21 | CST | WB 1:1000 |
| #2461 | Phospho-Rac1/cdc42(Ser71) | Rabbit | 28 | CST | WB 1:1000 |
| #PA5-16785 | Ki67 | Mouse |  | invitrogen | IHC 1:200 |
| LS-B16755 LS-C133796 | PLEKHG5 | Mouse |  | LifeSpan BioScience,Inc LSBio | IHC 1:500(1:100) |
| MA1-91878 | Flag | Mouse |  | invitrogen | WB 1:1000 |
| STJ111149 | Anti-Pan Acetyl-Lysine antibody | Rabbit |  | St John's Laboratory | WB 1:1000 |
| #9441 | Acetylated-Lysine Antibody | Rabbit |  | CST | WB 1:1000  IP:3μg |
| 211-032-171 | Anti-Rabbit IgG, light chain specific | Mouse |  | Jackson ImmunoResearch |  |
| 115-035-174 | Anti-Mouse IgG, light chain specific | Goat |  | Jackson ImmunoResearch |  |
| S7397 | Sorafenib |  |  | selleck |  |
| S7595 | Santacruzamate A(CAY10683) |  |  | selleck |  |
| S8031 | NSC23766 |  |  | selleck |  |
| S7418 | Cycloheximide (CHX) |  |  | selleck |  |
| S3020 | Romidepsin  (FK228) |  |  | selleck |  |
| S1047 | Vorinostat (SAHA) |  |  | selleck |  |
| N0636 | Nicotinamide (NAM) |  |  | Sigma-Aldrich |  |
| N0756 | Diethylnitrosamine  (DEN) |  |  | Sigma-Aldrich |  |
| C805332 | carbon tetrachloride  (CCL4) |  |  | MACKLIN |  |
| C0005 | Cell Counting Kit-8 (CCK-8) |  |  | TOPSCIENCE |  |

Supplemental table 2: list of primers

| Primer Name | | | | Sequence (5′-3′) | |  |
| --- | --- | --- | --- | --- | --- | --- |
| Human GAPDH qPCR | | forward | | | GACATCAAGAAGGTGGTGAA |  |
|  |  | reverse | | | TGTCATACCAGGAAATGAGC |  |
| Human HDAC2 qPCR | | forward | | | ATATTGTGCTTGCCATCC |  |
|  |  | reverse | | | CCTCAAGTCTCCTGTGCC |  |
| Human PLEKHG5 qPCR | | forward | | | CGATGAAGACGAGGATGAG |  |
|  |  | reverse | | | CACAGGAACAGGTTGATGA |  |
| HDAC2 KO | sgRNA target Forward | | | | CACCGATATGGCTGTTAATTGGGC |  |
|  | sgRNA target Reverse | | | | AAACGCCCAATTAACAGCCATATC |  |
| HDAC2 TA-clone | | TA-Forward | | | GGCGGTTCAGTTGGTAA |  |
|  |  | TA-Reverse | | | AAGGATGGCAAGCACAA |  |
| PLEKHG5 KD | | sgRNA target Forward | | | CACC GCGGCTTCCTCCGGCCGCCA |  |
|  |  | sgRNA target Reverse | | | AAAC TGGCGGCCGGAGGAAGCCGC |  |
| pSEB-PLEKHG5-Flag | | forward | | | CCCAAGCTT ACC ATG GGC CATTATGATGGGCATGTC |  |
|  |  | reverse | | | CGCGGATCC AGCAGCAGGGTGGTCCTGA |  |
| K^1^R | | forward | | | GATGAAGGAGGGGAGGGACAGCAAGATG |  |
|  |  | reverse | | | CATCTTGCTGTCCCTCCCCTCCTTCATC |  |
| K^2^R | | forward | | | CAAAGCAGTGAAGAGGGCAGAGAGGACC | |
|  |  | reverse | | | GGTCCTCTCTGCCCTCTTCACTGCTTTG | |
| K^3^R | | forward | | | GAGCCTGAGGATGAGAGAGGGGAAGGACAG | |
|  |  | reverse | | | CTGTCCTTCCCCTCTCTCATCCTCAGGCTC | |
| K^1^Q | | forward | | | GAGCCTGAGGATGCAGGAGGGGAAGGACAG | |
|  |  | reverse | | | CTGTCCTTCCCCTCCTGCATCCTCAGGCTC | |
| K^2^Q | | forward | | | GATGAAGGAGGGGCAGGACAGCAAGATG | |
|  |  | reverse | | | CATCTTGCTGTCCTGCCCCTCCTTCATC | |
| K^3^Q | | forward | | | CAAAGCAGTGAAGCAGGCAGAGAGGACC | |
|  |  | reverse | | | GGTCCTCTCTGCCTGCTTCACTGCTTTG | |
| N-term | | forward | | | AAATATGCGGCCGCGCCACCATGCATTATGATGGGCATGTCC | |
|  |  | reverse | | | CGC GGATCC CTCCTGCTGGTGGCACTG | |
| Rho GEF domain | | forward | | | AAATATGCGGCCGCGCCACCATGGCGGTGTGGGAGCTGCTGCAC | |
|  |  | reverse | | | CGCGGATCCCTGCCGCGTCTCCTCCGG | |
| PH domain | | forward | | | AAATATGCGGCCGCGCCACCATGCTGCTGCTGGAGGGGAGCC | |
|  |  | reverse | | | CGCGGATCCCAGCTGGTTCTGGGCATTG | |
| C-term | | forward | | | AAATATGCGGCCGCGCCACCATGCAACAGCTGCGTGCACAGG | |
|  |  | reverse | | | CGCGGATCCGTAAGCAGCAGGGTGGTCC | |
| Human ABR qPCR | | forward | | | TTCCTGCTGCTGCTCAACT | |
|  |  | reverse | | | TCTCCACTCTGACCTCTCGTA | |
| Human ARHGEF4 qPCR | | | forward | | CAGTTGCTCTCACAGTCAGAAG | |
| Human ARHGEF4 qPCR | | | reverse | | GCCACAGGTCATCCACACT | |
| Human NET1 qPCR | | forward | | | TGATGGAACAGTGGAGCAGAT | |
|  |  | reverse | | | ATCGCTGGAGGAAGTCTTGG | |
| Human DNMBP qPCR | | forward | | | GAACCGCTGGCTGATTGAC | |
|  |  | reverse | | | CTCTGTGGAGGAGTGGCTAC | |
| Human ITSN1 qPCR | | forward | | | GGCAACAGGCATTGGAAGG | |
|  |  | reverse | | | CCAGGTCTCGGATGAAGAACT | |
| Human ITSN2 qPCR | | forward | | | CCGAGAAGAAGAAGCGTGAGA | |
|  |  | reverse | | | GATGGTCCTGGTGGTGTAGC | |
