# Supplementary figures and images for: PLEKHG5 is stabilized by HDAC2-related deacetylation and confers sorafenib resistance in hepatocellular carcinoma
Source: Cell Death Discov. 2023 May 29;9:176. doi: 10.1038/s41420-023-01469-z (PMC10227013; doi:10.1038/s41420-023-01469-z)

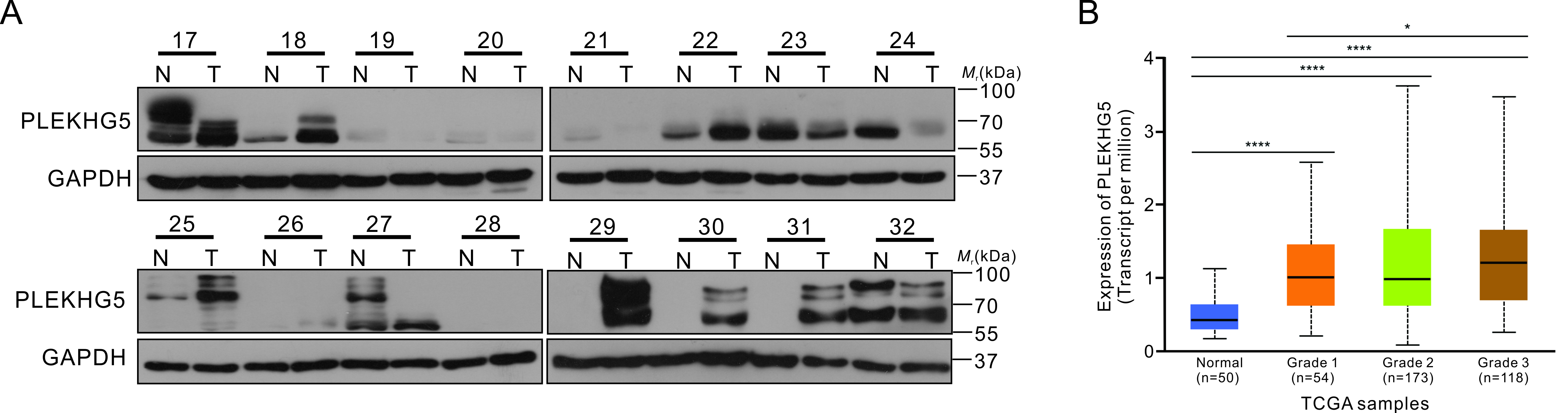

Supplement: Supplementary file 3 — Supplementary Figure S2 [file 41420_2023_1469_MOESM3_ESM.tif]

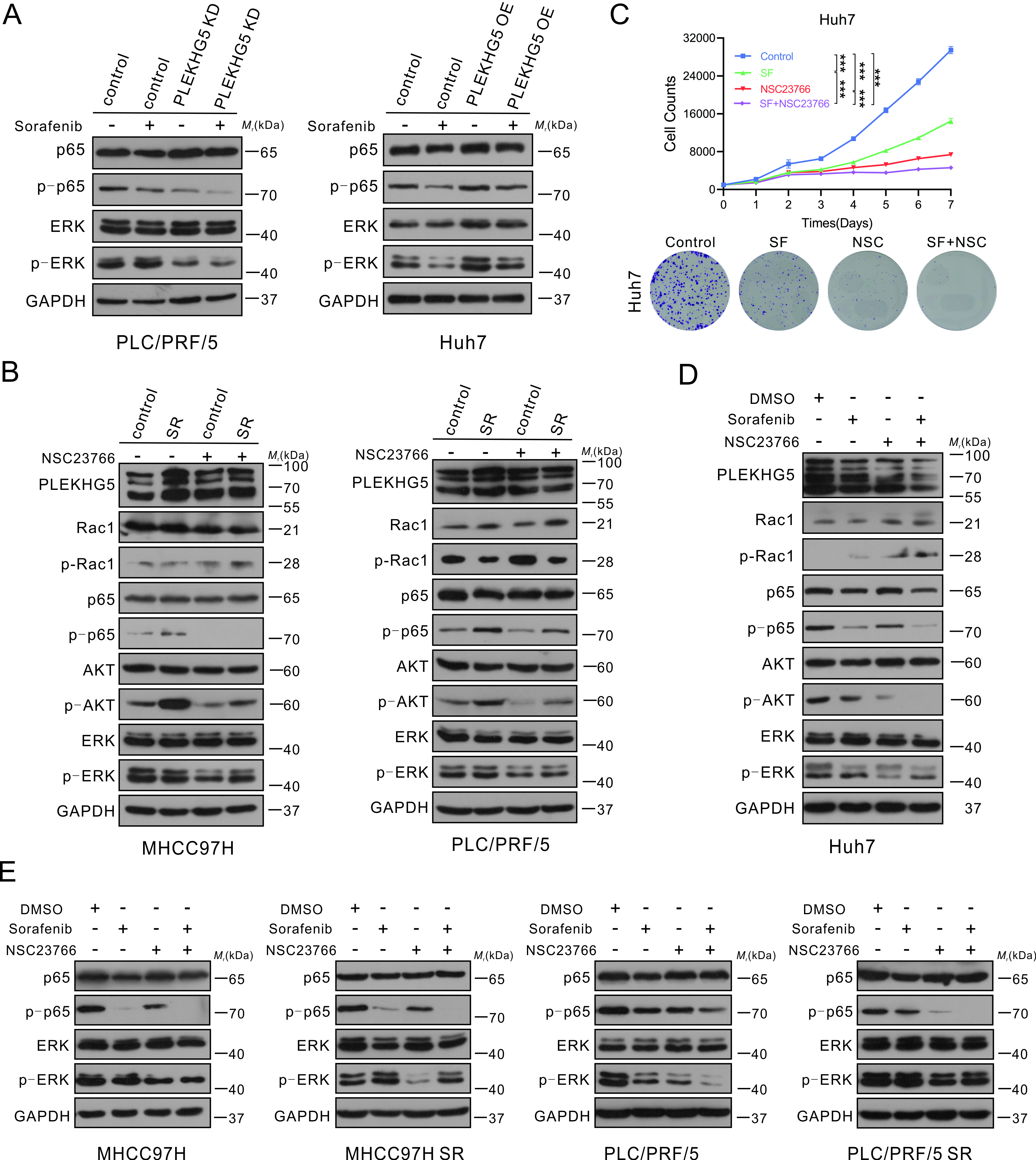

Supplement: Supplementary file 4 — Supplementary Figure S3 [file 41420_2023_1469_MOESM4_ESM.tif]

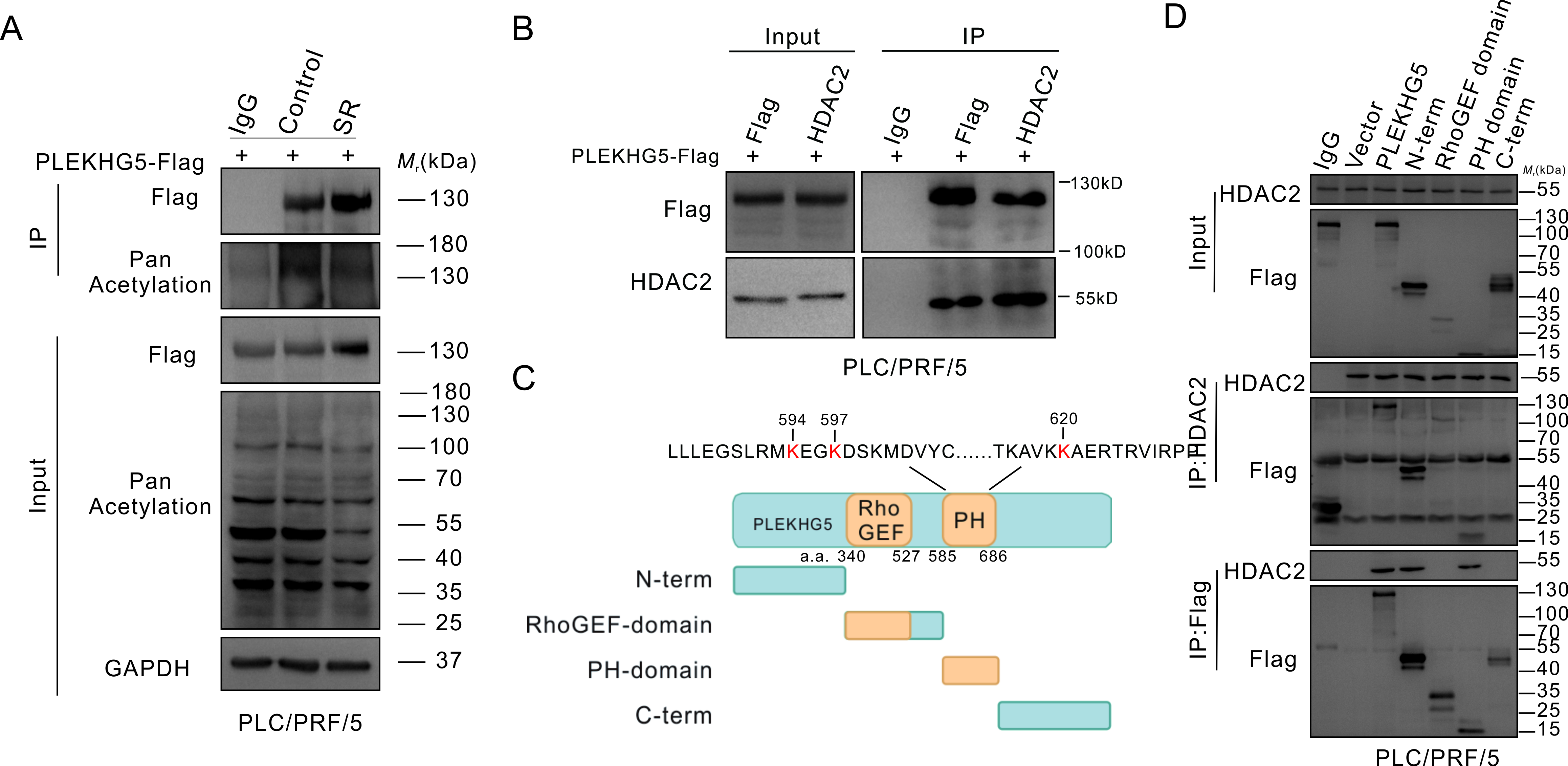

Supplement: Supplementary file 5 — Supplementary Figure S4 [file 41420_2023_1469_MOESM5_ESM.tif]
