# Supplementary material for: PLEKHG5 is stabilized by HDAC2-related deacetylation and confers sorafenib resistance in hepatocellular carcinoma
Source: Cell Death Discov. 2023 May 29;9:176. doi: 10.1038/s41420-023-01469-z (PMC10227013; doi:10.1038/s41420-023-01469-z)
Supplement: Supplementary file 9 — Western Blot [file 41420_2023_1469_MOESM9_ESM.pdf]

Figure 1K: MHCC97H

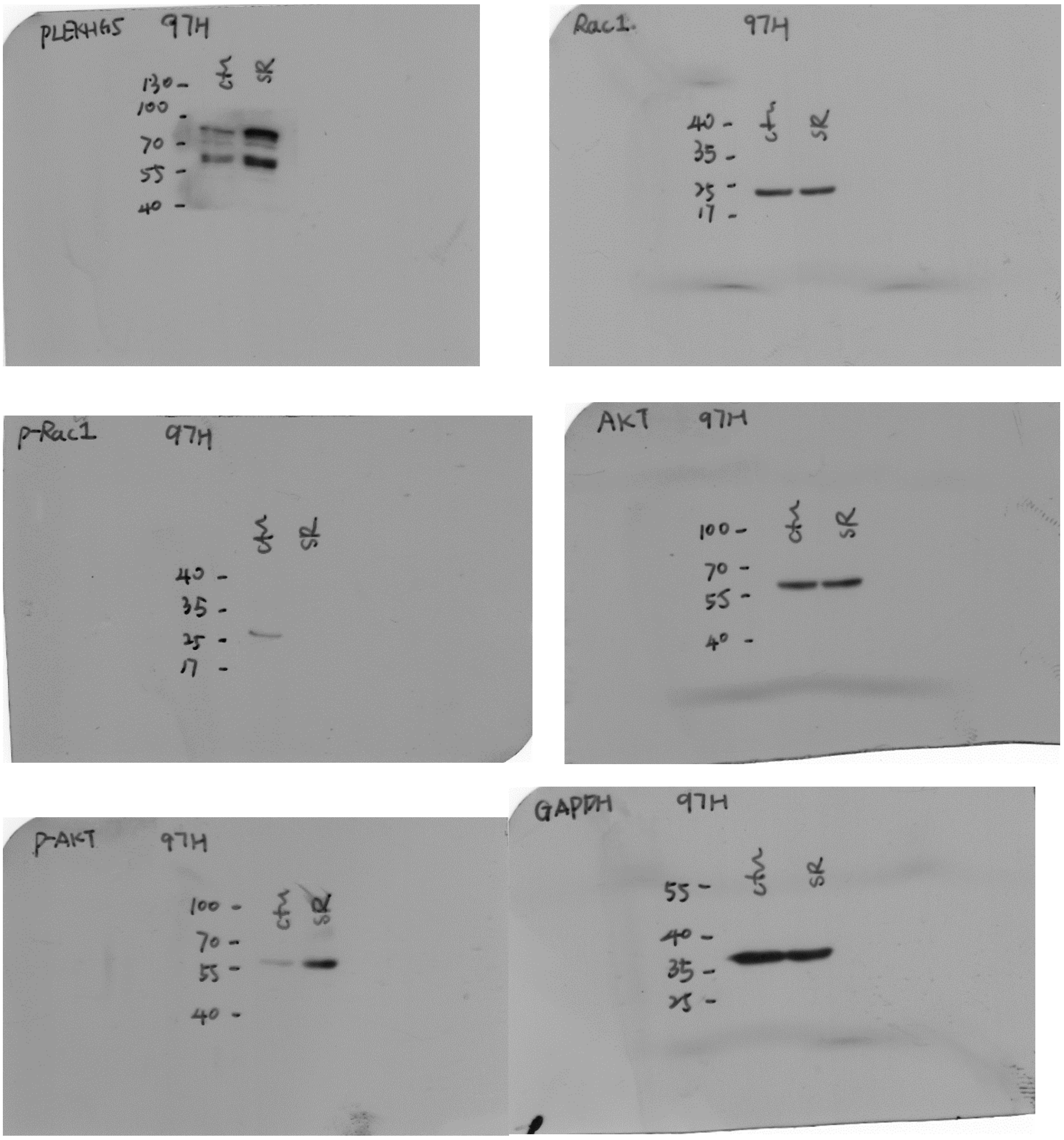

Figure 1L: PLC/PRF/5

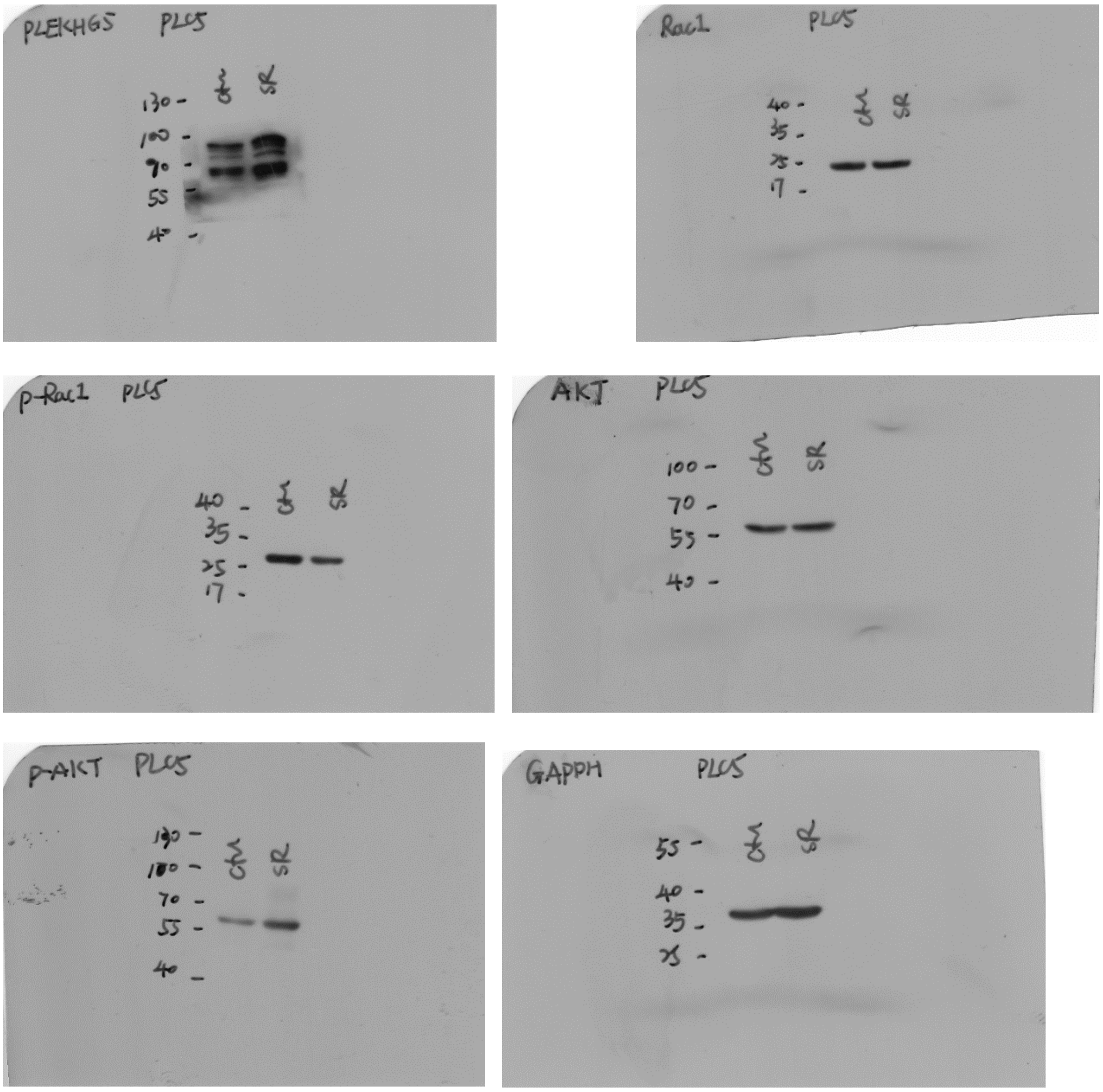

Figure 2C:

1-4:

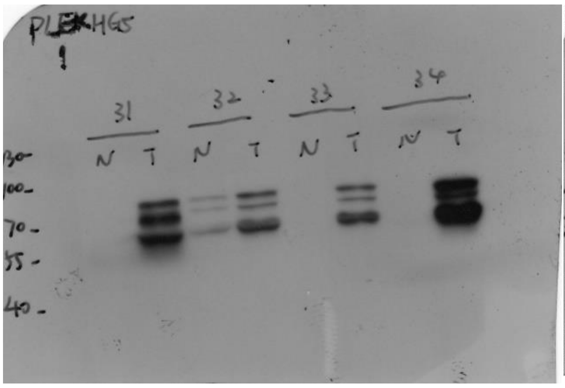

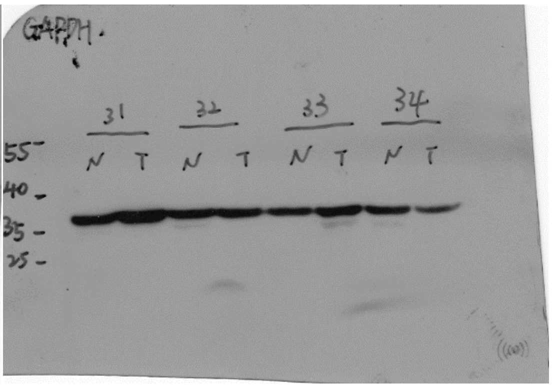

5-8:

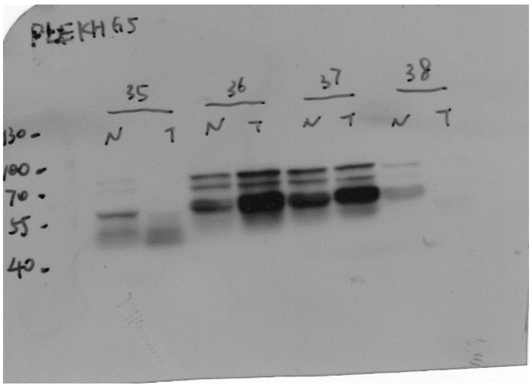

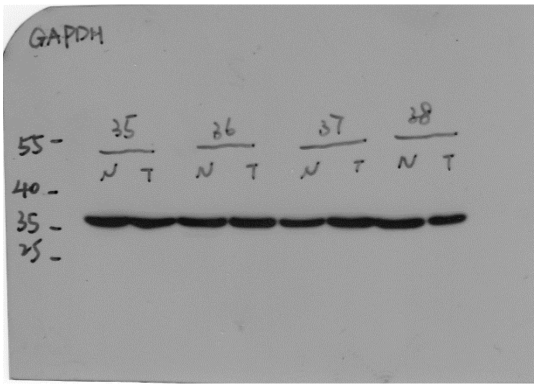

9-12

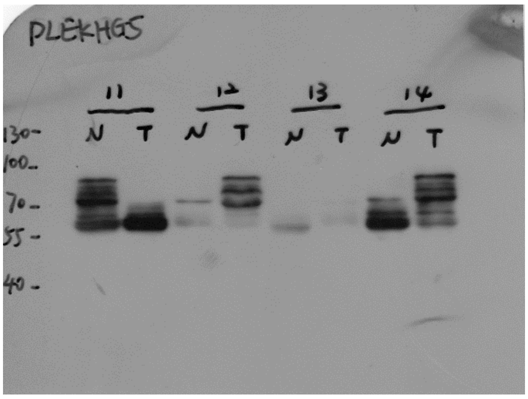

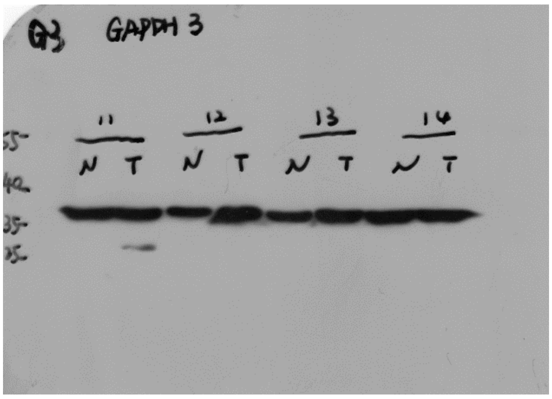

13-16

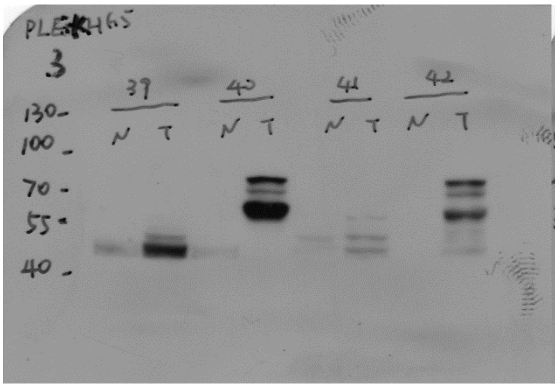

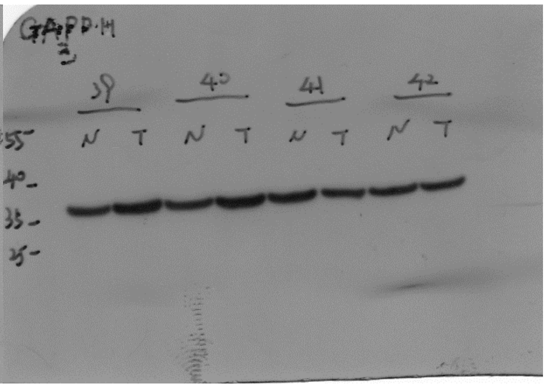

Figure 3A:

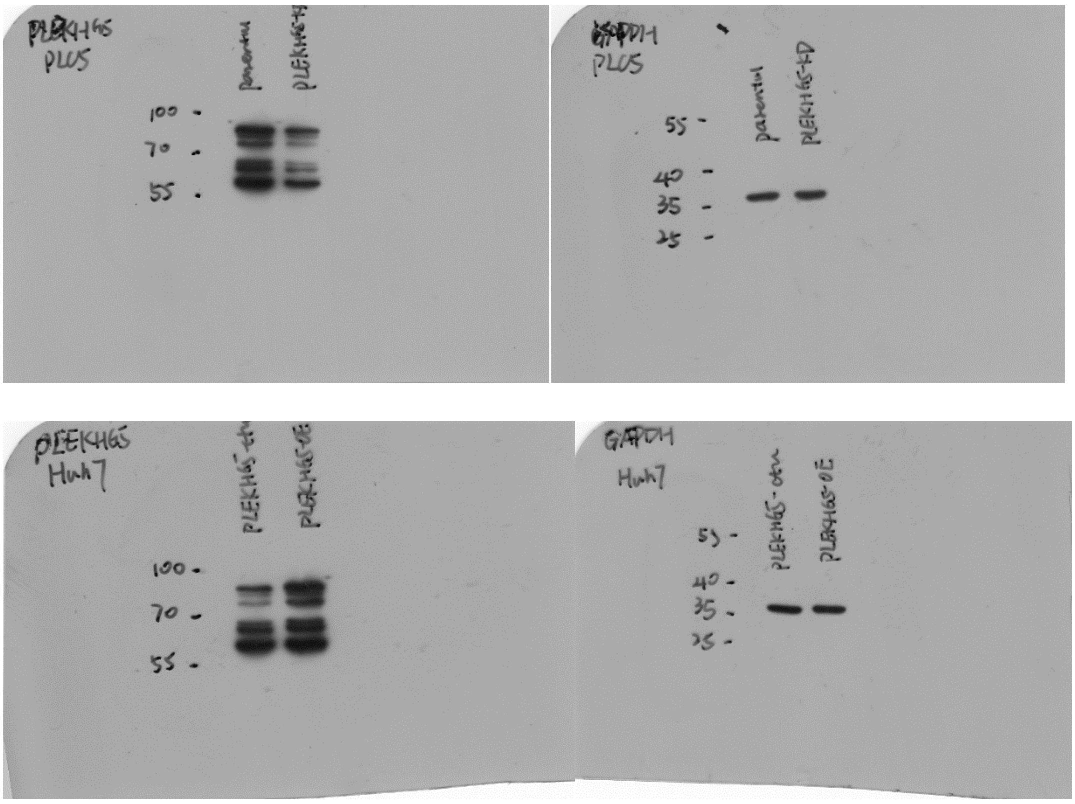

PLEKHG5
100
70
55
GAPDH
55
40
35
25
PLEKHG5
Huh7
100
70
55
GAPDH
Huh7
40
35

Figure 3C PLC/PRF/5

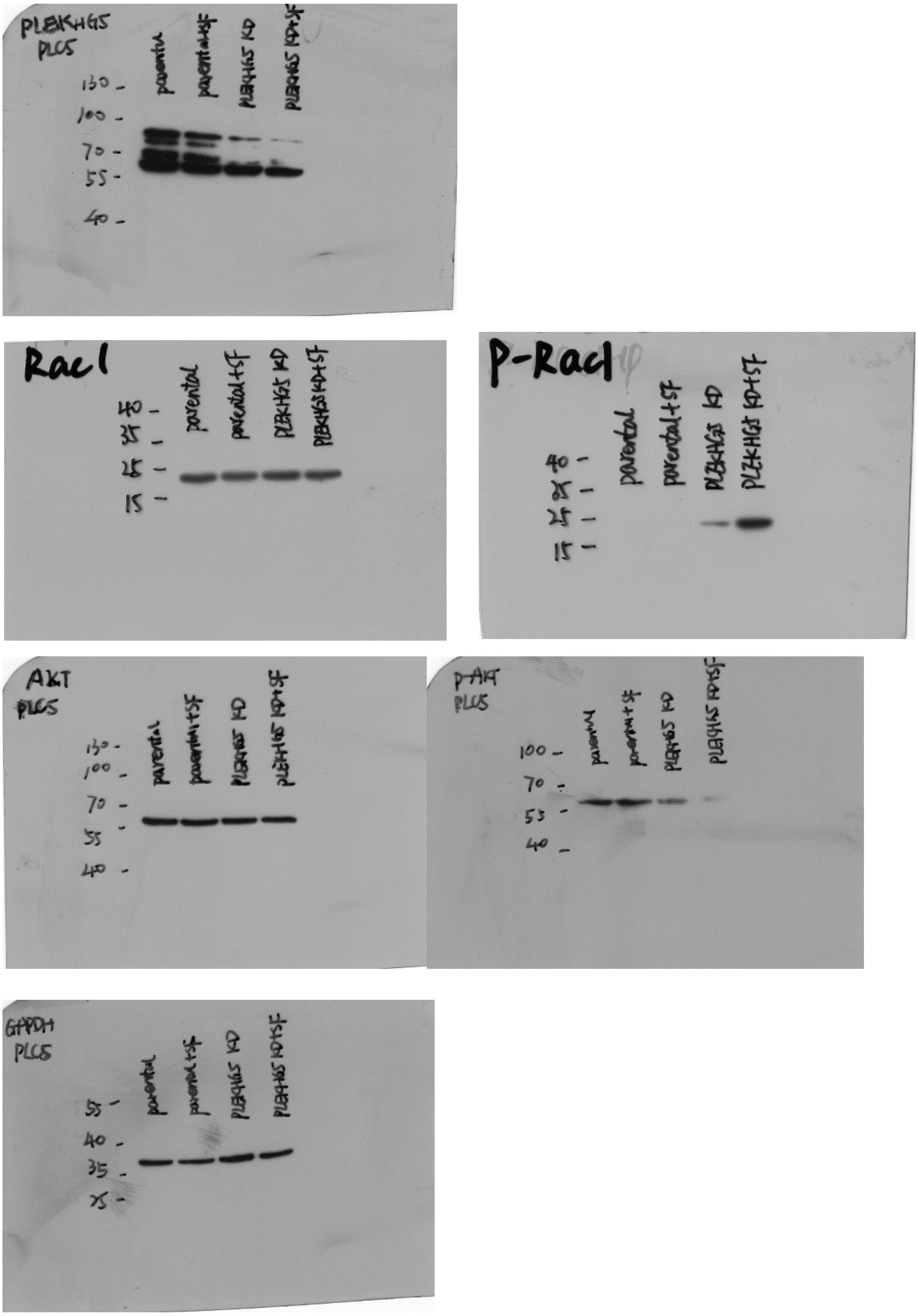

Figure 3C: Huh7

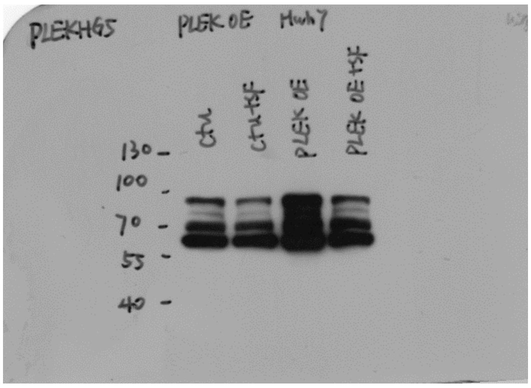

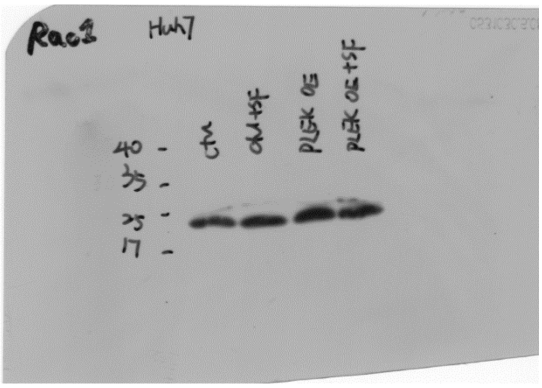

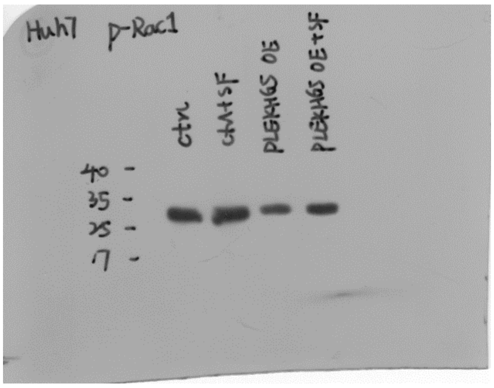

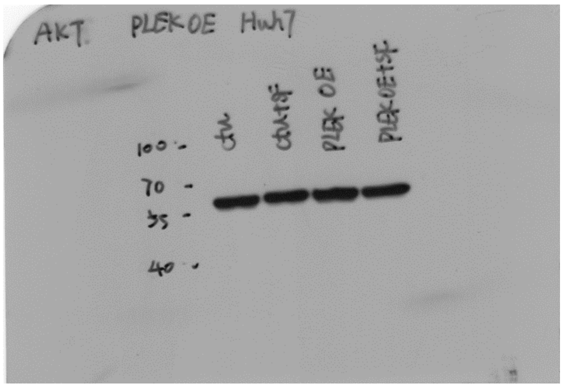

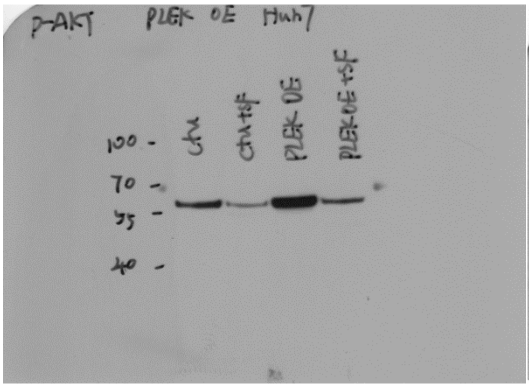

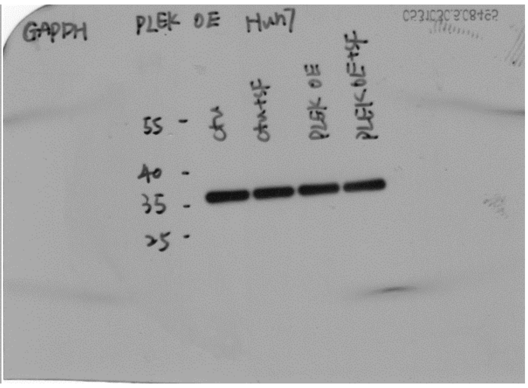

Figure 3I MHCC97H

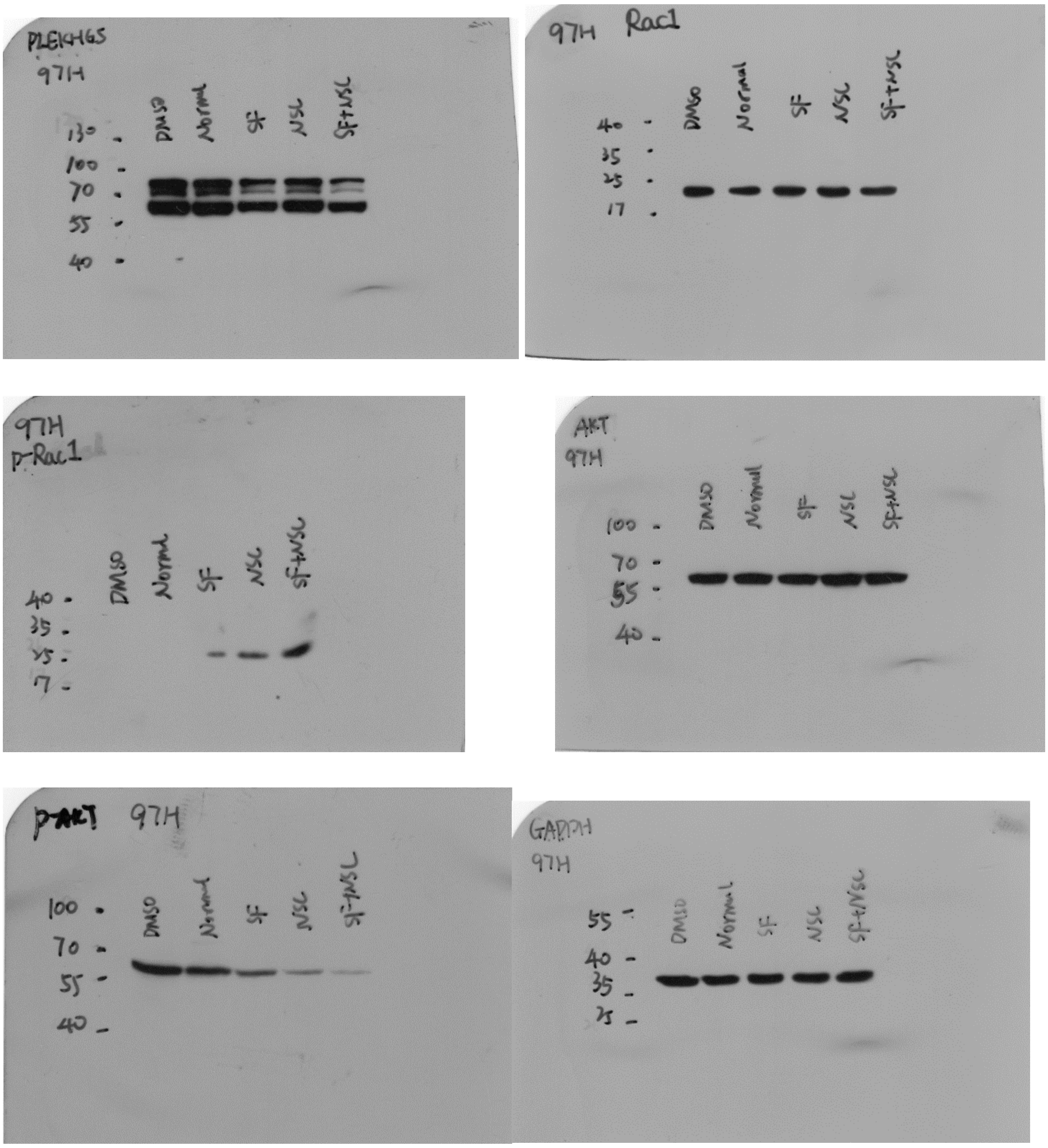

Figure 3I MHCC97H SR

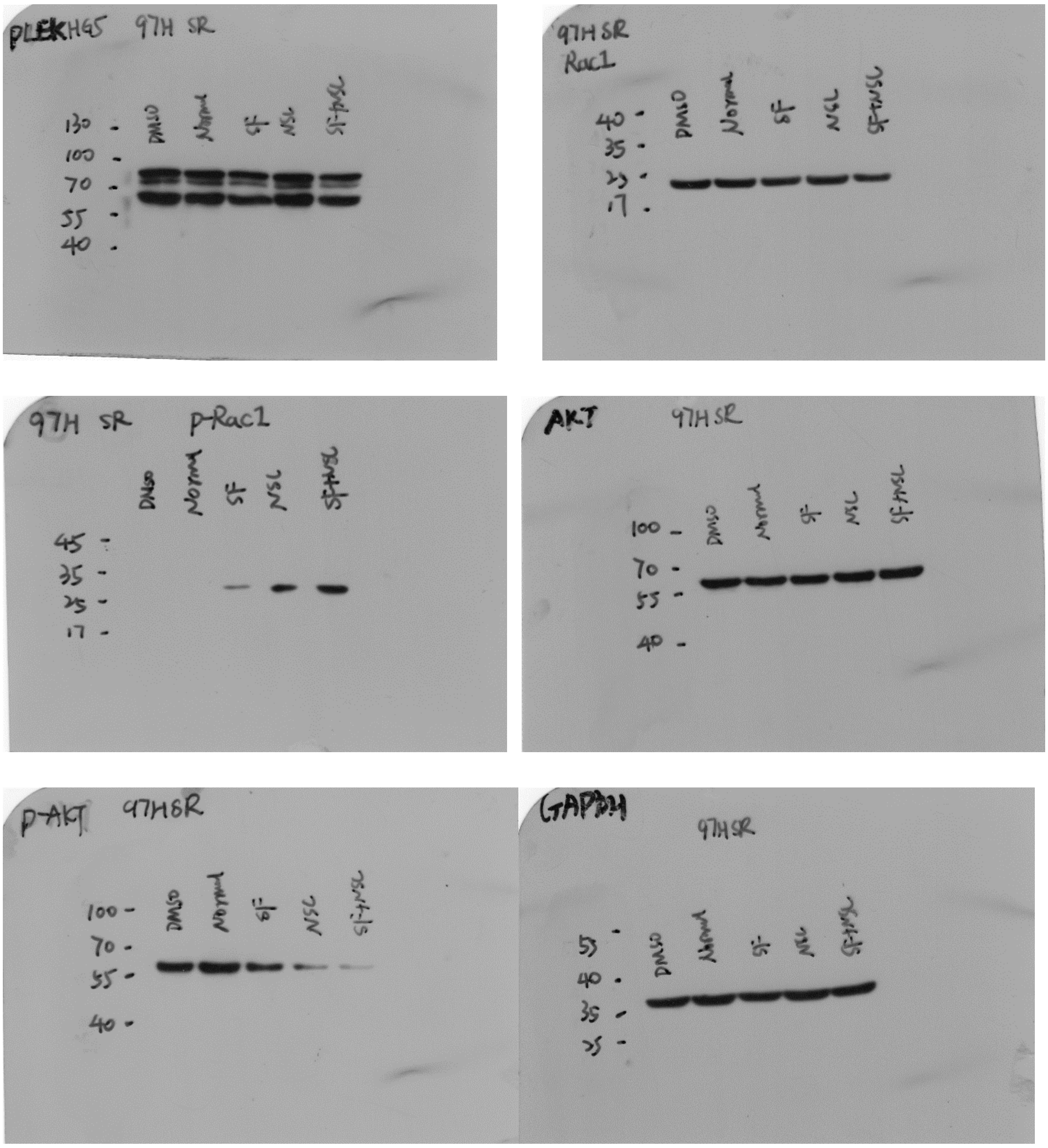

Figure 3J PLC/PRF/5

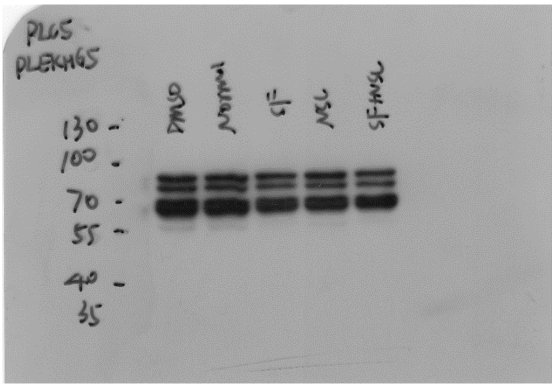

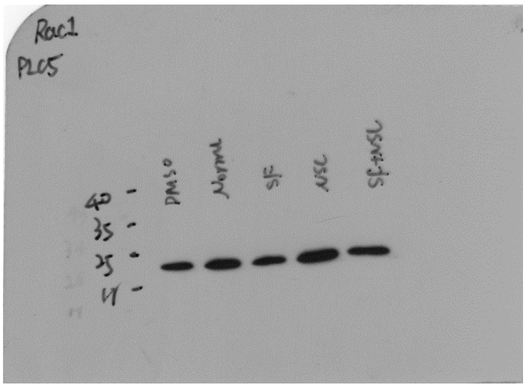

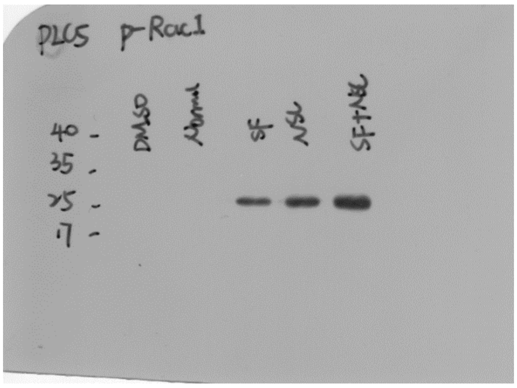

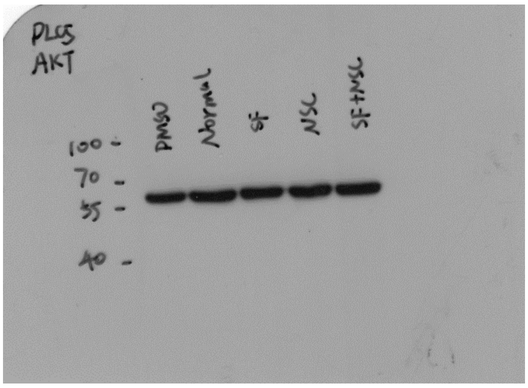

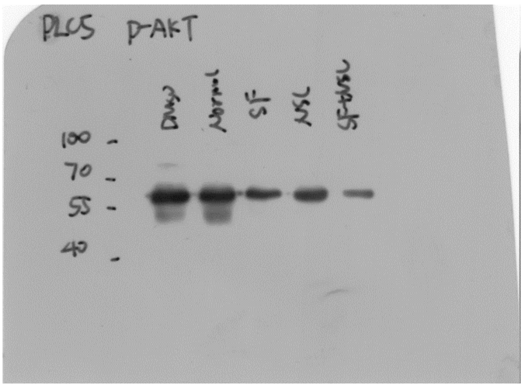

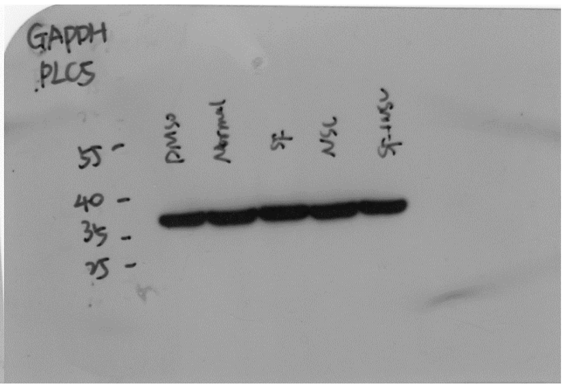

Figure 3J PLC/PRF/5 SR

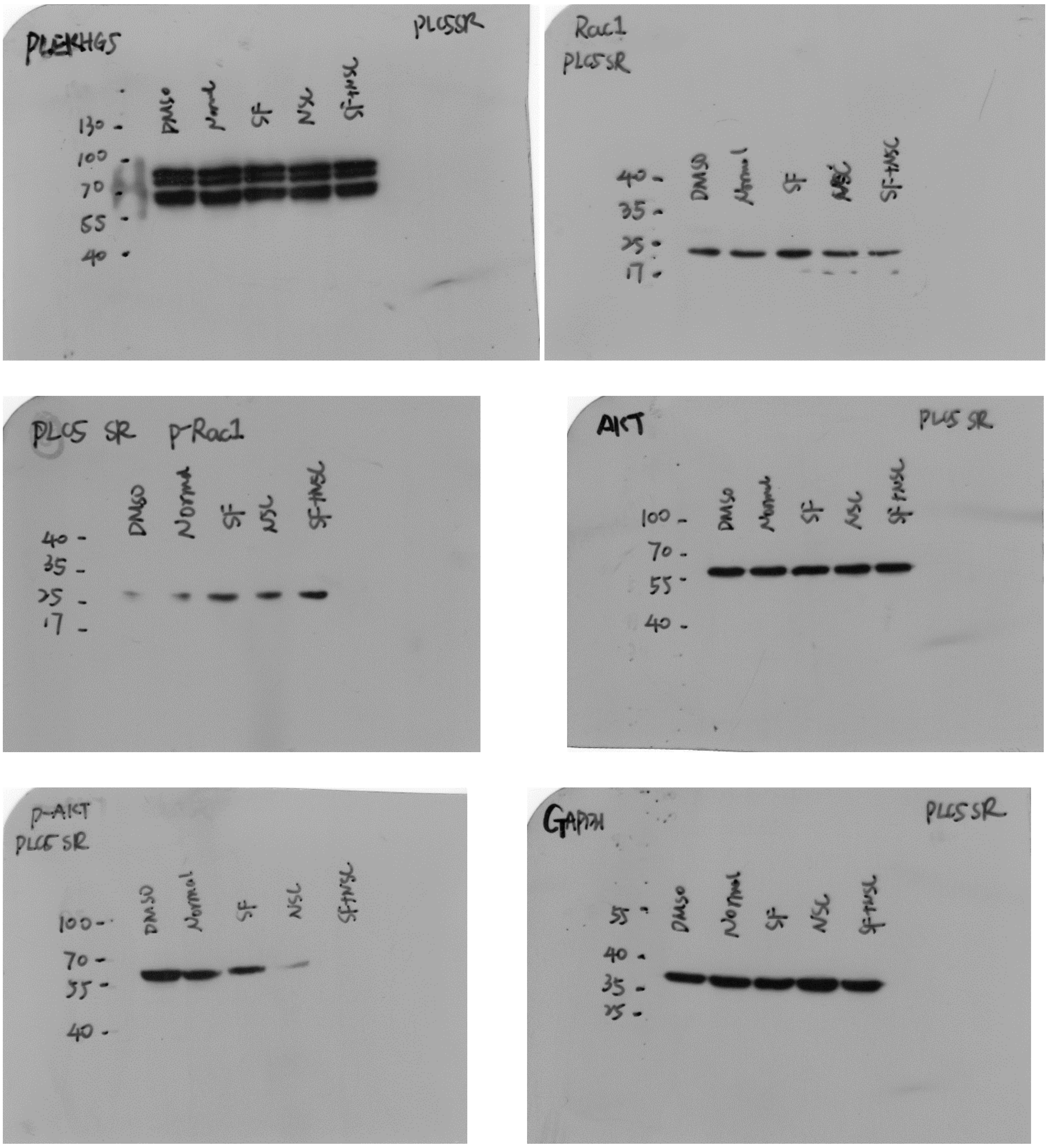

Figure 4A

IP-Flag

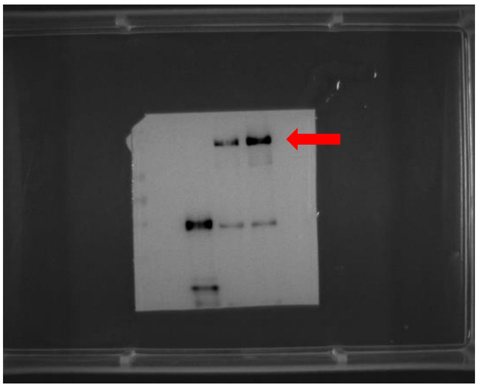

IP-ACE

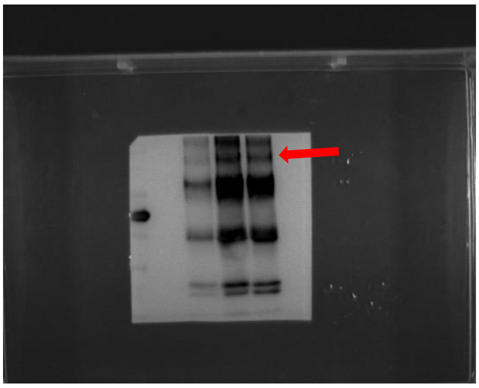

Input-Flag

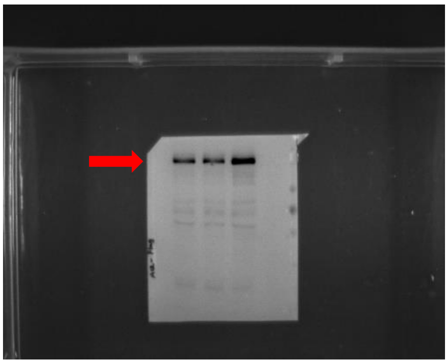

Input-ACE

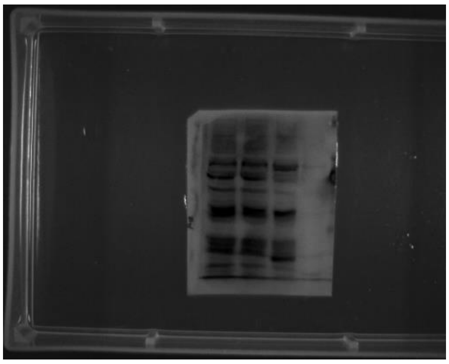

Input-GAPDH

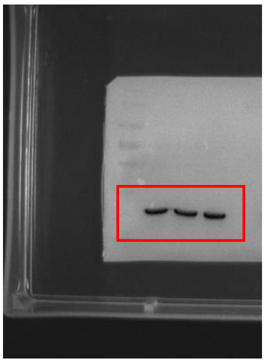

Figure 4C MHCC97H+PLC/PRF/5

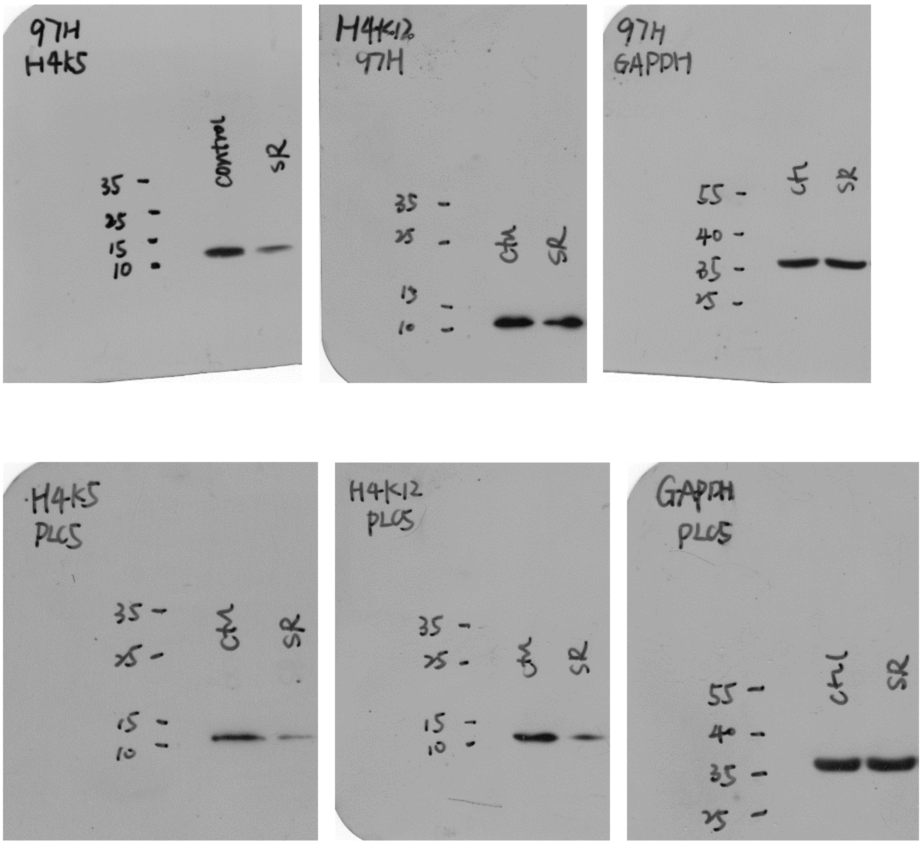

Figure 4E

CO-IP Flag (Input+IP)

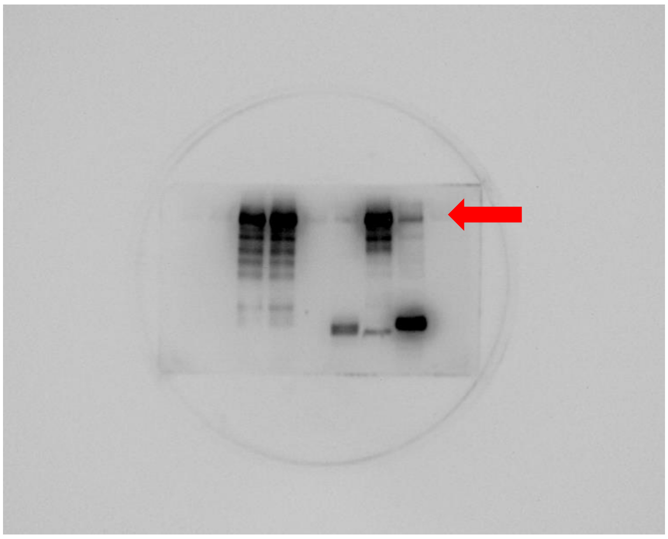

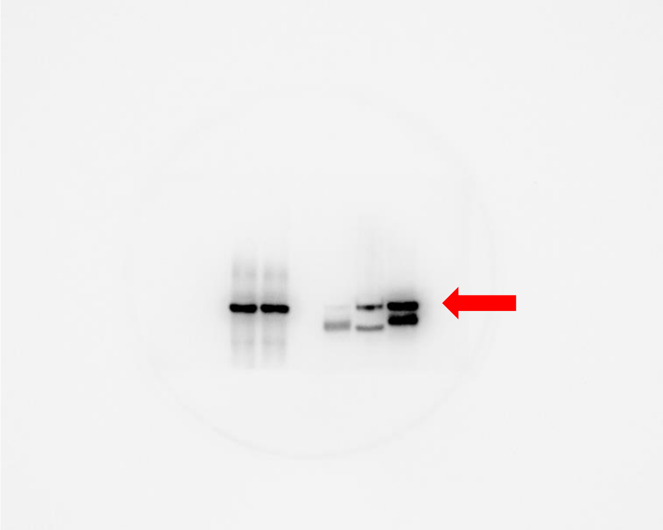

Figure 4F

Input-HDAC2

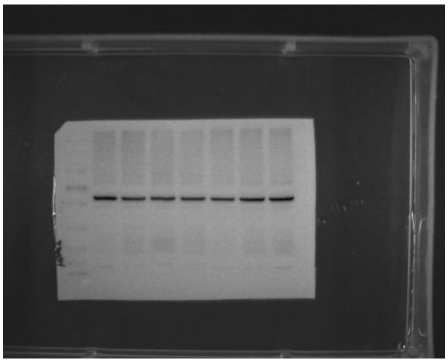

Input-Flag

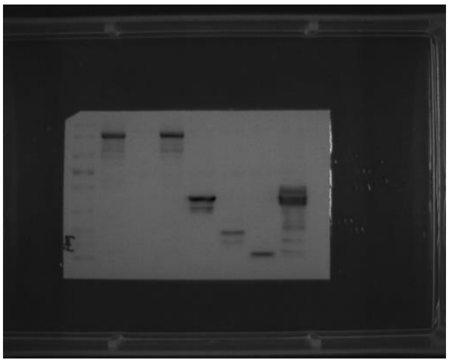

HDAC2 -IP-HDAC2

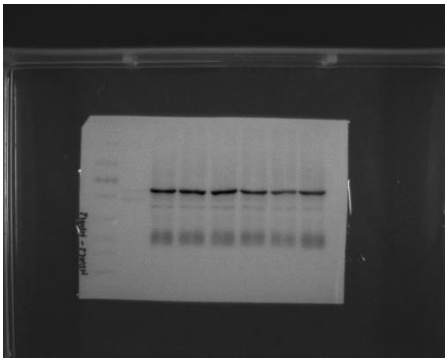

HDAC2-IP-Flag

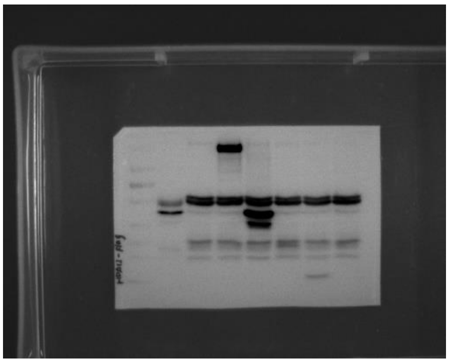

Flag-IP-Flag

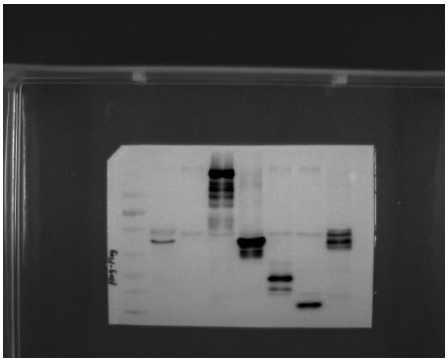

Flag-IP-HDAC2

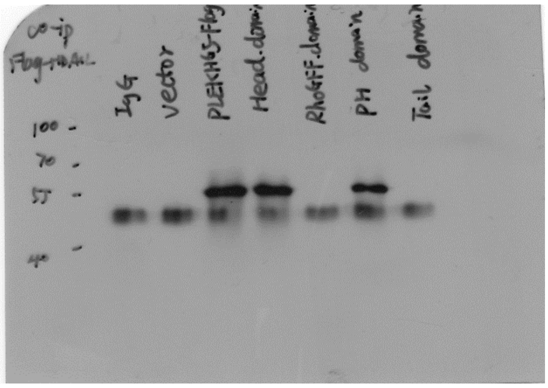

Figure 5A

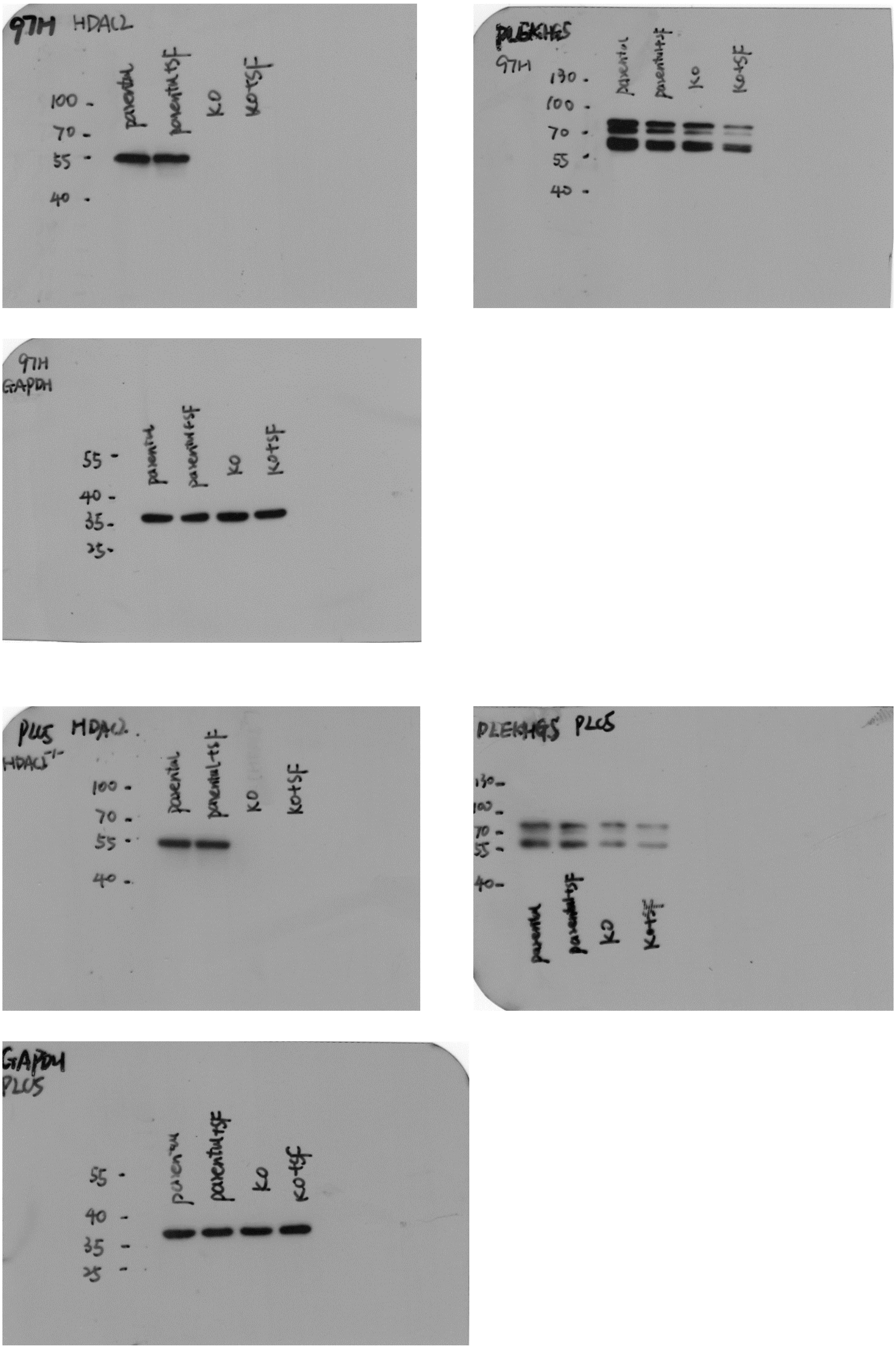

97H HDAC2
100
70
55
40
parental
parental+SF
KO
KO+SF
PLEKHG5
97H
130
100
70
55
40
parental
parental+SF
KO
KO+SF
97H
GAPDH
55
40
35
25
parental
parental+SF
KO
KO+SF
PLC5 HDAC2
100
70
55
40
parental
parental+SF
KO
KO+SF
PLEKHG5 PLC5
130
100
70
55
40
parental
parental+SF
KO
KO+SF
GAPDH
PLC5
55
40
35
25
parental
parental+SF
KO
KO+SF

Figure 5C

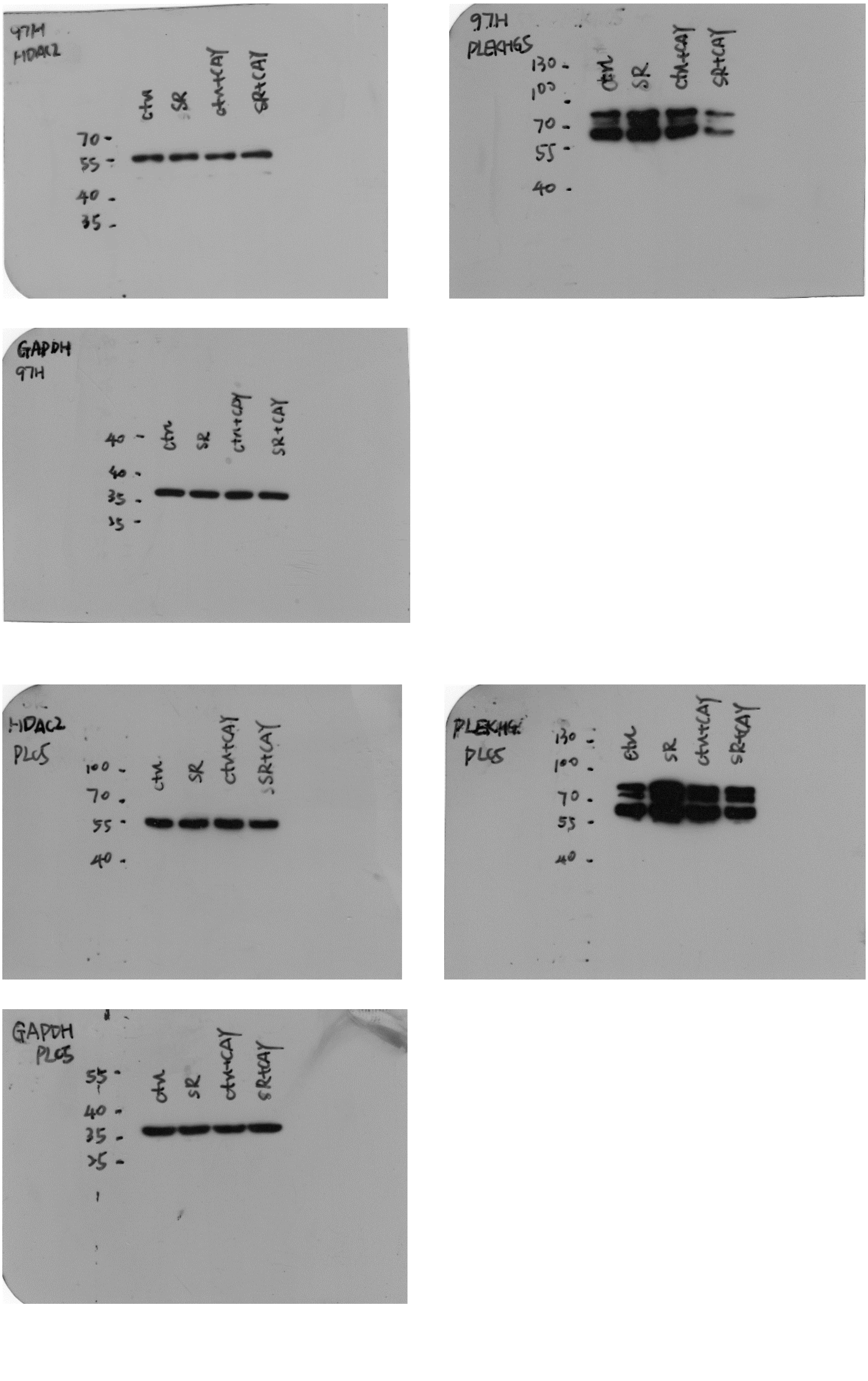
97H
HDAC2
PLEKHG5
GAPDH
PLC5
ctrl
SR
ctrl+CAY
SR+CAY
130
100
70
55
40
35

Figure 5E MHCC97H

Input-Flag

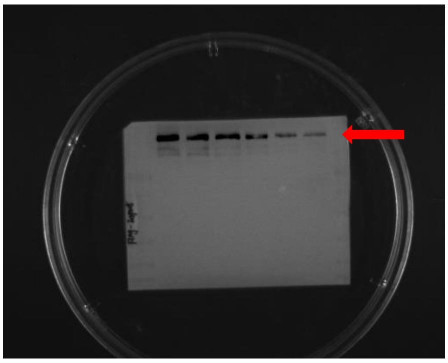

Input-Ace

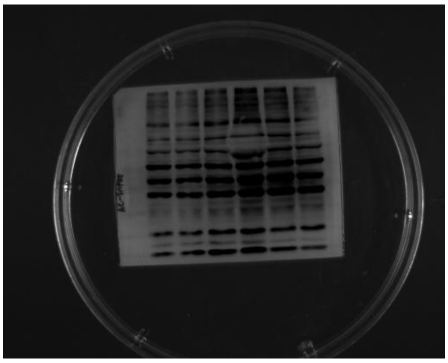

Input-GAPDH

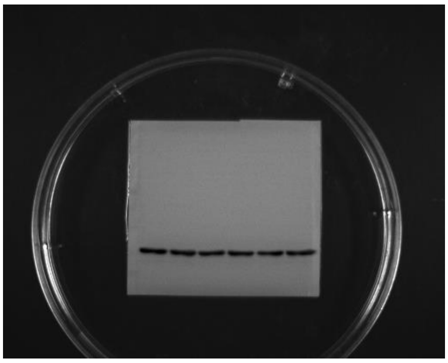

IP-Flag

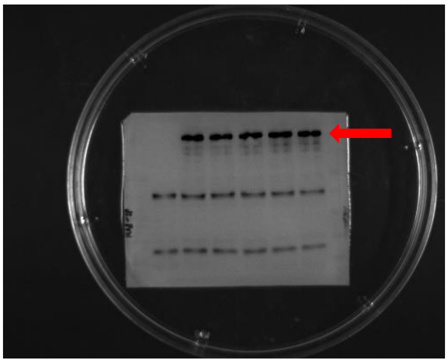

IP-ACE

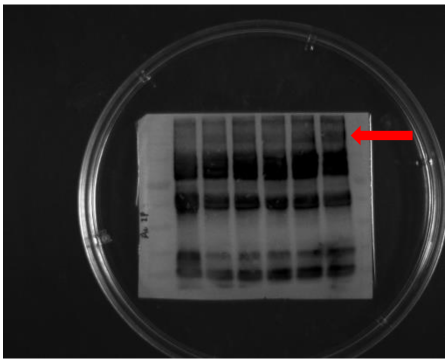

Figure 5E PLC/PRF/5

Input Flag

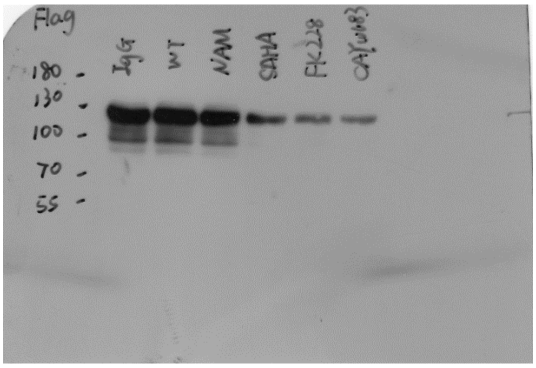

Input-Ace

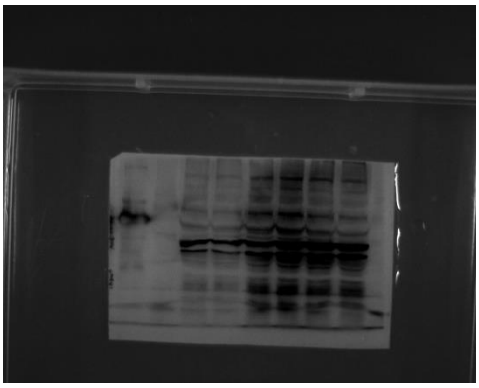

Input GAPDH

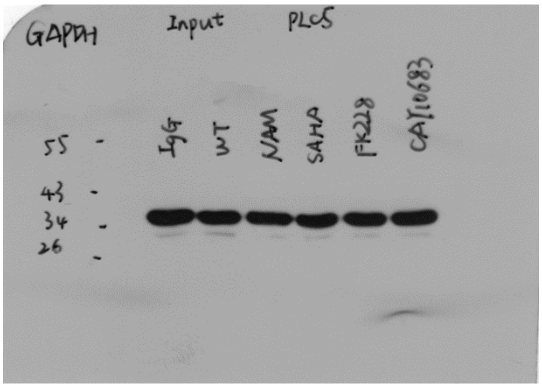

IP-Flag

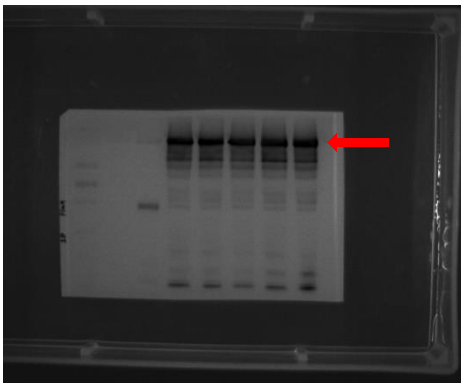

IP-Ace

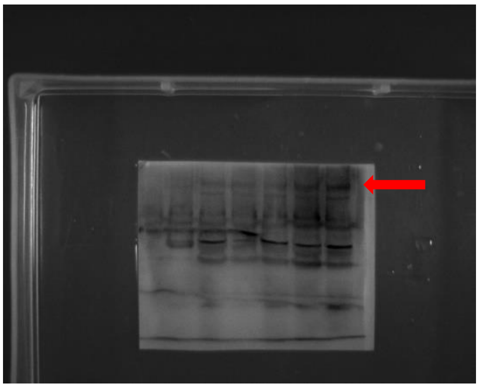

Figure 5F

MHCC97H

Input -Ace

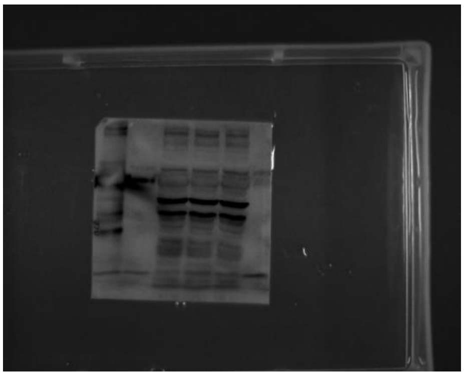

Input+IP   Flag

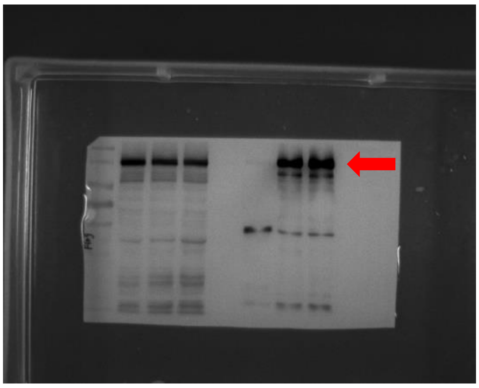

IP Ace

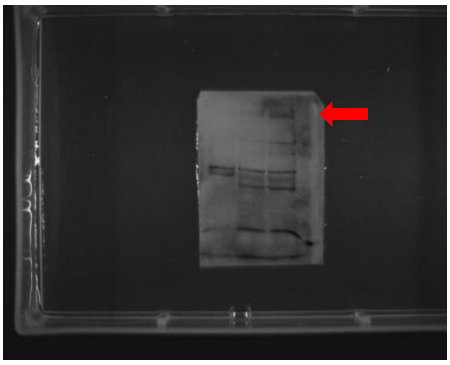

PLC/PRF/5

Input+IP-Flag

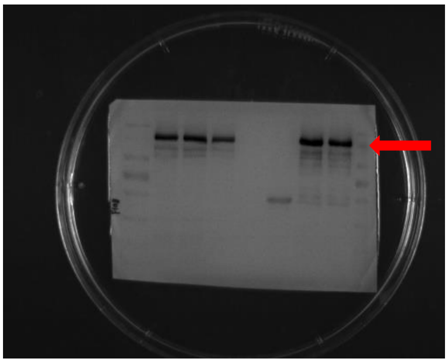

Input-Ace

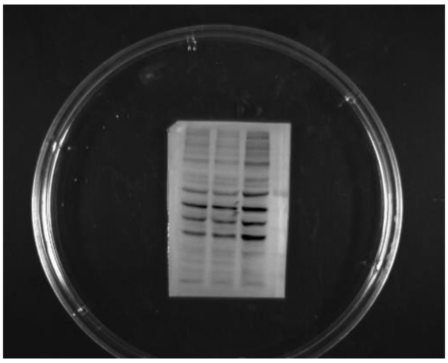

IP-Ace

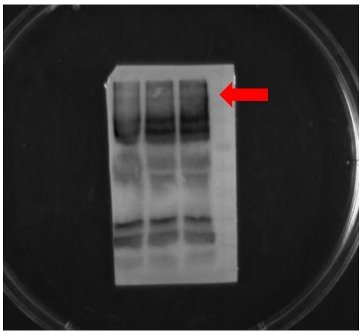

Figure 5G MHCC97H

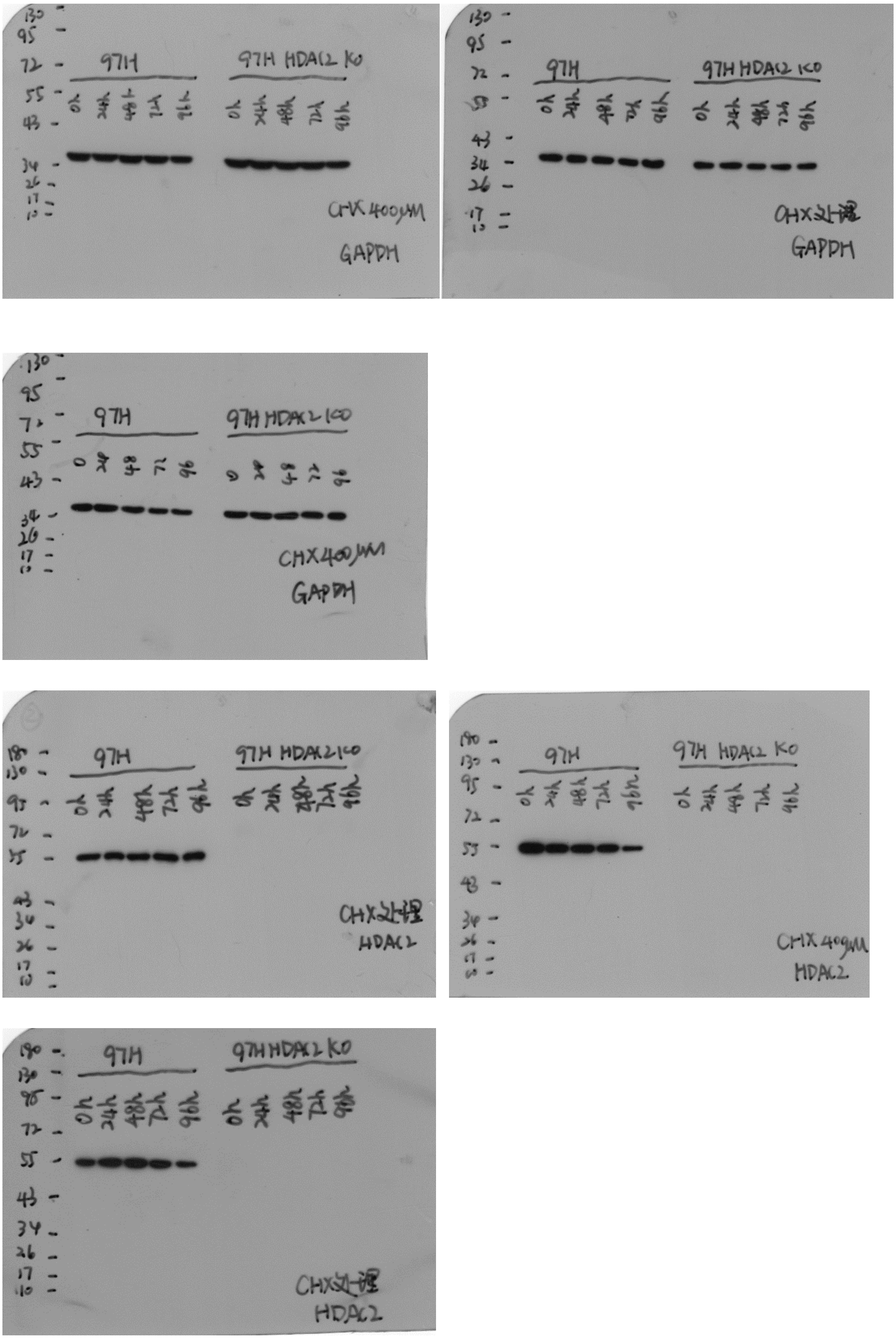

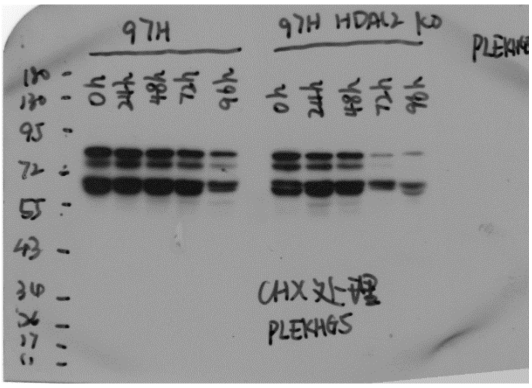
97H
97H HDAC2 KO
CHX处理
PLEKHG5

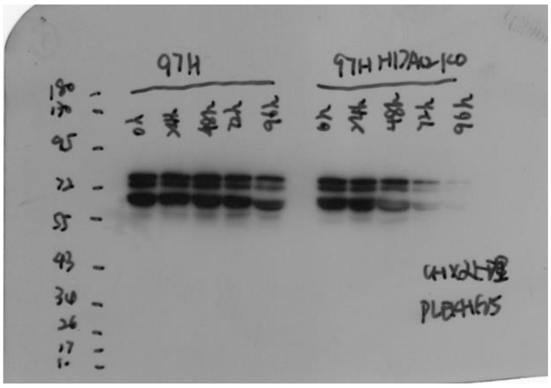
97H
97H HDAC2 KO
CHX处理
PLEKHG5

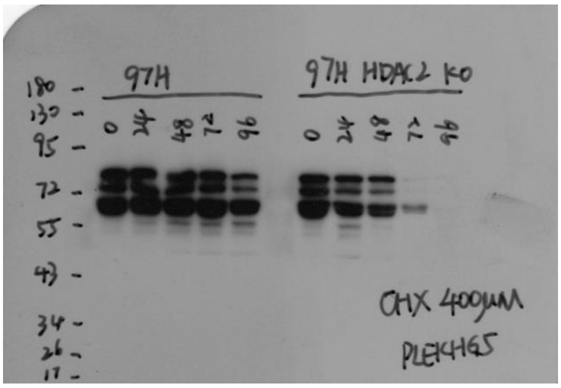
97H
97H HDAC2 KO
CHX 400μM
PLEKHG5

Figure 5G PLC/PRF/5

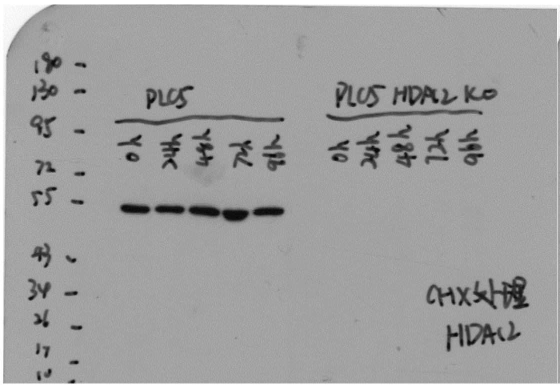

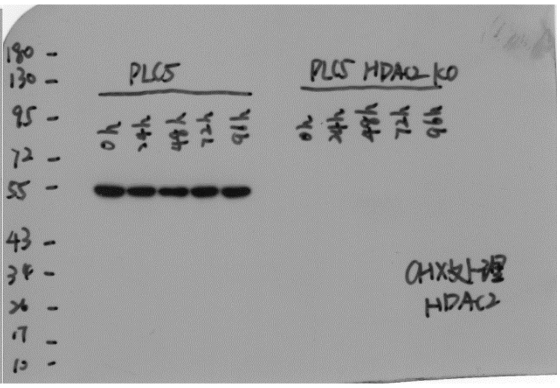

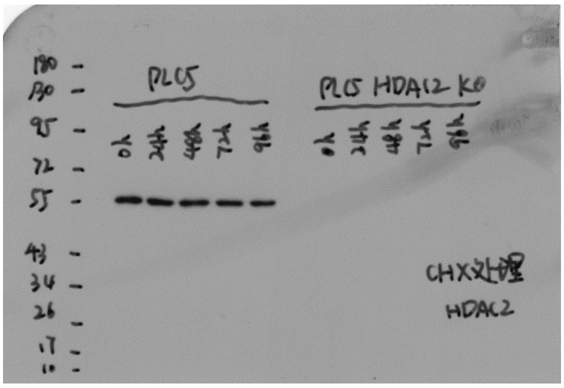

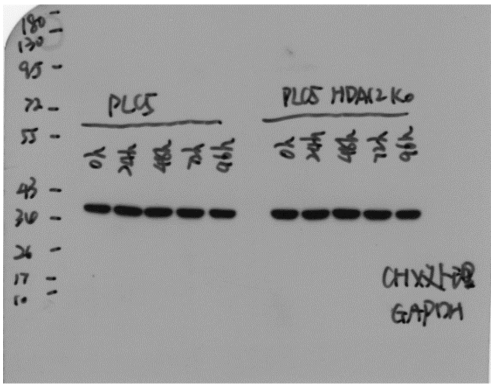

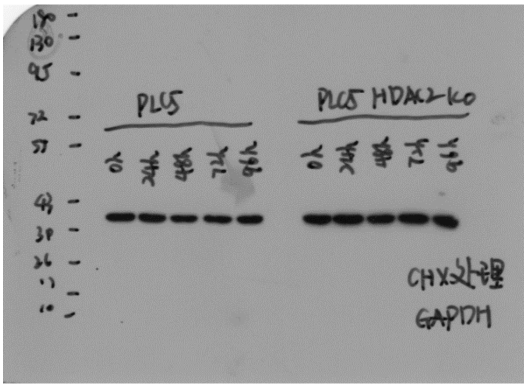

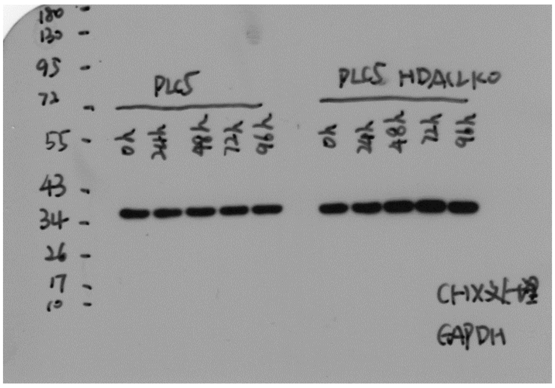

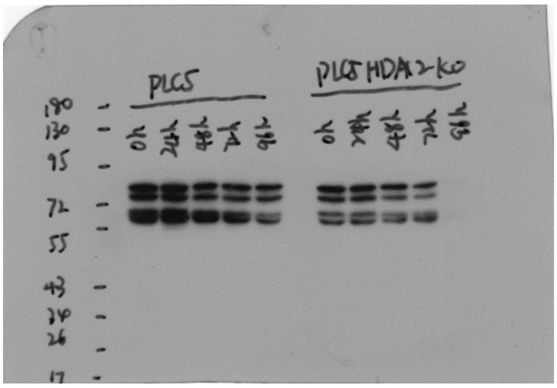
PLC5
PLC5 HDAC2 KO
180
130
95
72
55
43
34
26
17

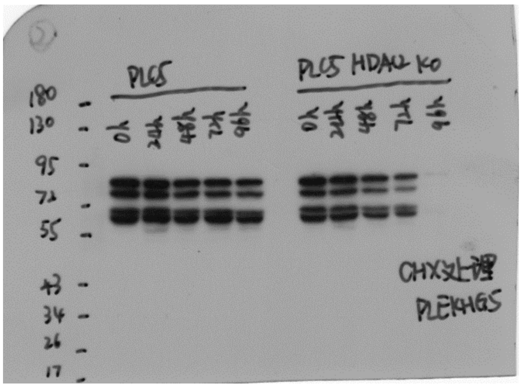
PLC5
PLC5 HDAC2 KO
180
130
95
72
55
43
34
26
17
CHX処理
PLEKHG5

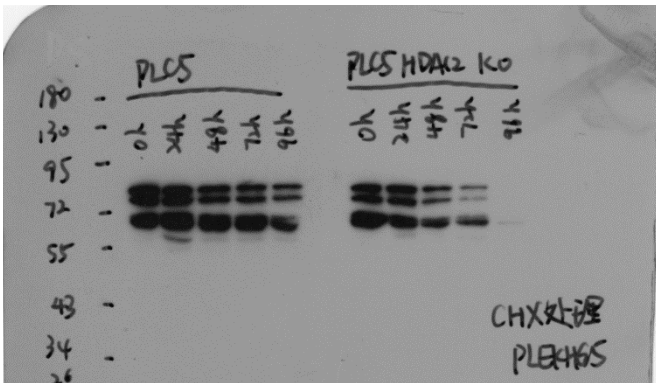
PLC5
PLC5 HDAC2 KO
180
130
95
72
55
43
34
CHX処理
PLEKHG5

Figure 6B MHCC97H

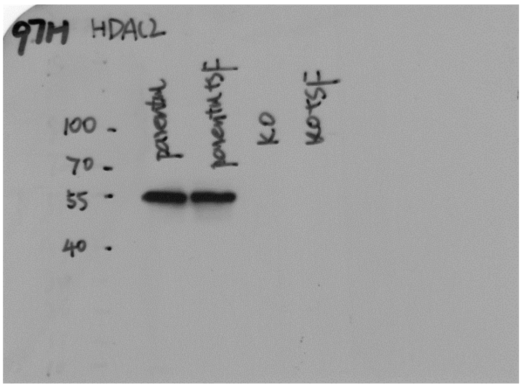

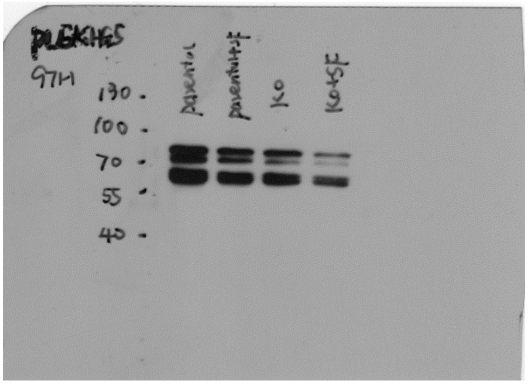

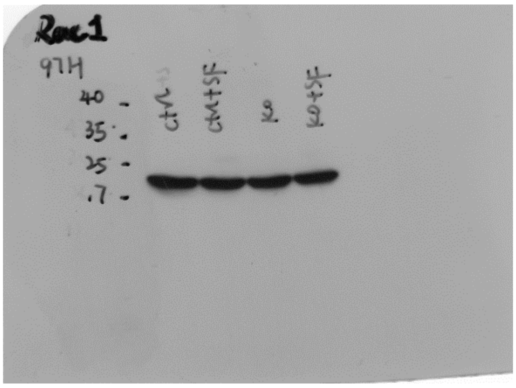

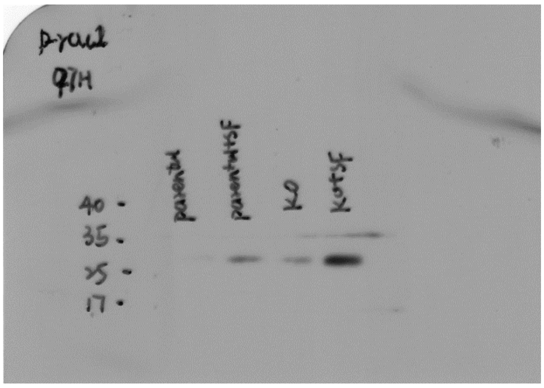

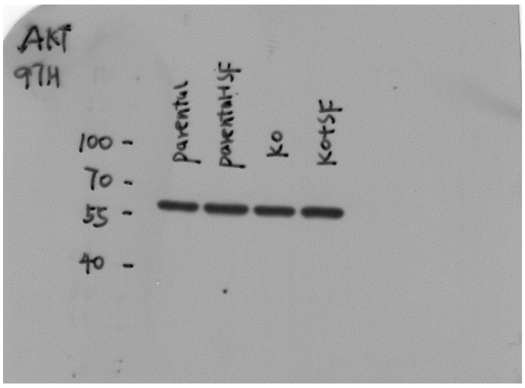

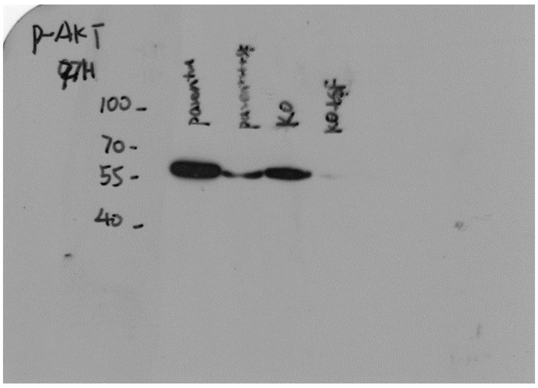

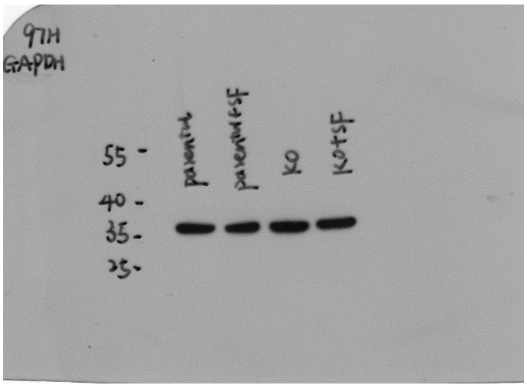

Figure 6B PLC/PRF/5

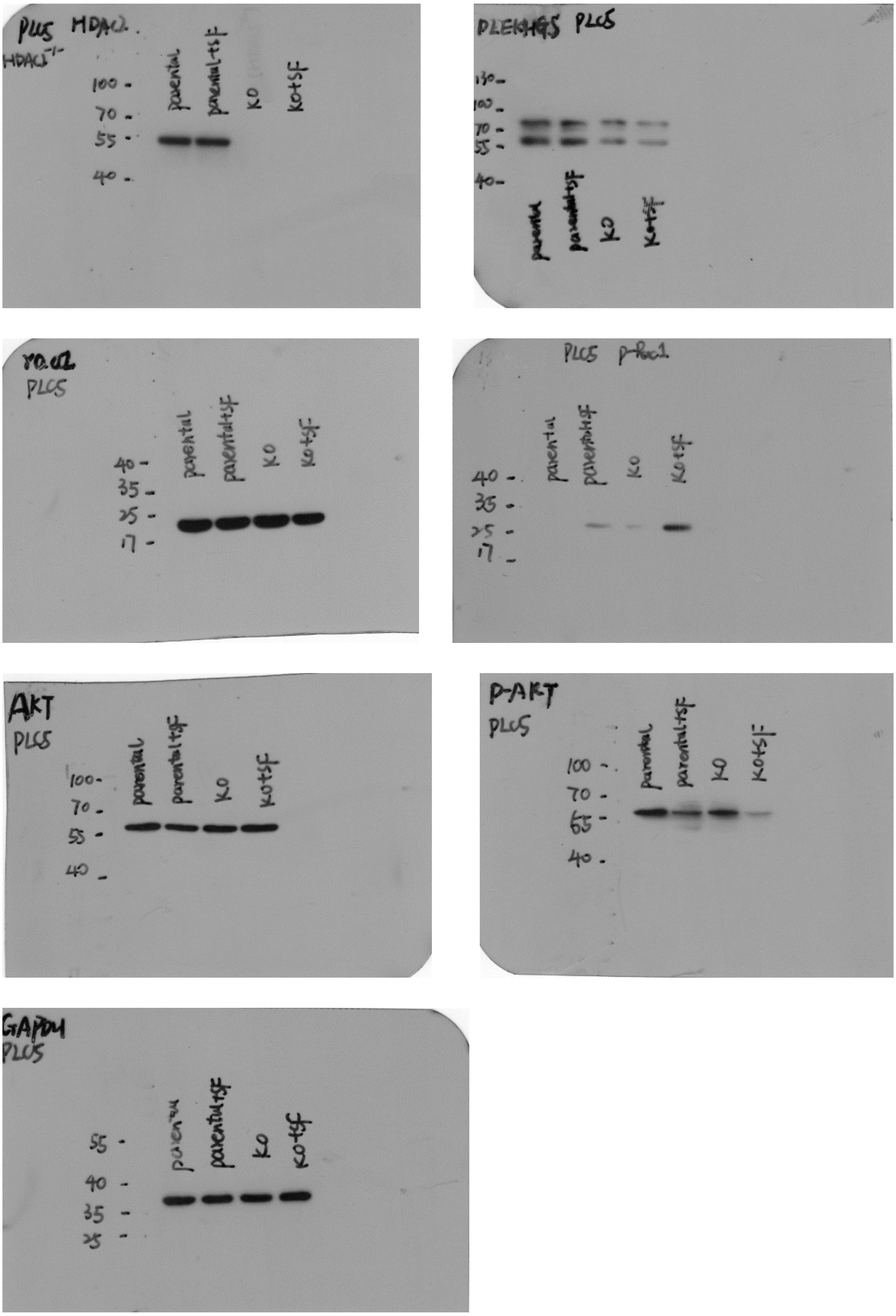

Figure 6E

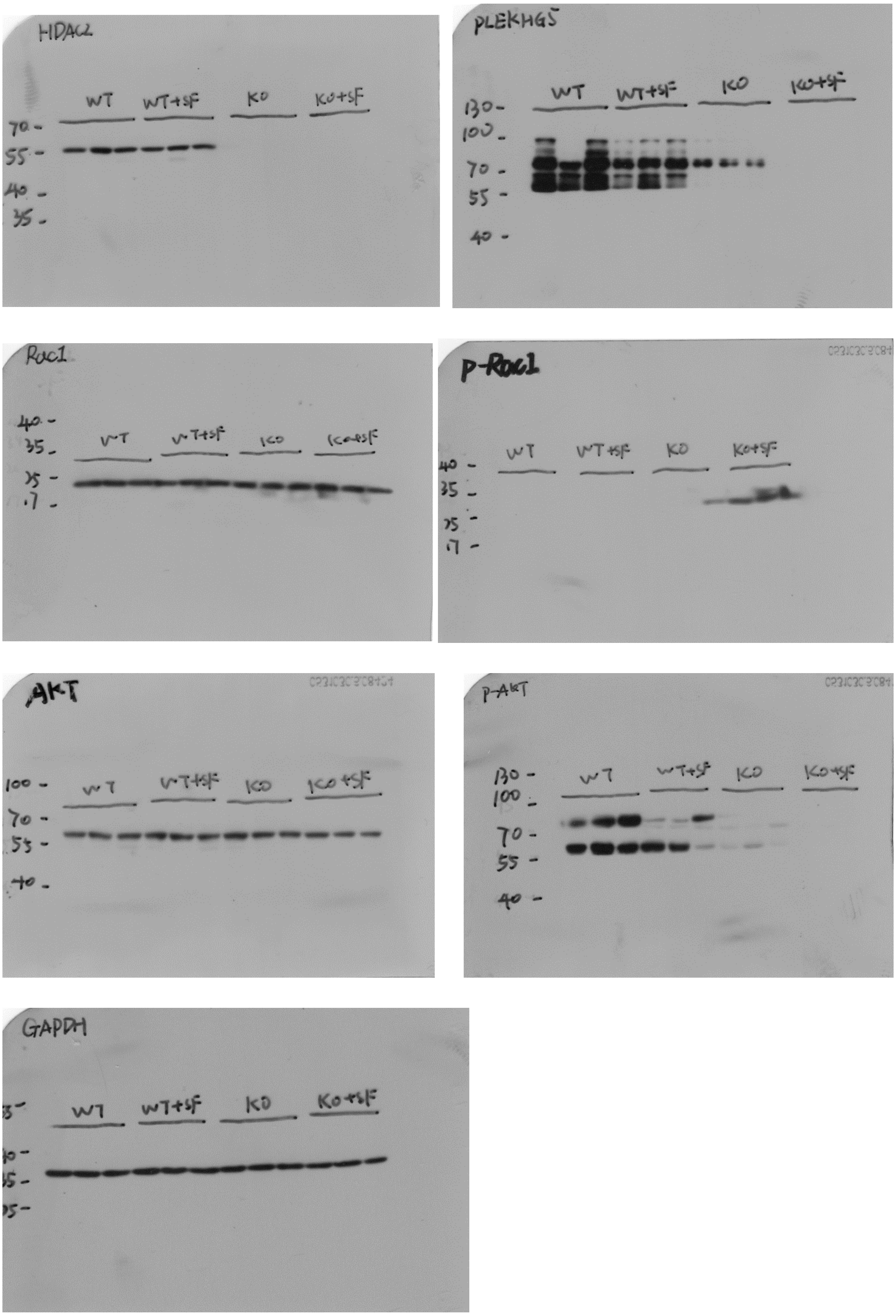

Figure 7C MHCC97H

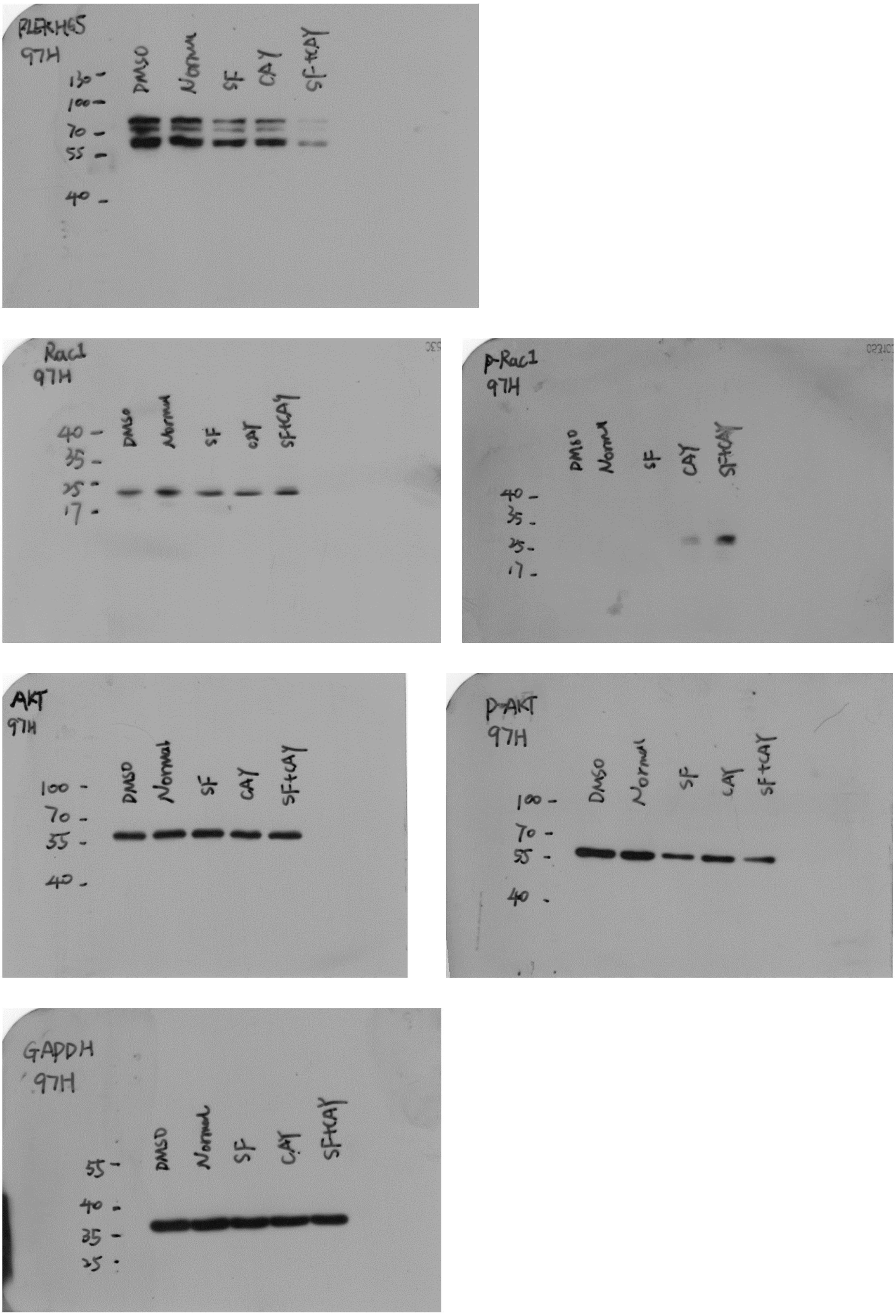

Figure 7C MHCC97H SR

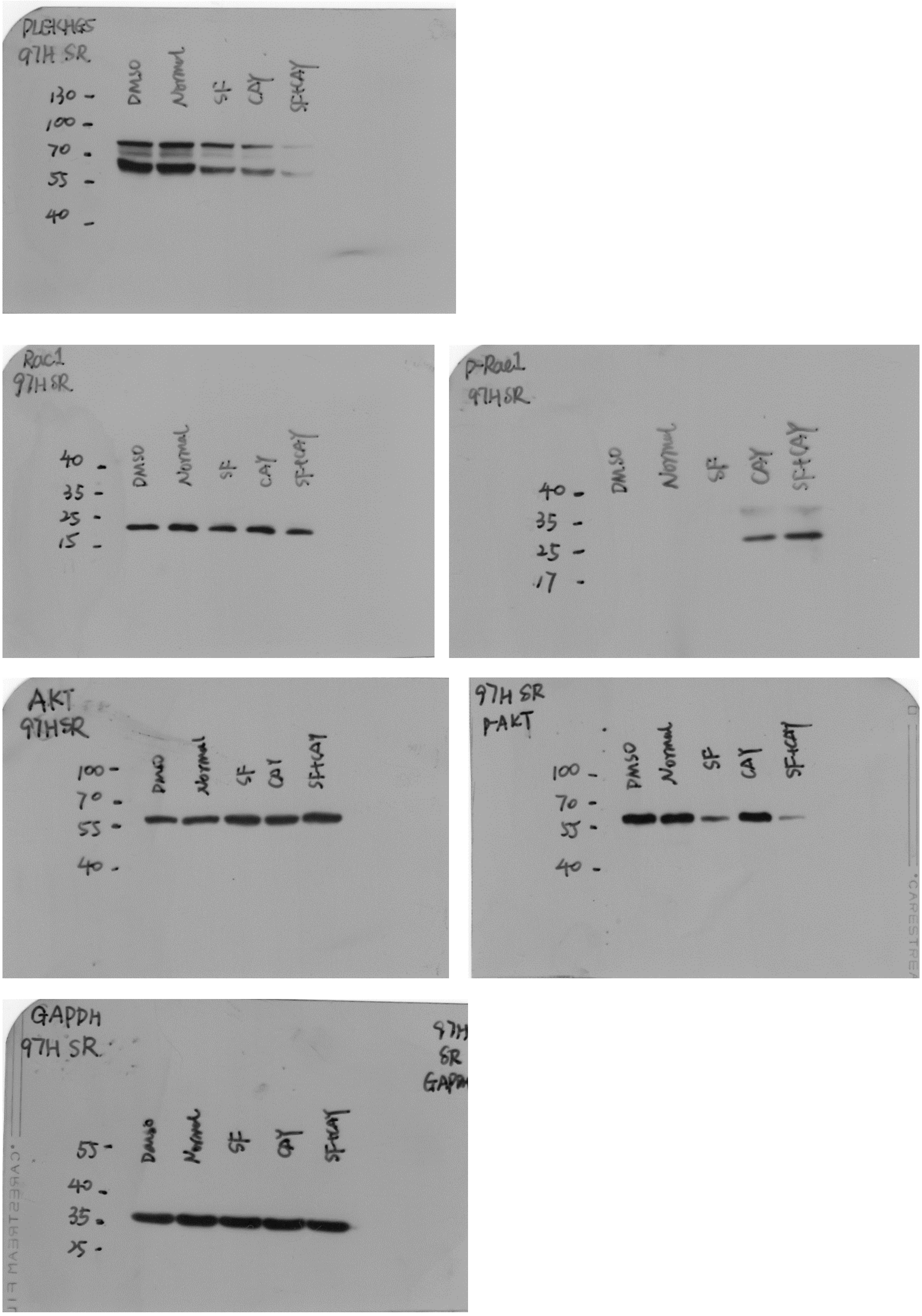

Figure 7D PLC/PRF/5

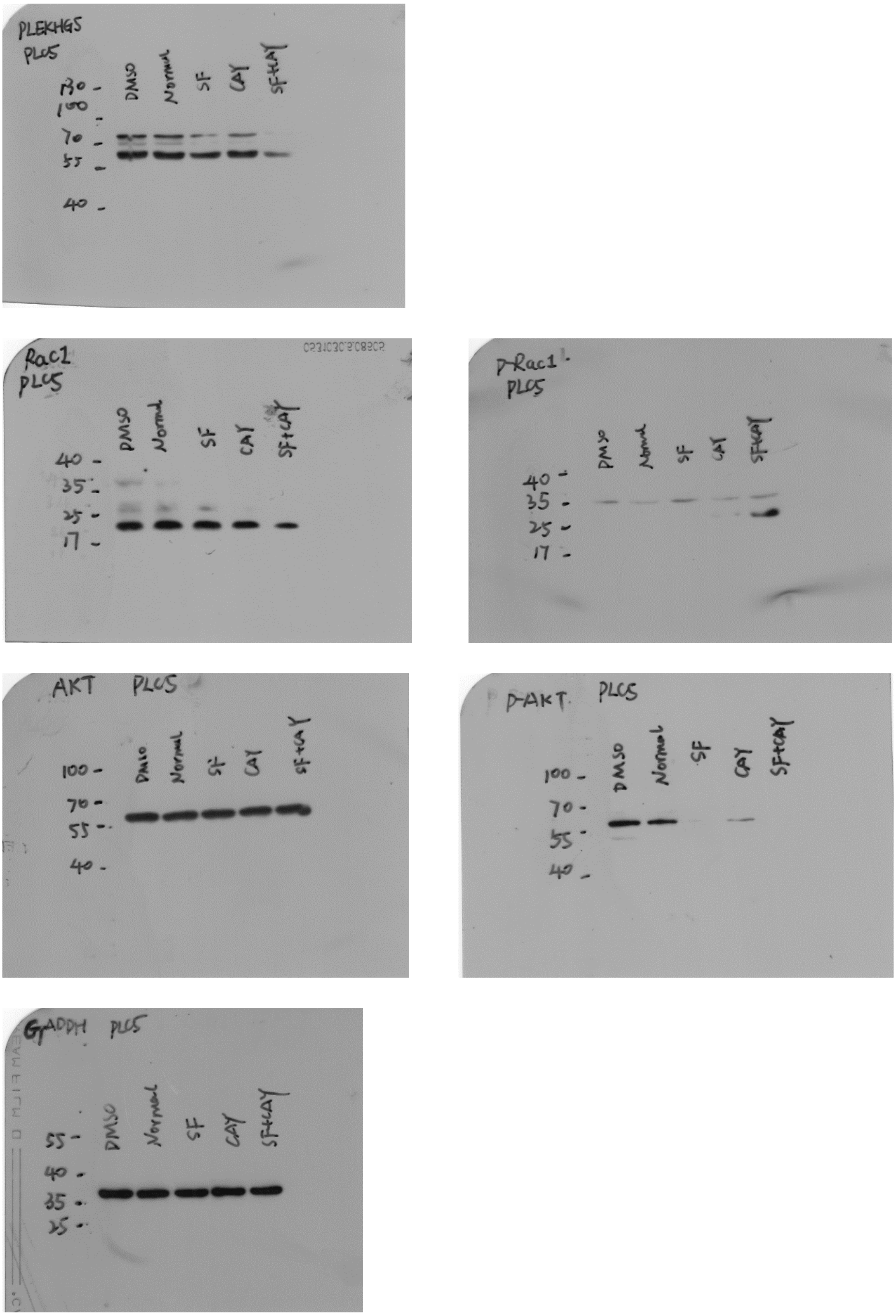

Figure 7D PLC/PRF/5 SR

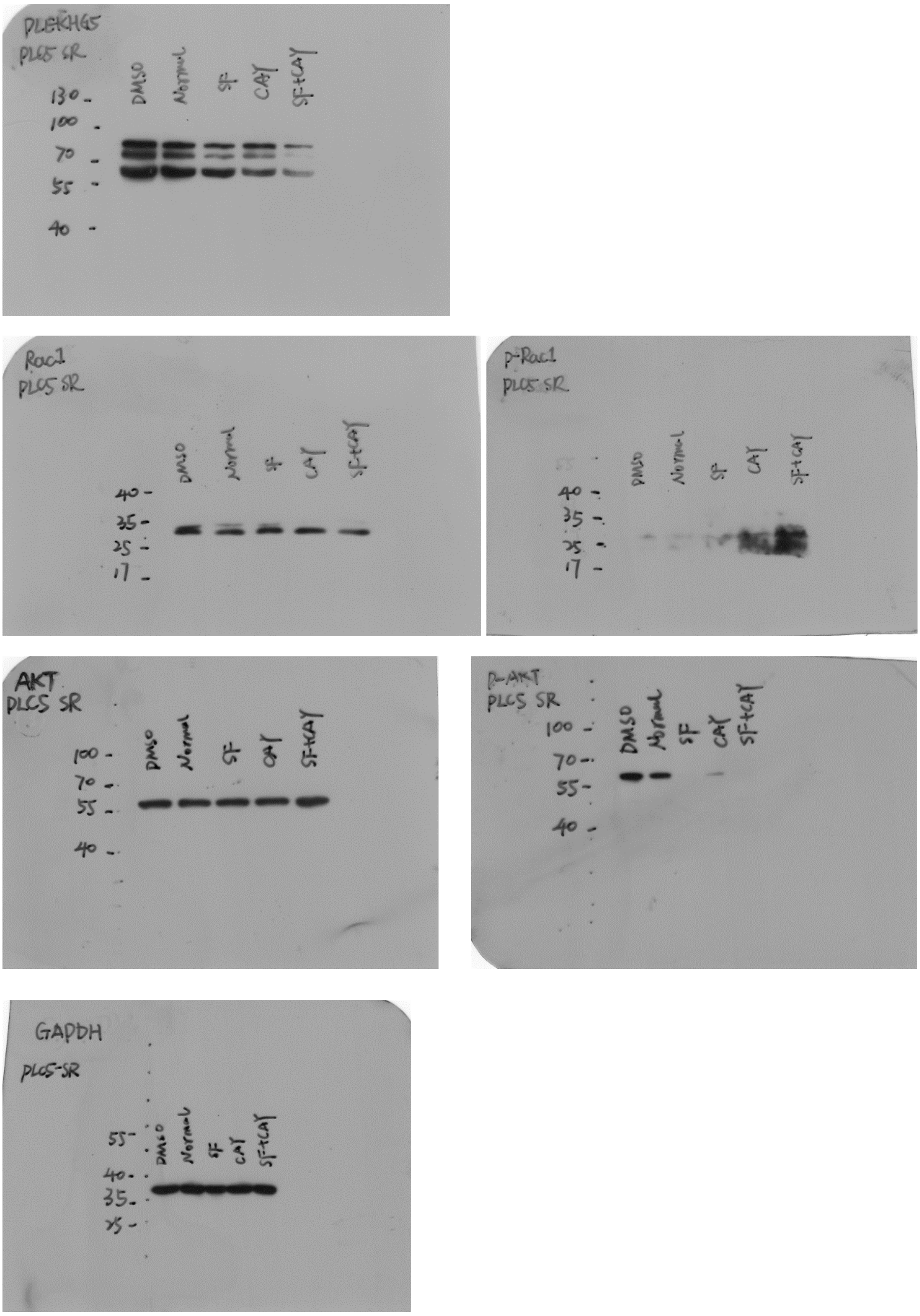

Figure 7H

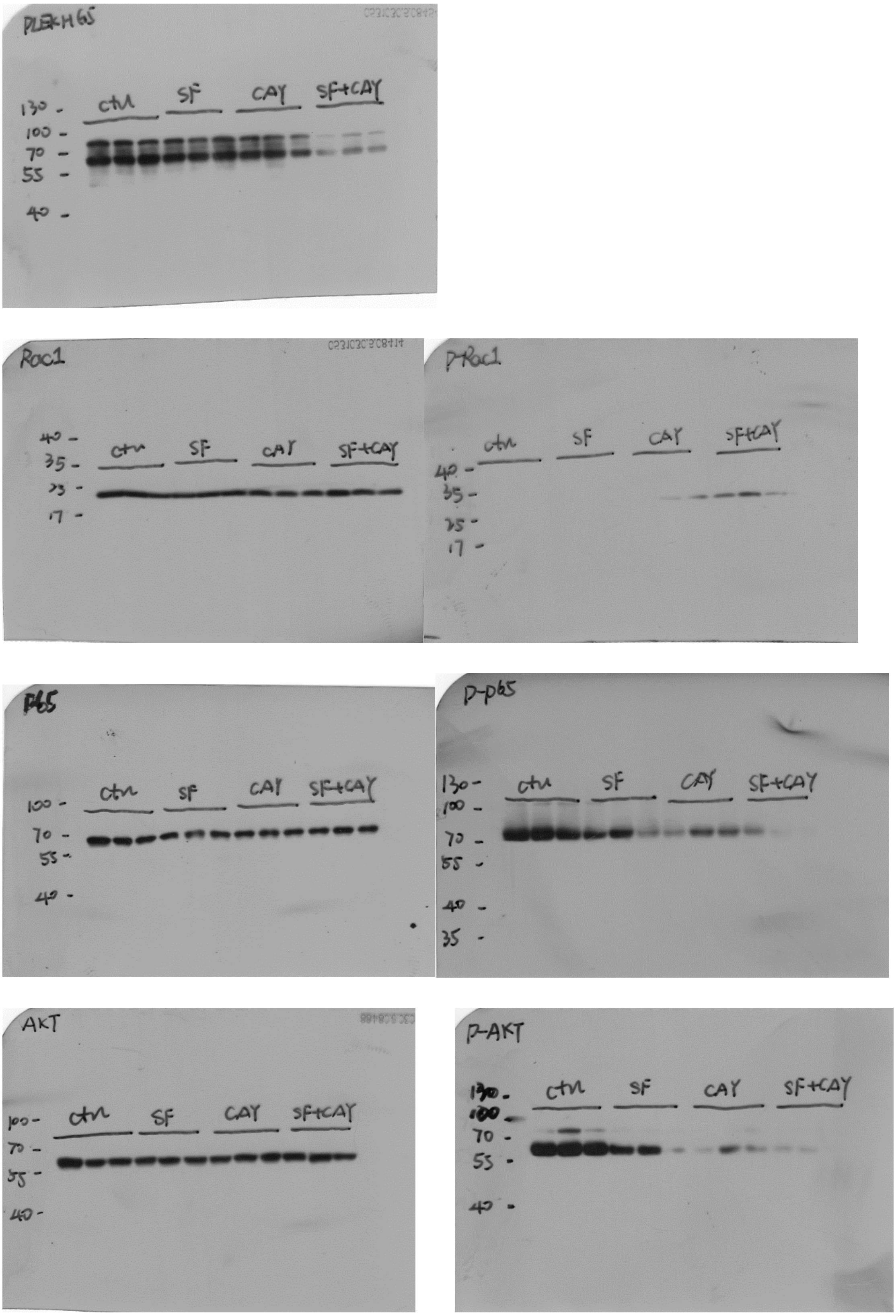

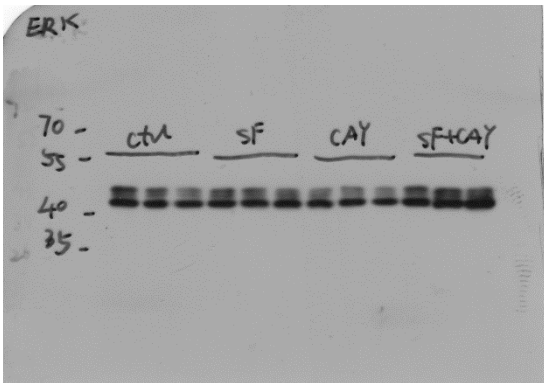
ERK
70
55
40
35
ctrl
SF
CAY
SF+CAY

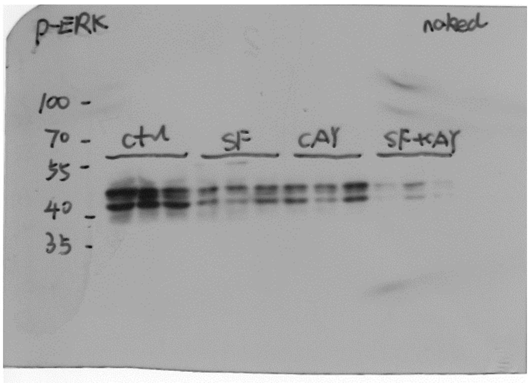
p-ERK
naked
100
70
55
40
35
ctrl
SF
CAY
SF+CAY

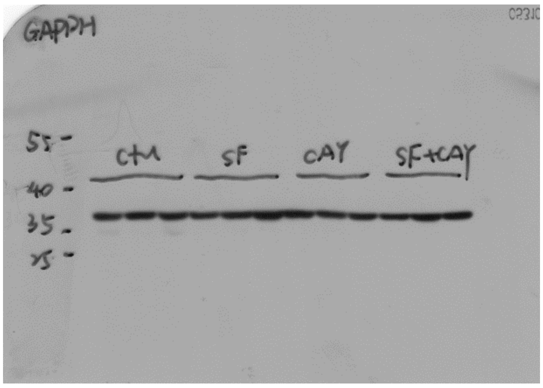
GAPDH
55
40
35
25
ctrl
SF
CAY
SF+CAY

Figure S1E MHCC97H

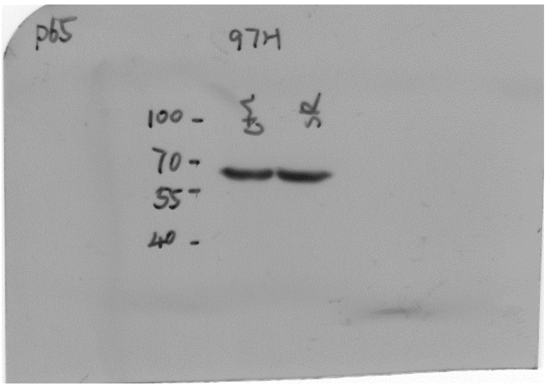

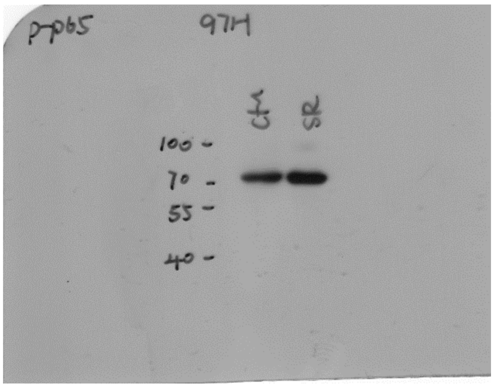

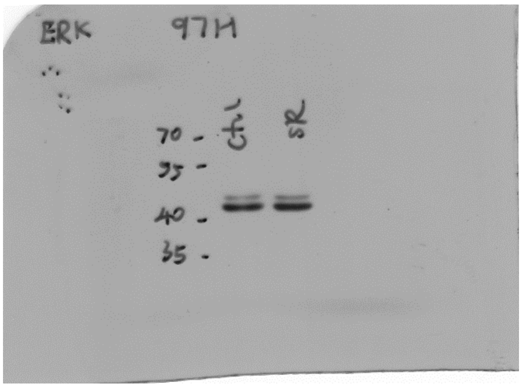

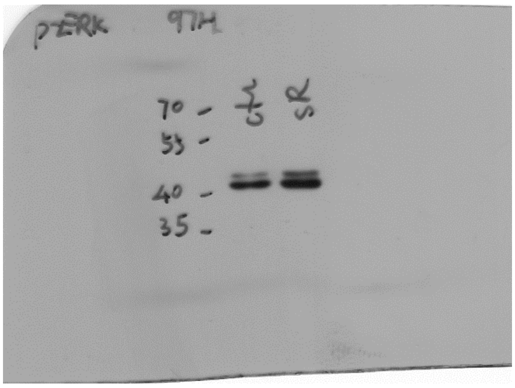

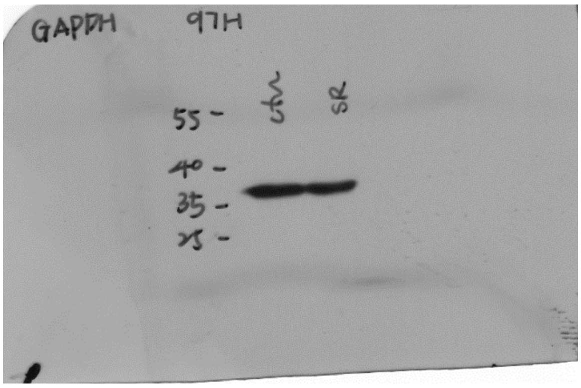

Figure S1D PLC/PRF/5

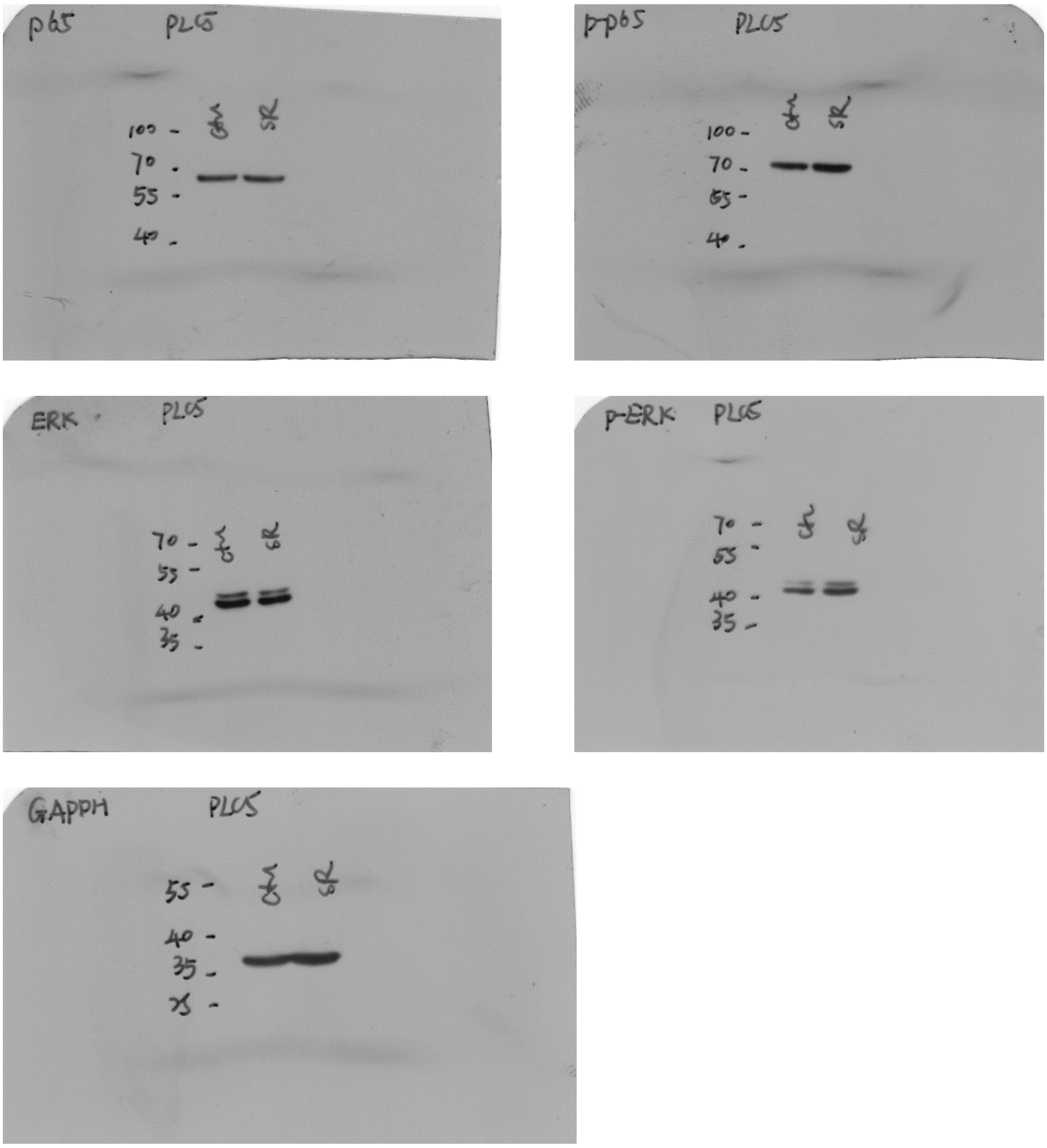

Figure S2A

17-20

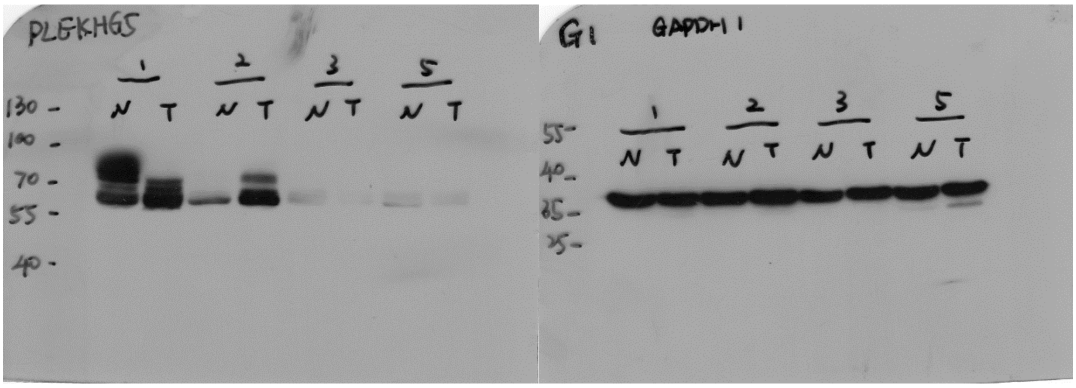

21-24

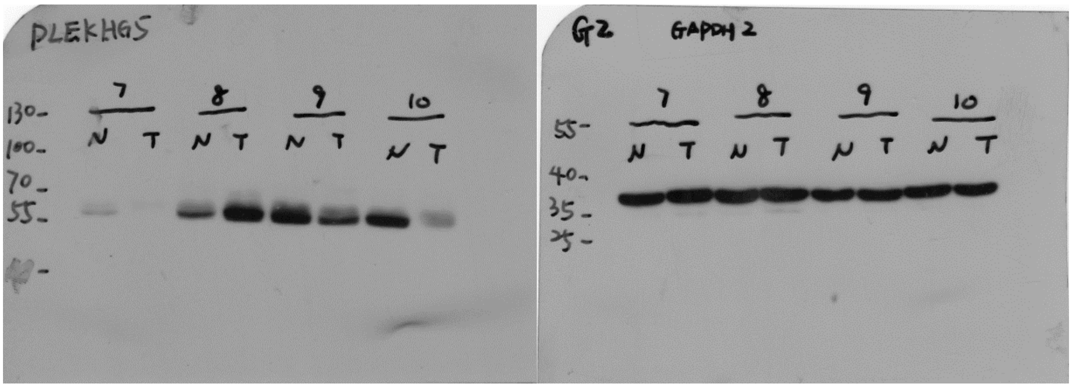

25-28

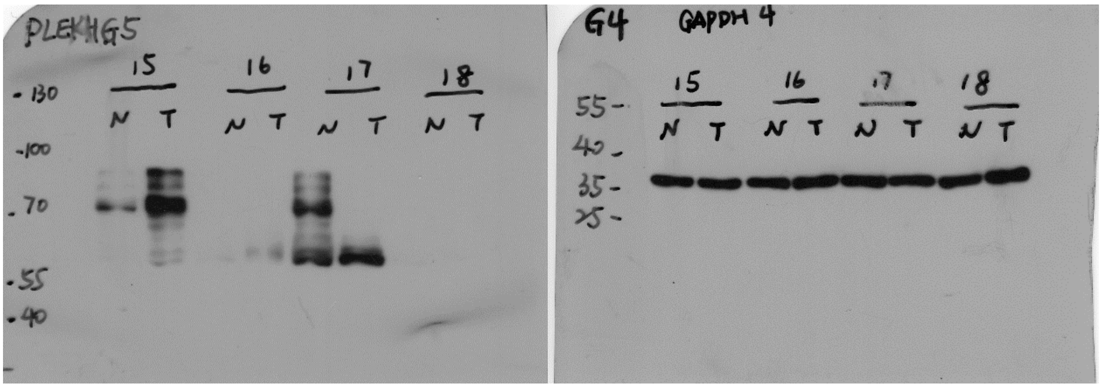

29-32

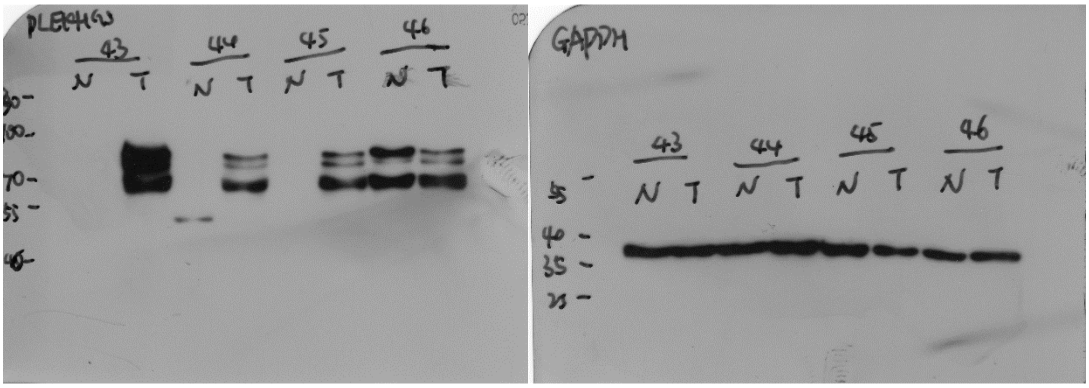

Figure S3A PLC/PRF/5

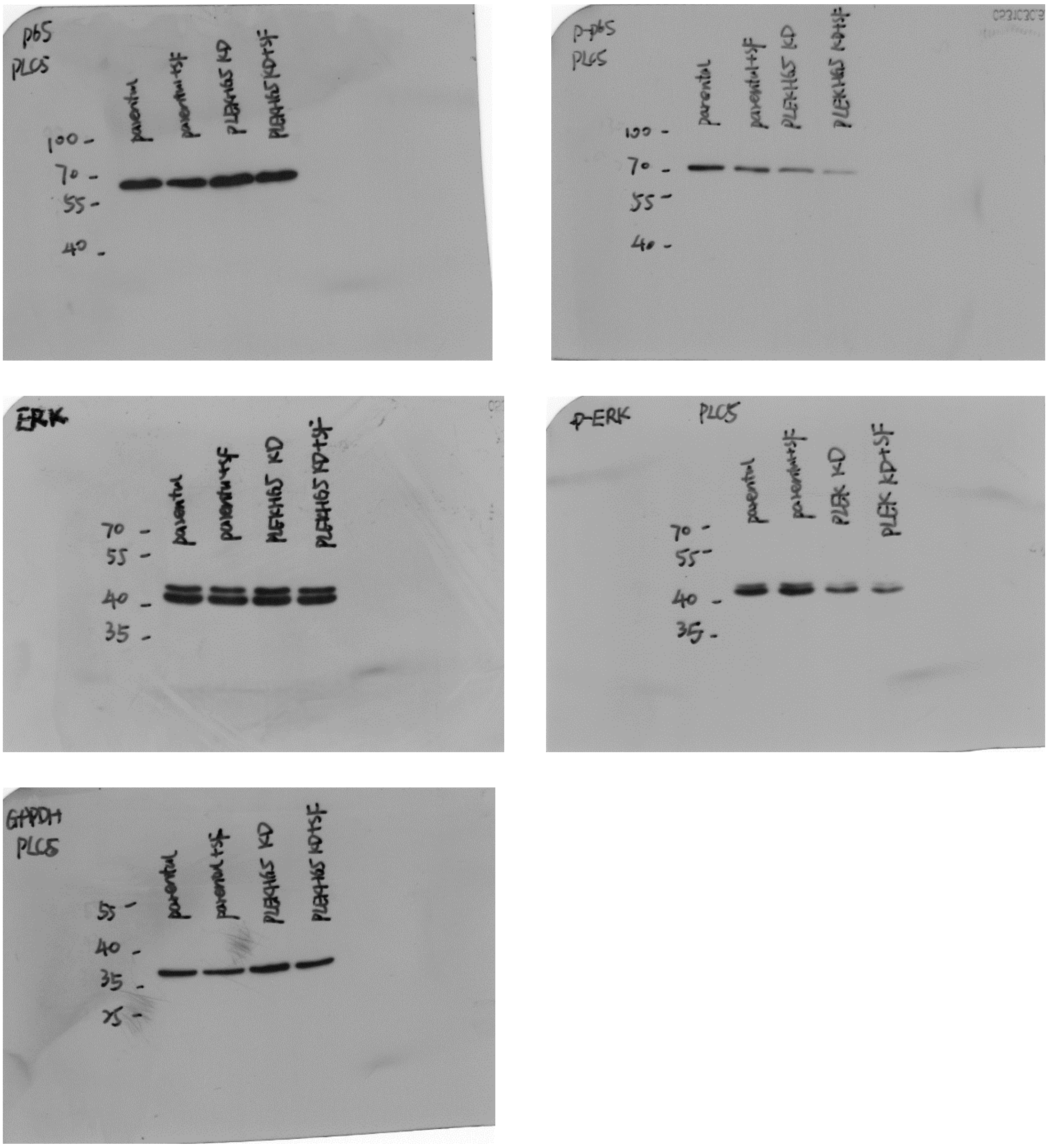

Figure S3A Huh7

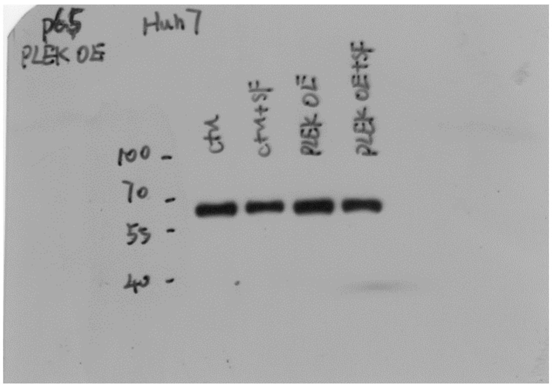

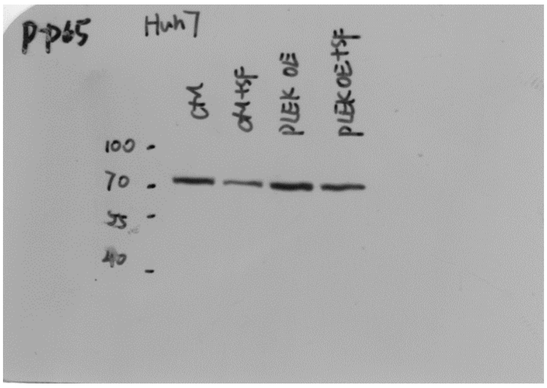

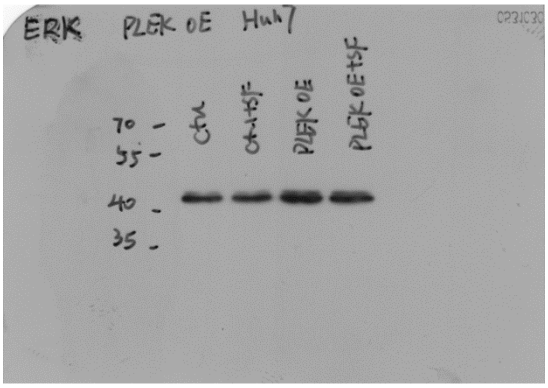

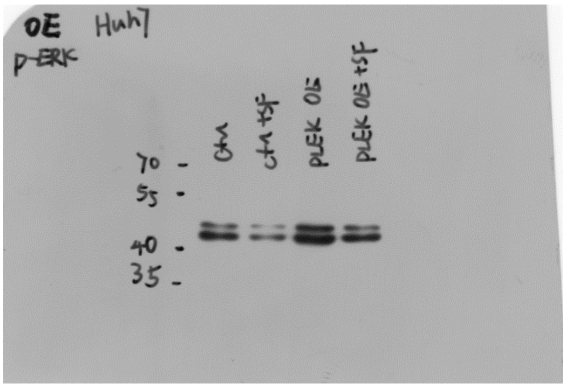

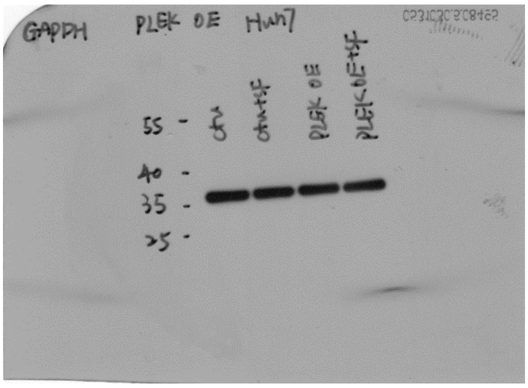

Figure S3B MHCC97H

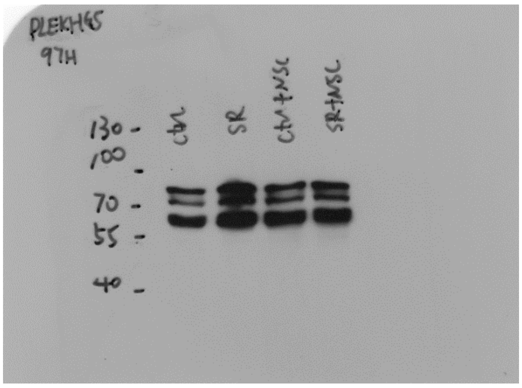

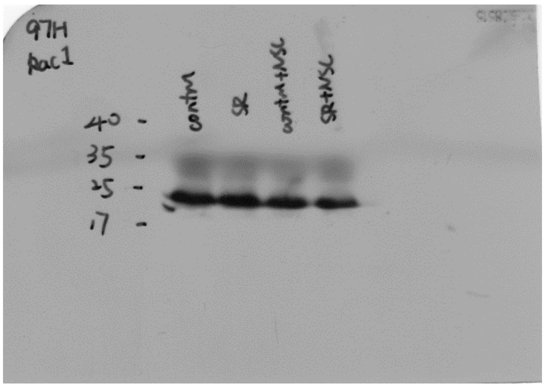

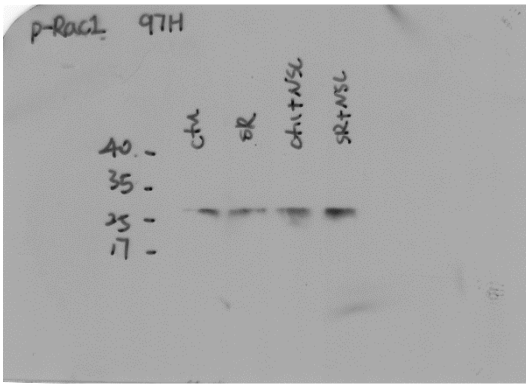

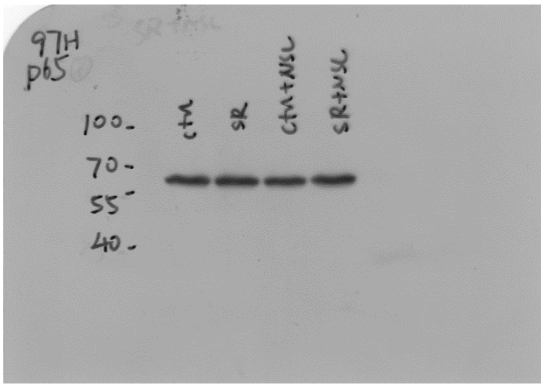

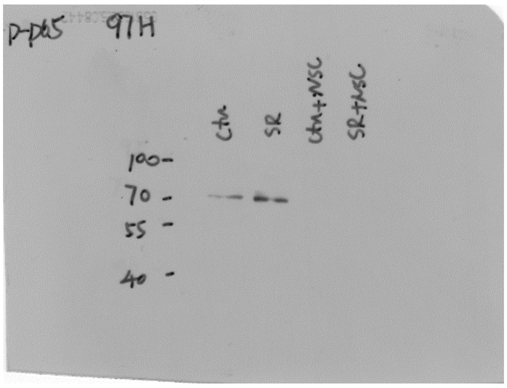

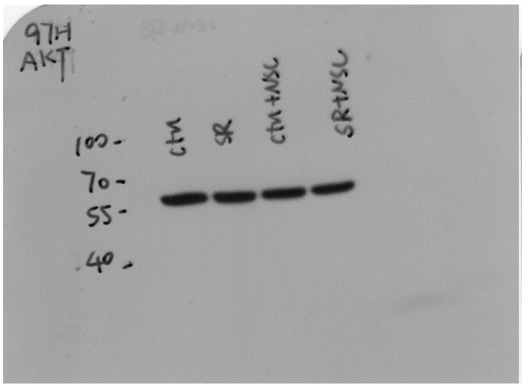

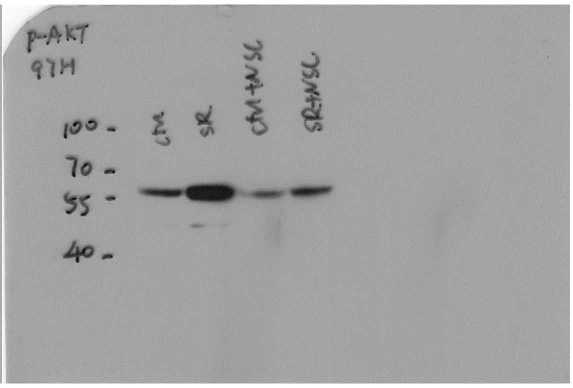

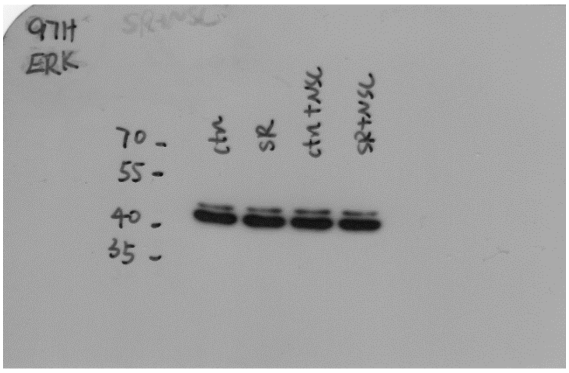
97H
ERK
70
55
40
35
ctrl
SR
ctrl+NSC
SR+NSC

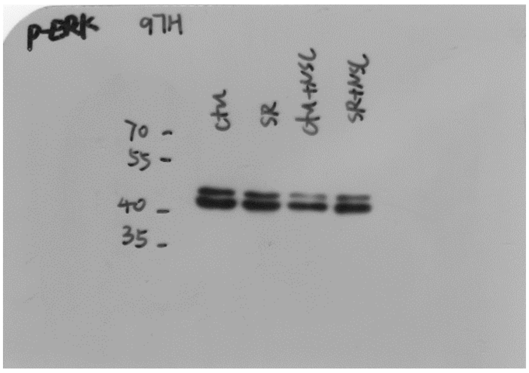
p-ERK 97H
70
55
40
35
ctrl
SR
ctrl+NSC
SR+NSC

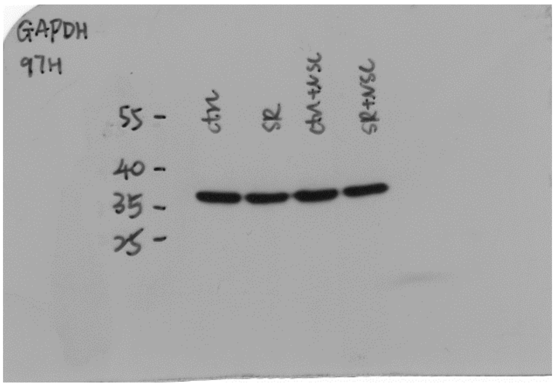
GAPDH
97H
55
40
35
25
ctrl
SR
ctrl+NSC
SR+NSC

Figure S3B PLC/PRF/5

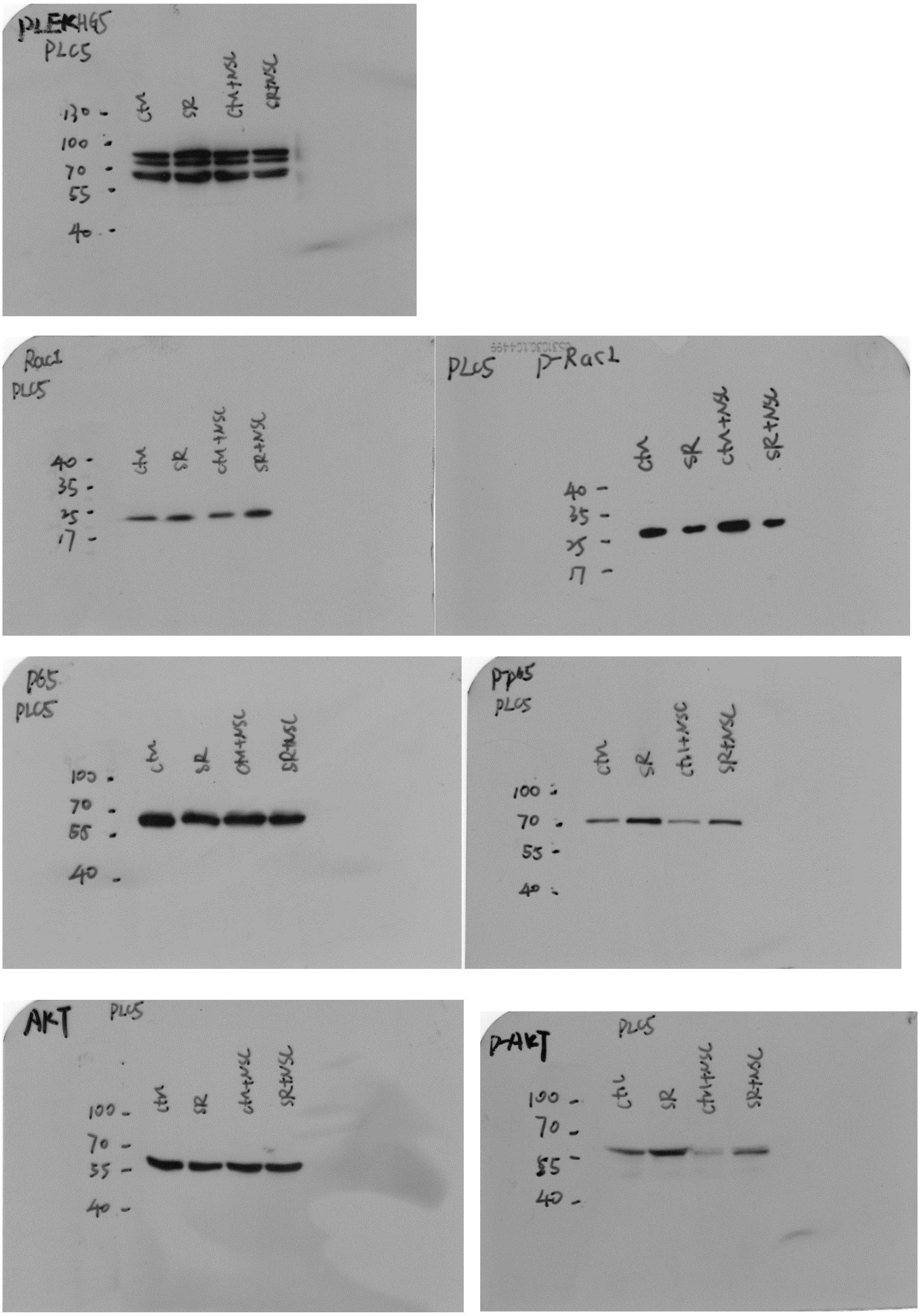

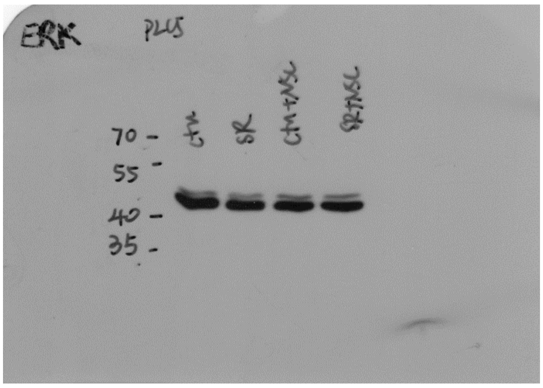
ERK
PLUS
70
55
40
35
Ctrl
SR
Ctrl+MSC
SR+MSC

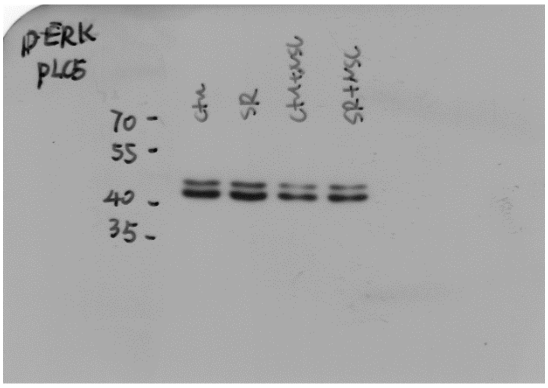
ERK
PLUS
70
55
40
35
Ctrl
SR
Ctrl+MSC
SR+MSC

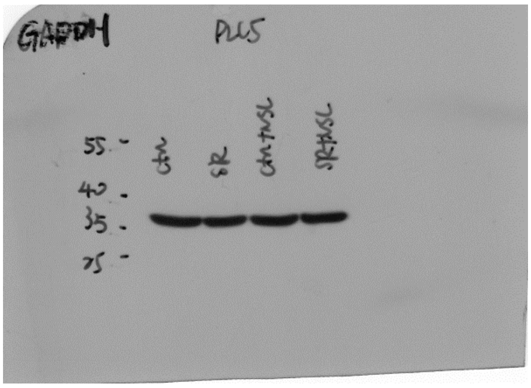
GAPDH
PLUS
55
40
35
25
Ctrl
SR
Ctrl+MSC
SR+MSC

Figure S3D Huh7

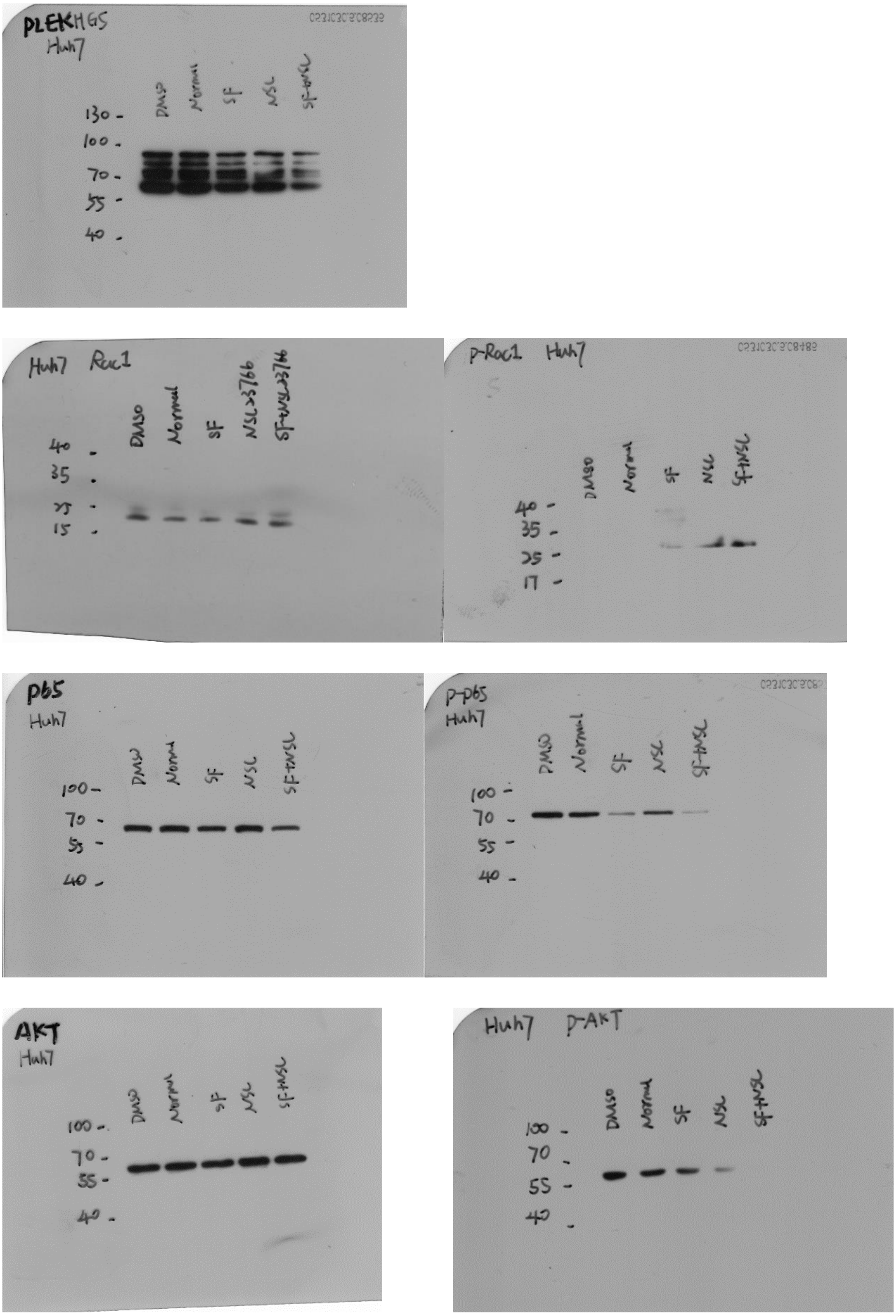

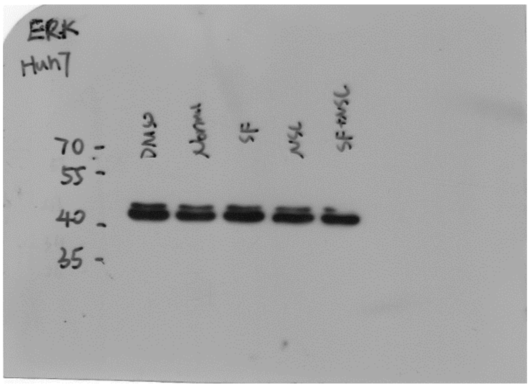
ERK
Huh7
DMSO
Normal
SF
NSC
SF+NSC
70
55
40
35

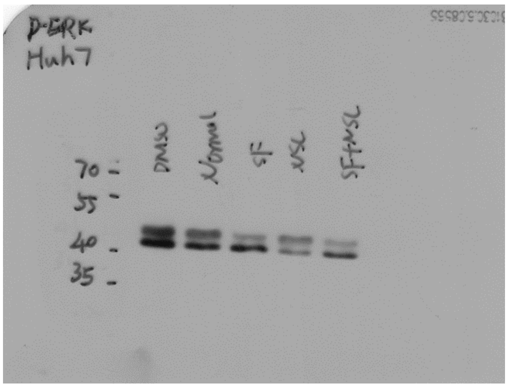
p-ERK
Huh7
DMSO
Normal
SF
NSC
SF+NSC
70
55
40
35

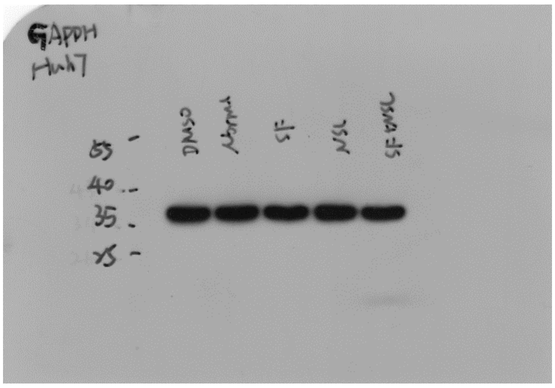
GAPDH
Huh7
DMSO
Normal
SF
NSC
SF+NSC
55
40
35
25

Figure S3E MHCC97H

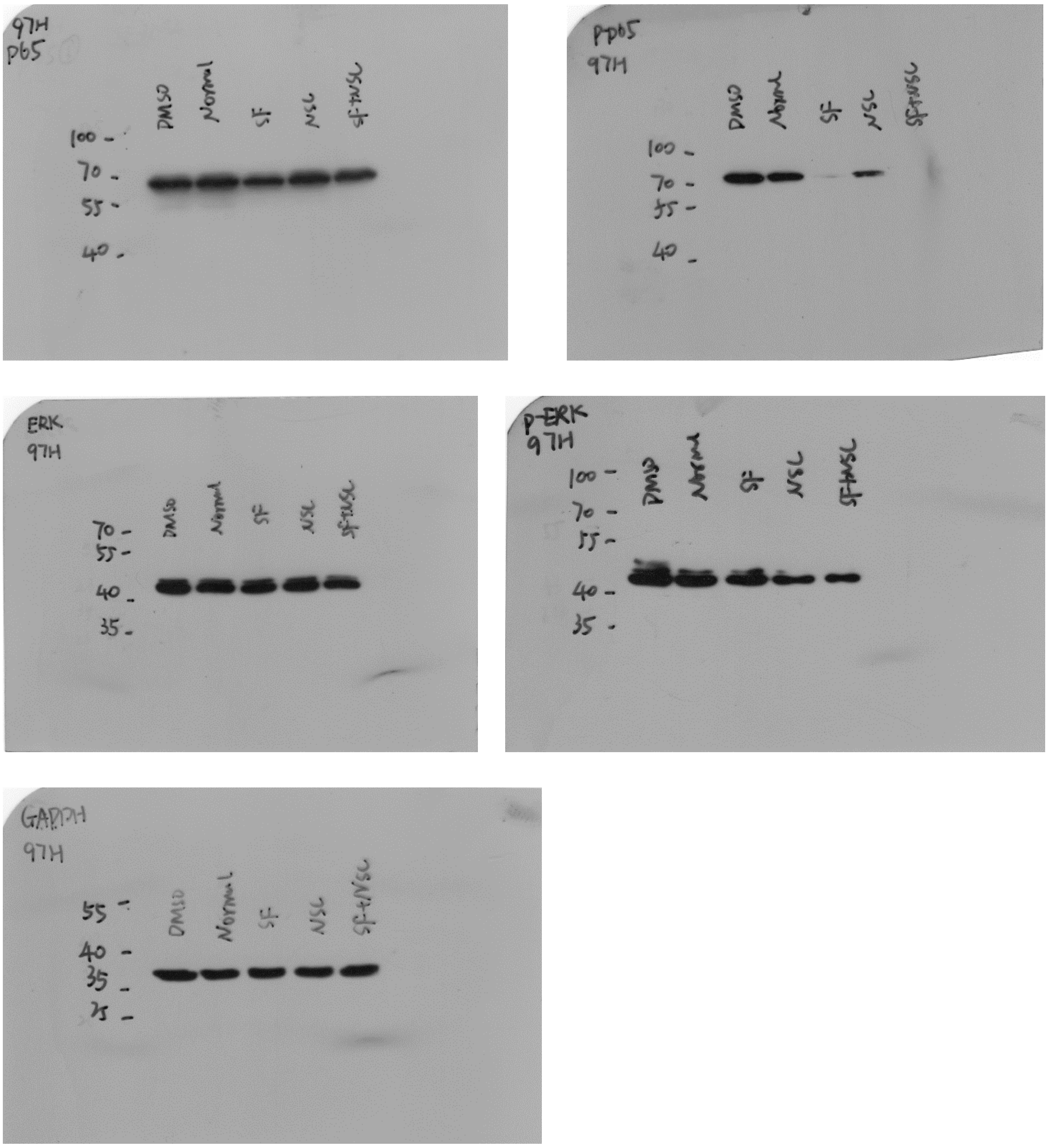

Figure S3E MHCC97H SR

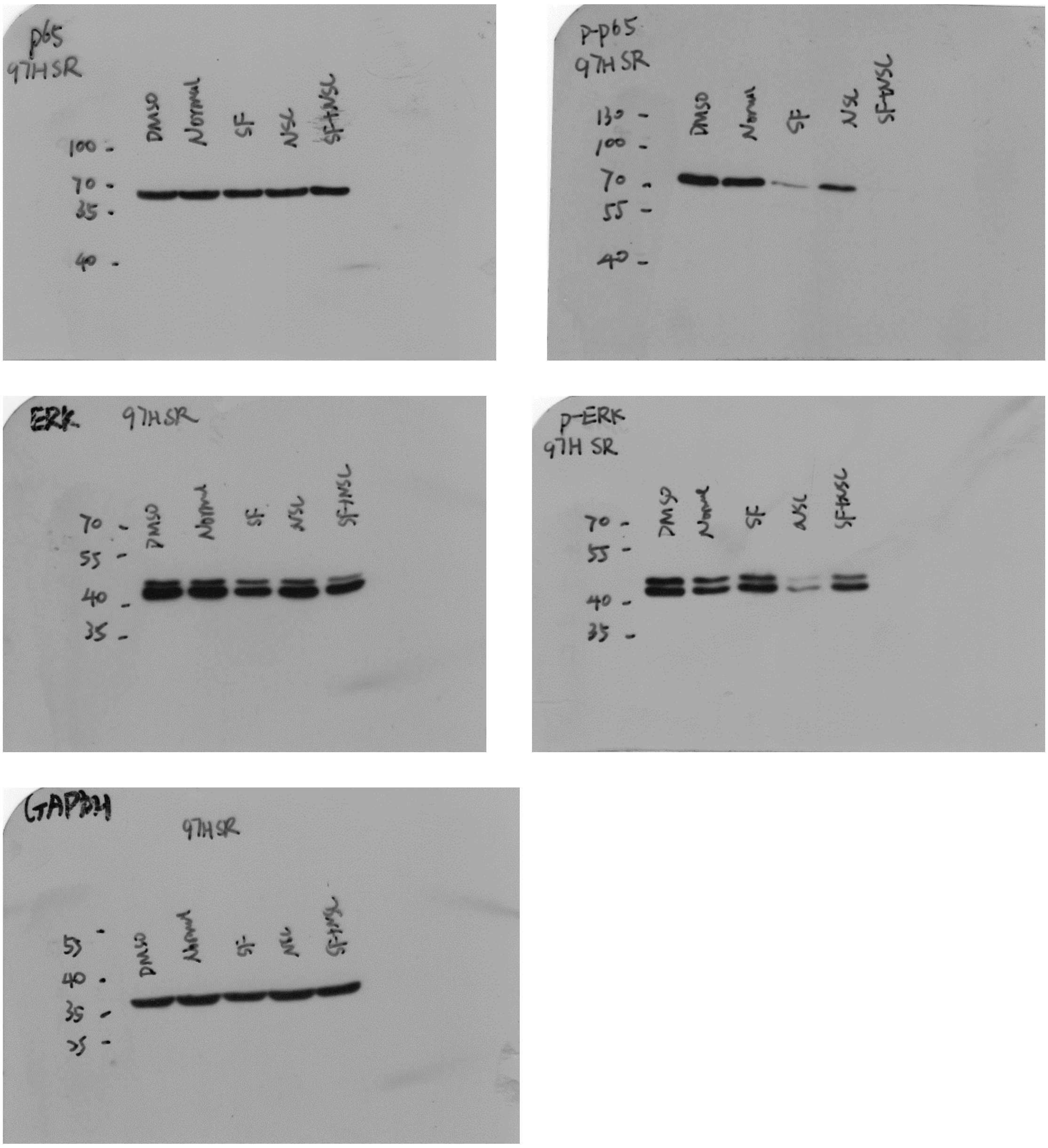

Figure S3E PLC/PRF/5

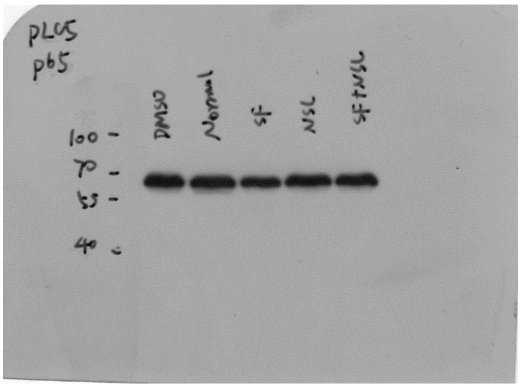

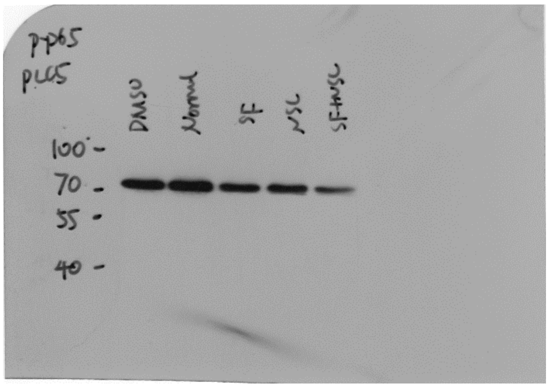

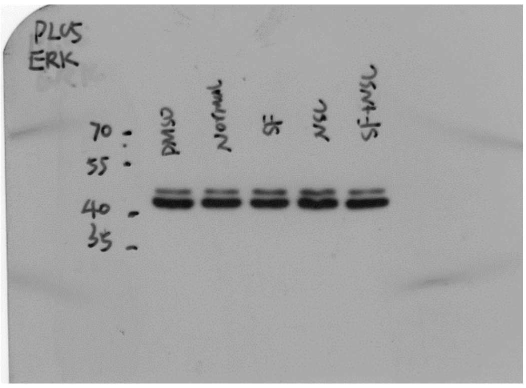

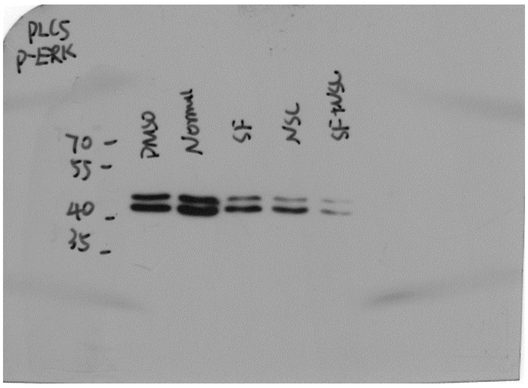

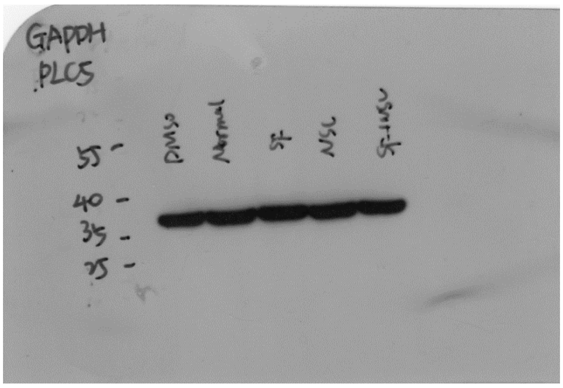

Figure S3E PLC/PRF/5 SR

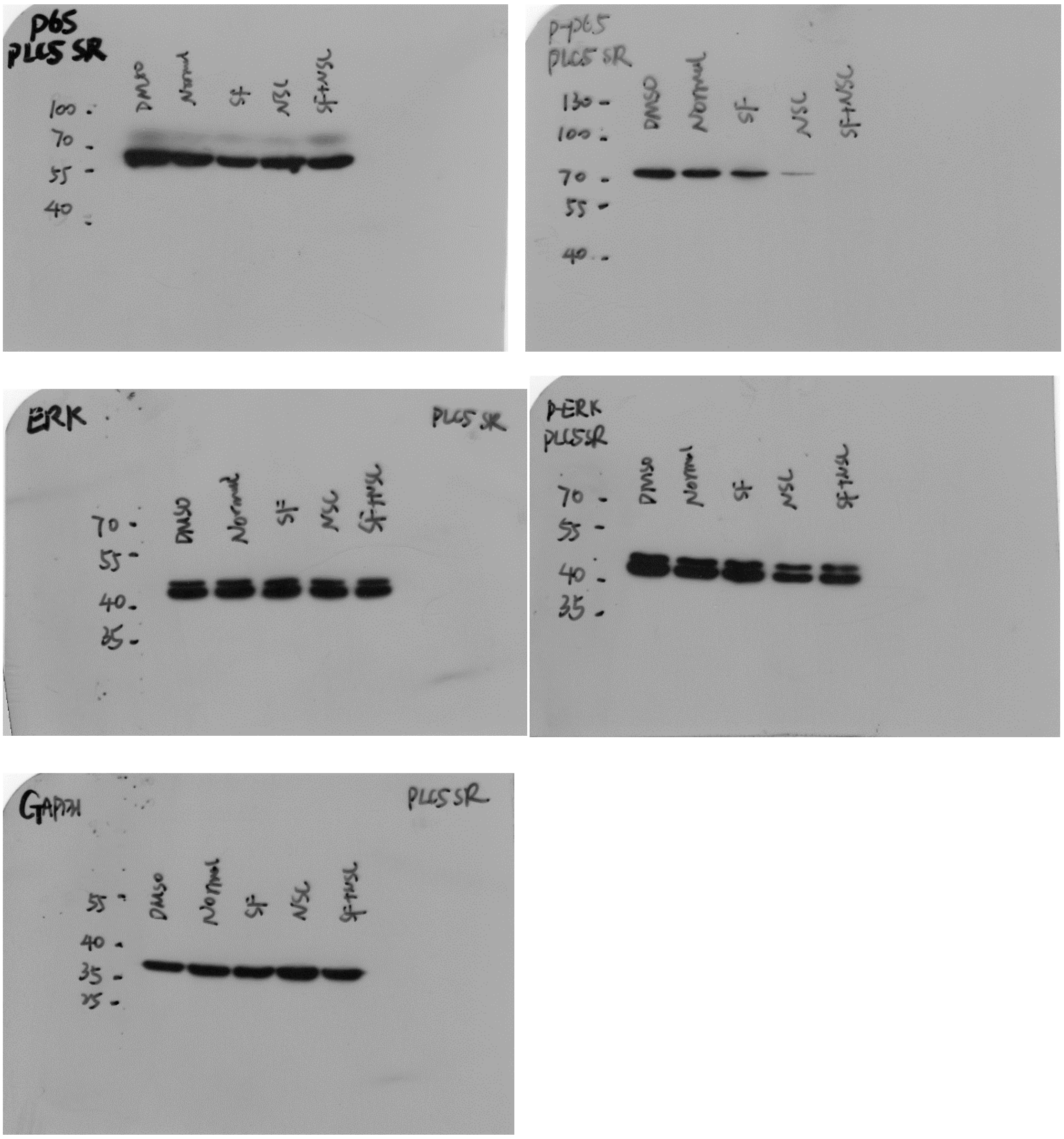

Figure S4A PLC/PRF/5

Input ACE

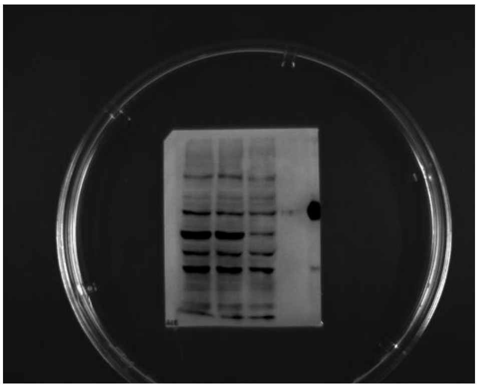

Input-Flag

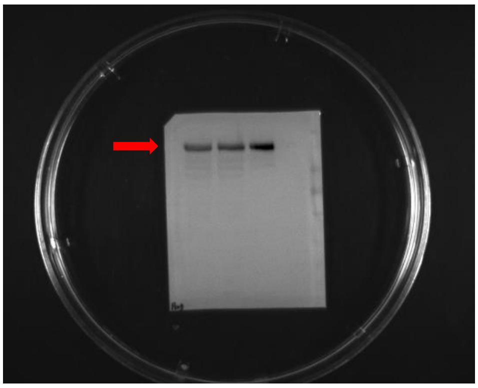

Input GAPDH

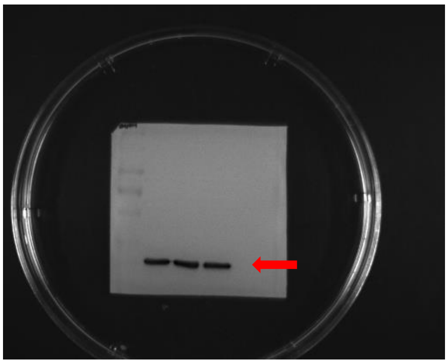

IP-ACE

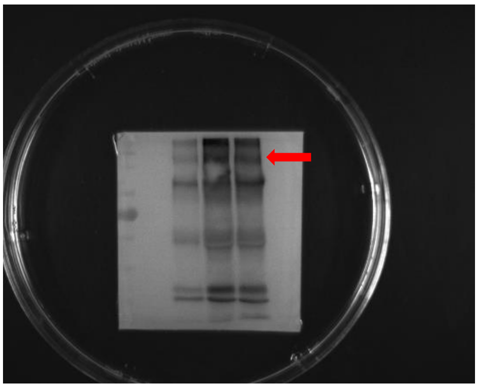

IP-Flag

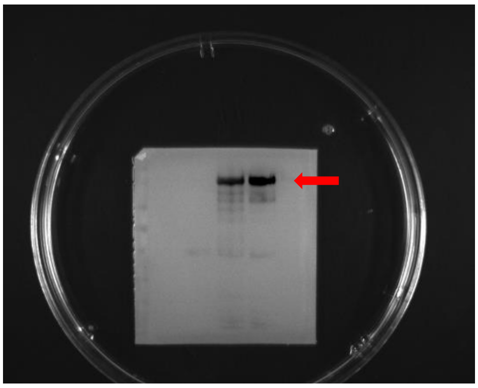

Figure S4B PLC/PRF/5

Input-Flag

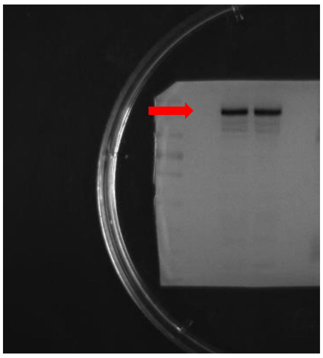

Input-HDAC2

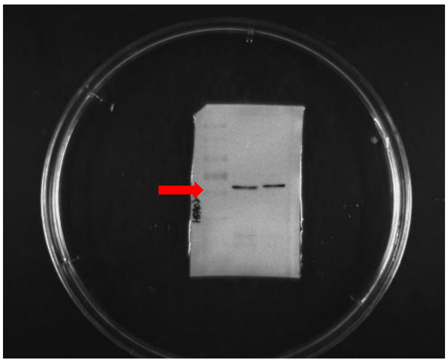

IP-Flag

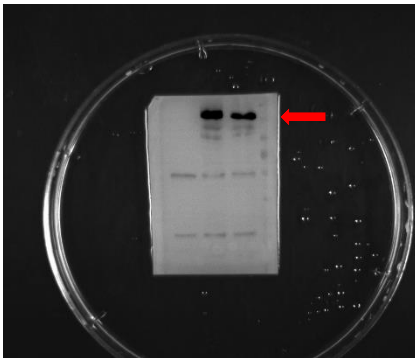

IP-HDAC2

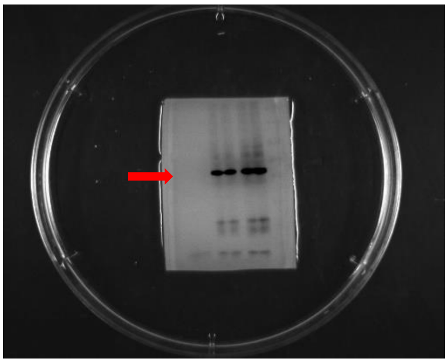

Figure S4D PLC/PRF/5

Input-Flag

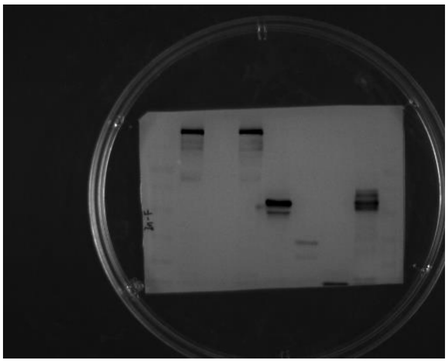

Input-HDAC2

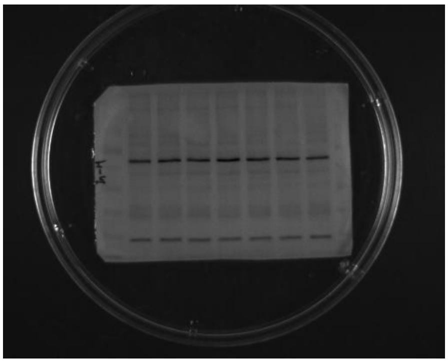

HDAC2-IP-Flag

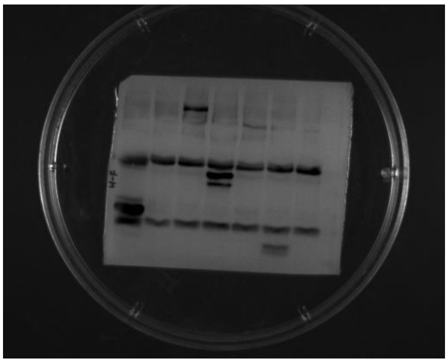

HDAC2-IP-HDAC2

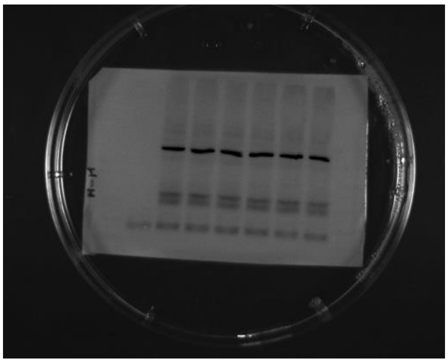

Flag-IP-Flag

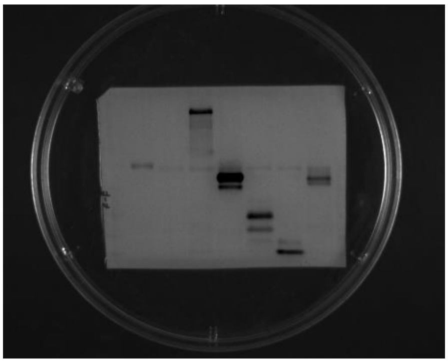

Flag-IP-HDAC2

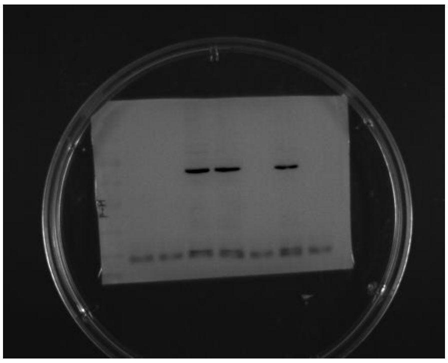

Figure S5B

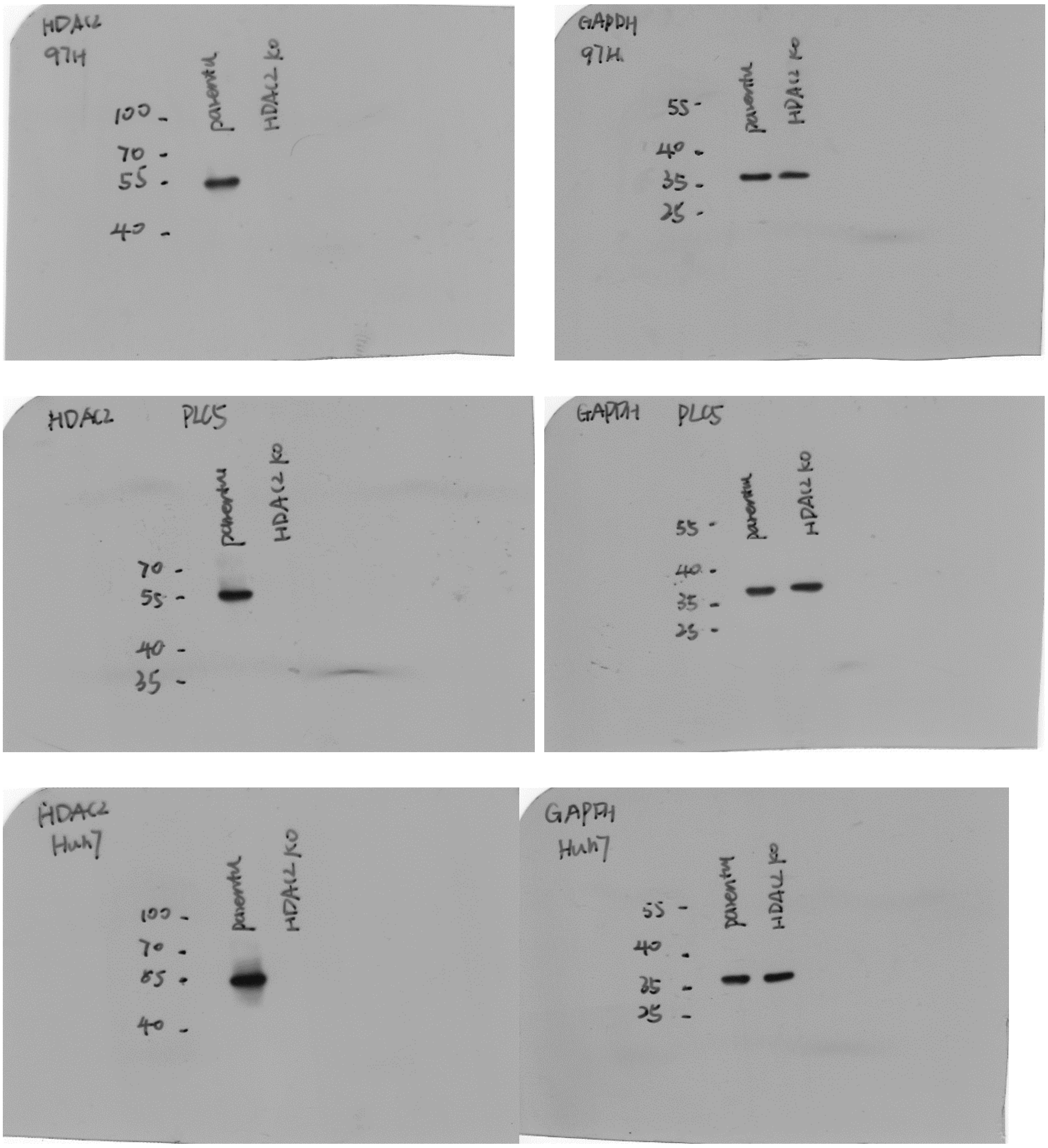
HDAC2
97H
parental
HDAC2 KO
100
70
55
40
GAPDH
97H
55
40
35
25
HDAC2
PLC5
70
55
40
35
GAPDH
PLC5
HDAC2
Huh7
100
70
40
GAPDH
Huh7

Figure S5C Huh7

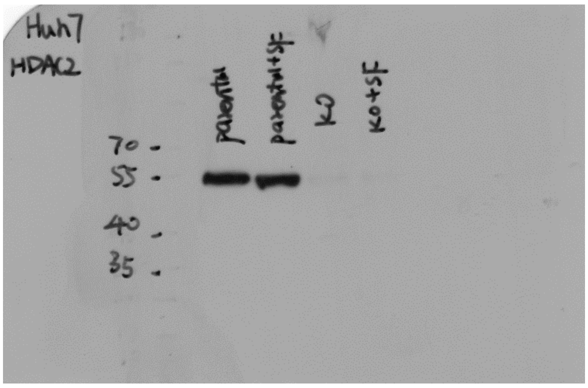

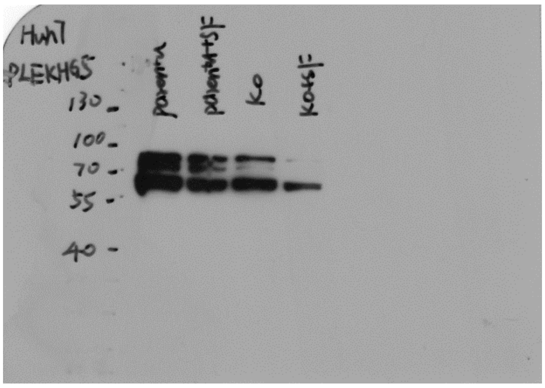

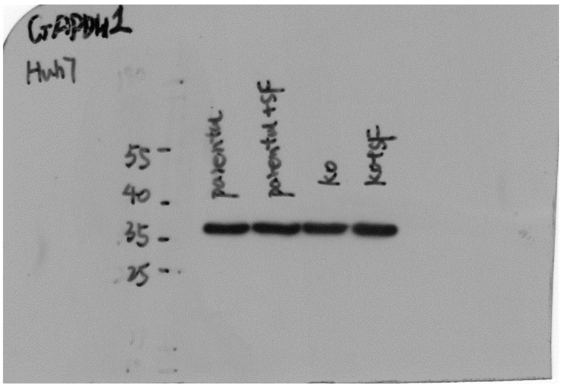

Figure S5D PLC/PRF/5

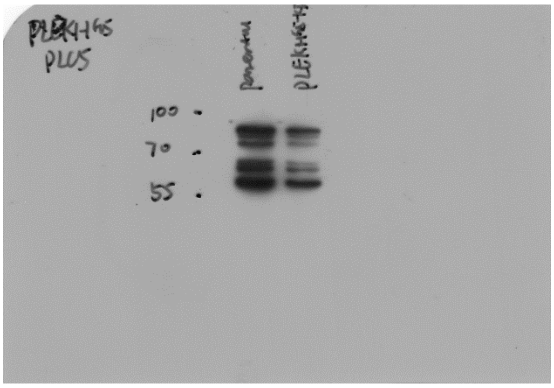

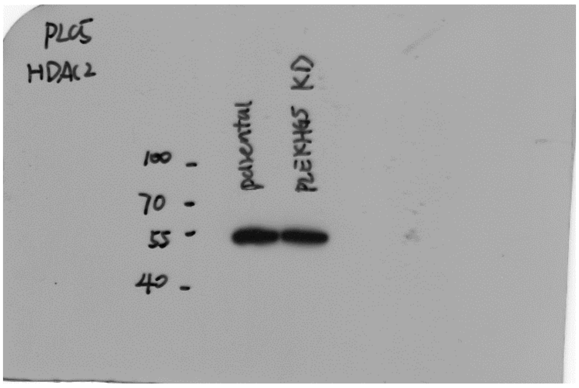

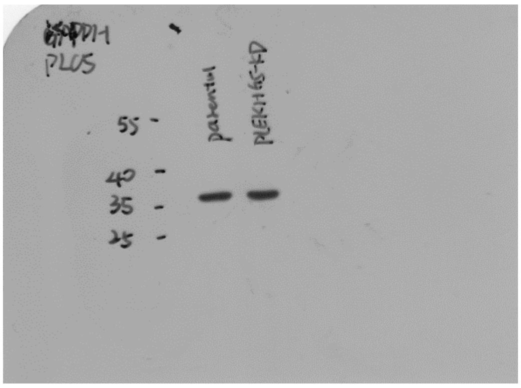

Figure S5E MHCC97H

WT+K1Q

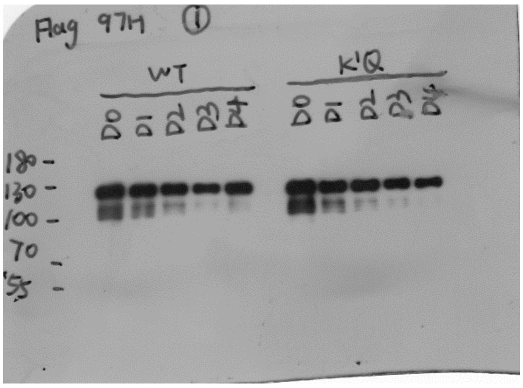

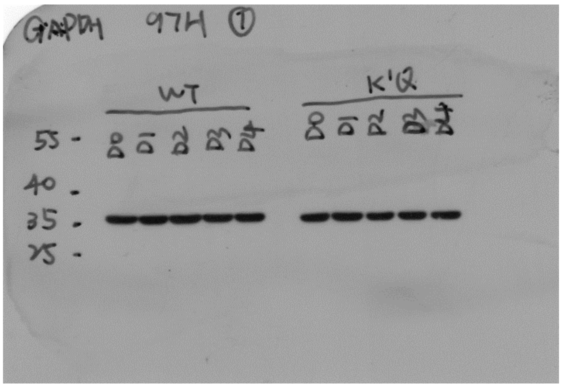

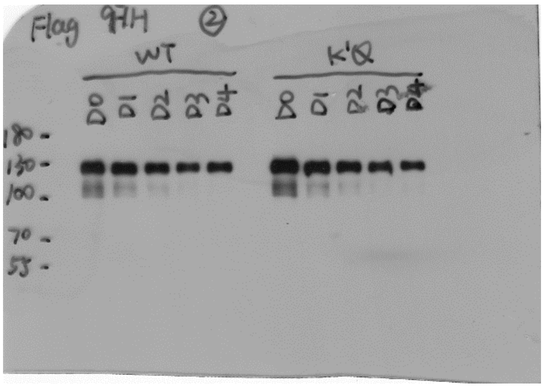

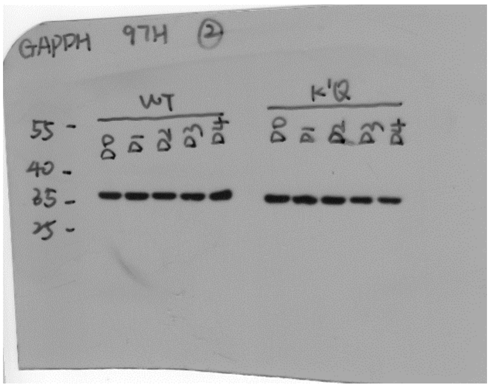

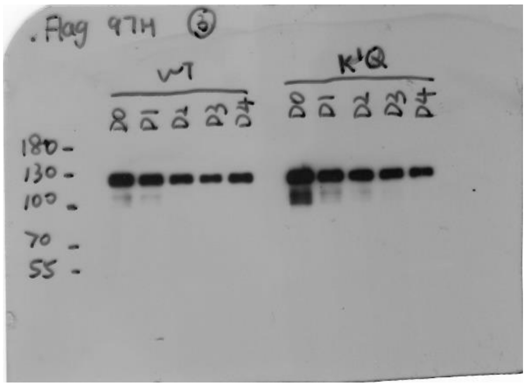

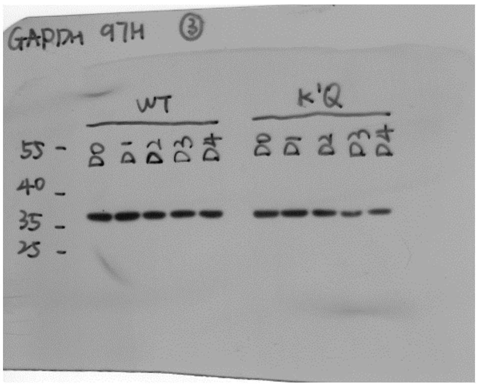

K2Q+K3Q

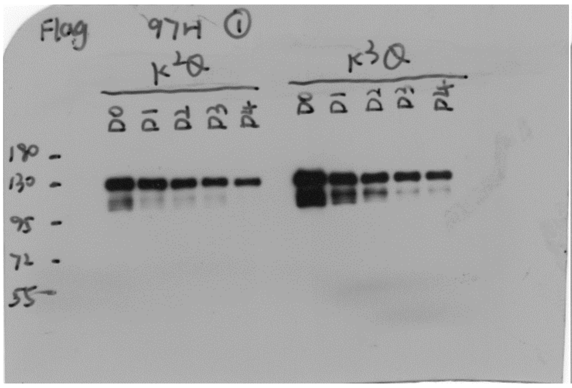

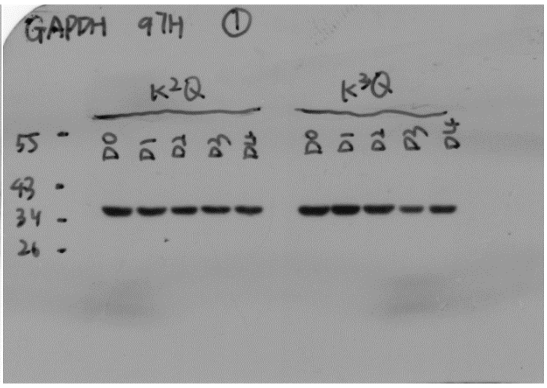

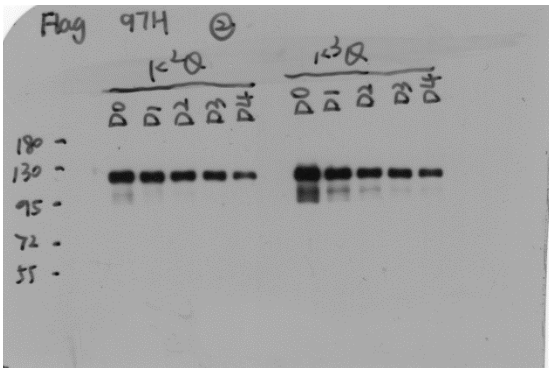
Flag 97H ②
K²Q
K³Q
D0 D1 D2 D3 D4
180
130
95
72
55

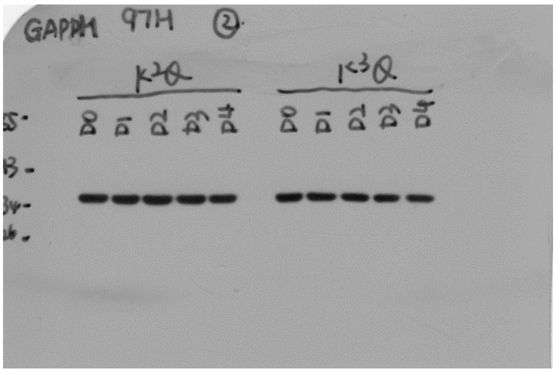
GAPDH 97H ②
K²Q
K³Q
D0 D1 D2 D3 D4
43
34
26

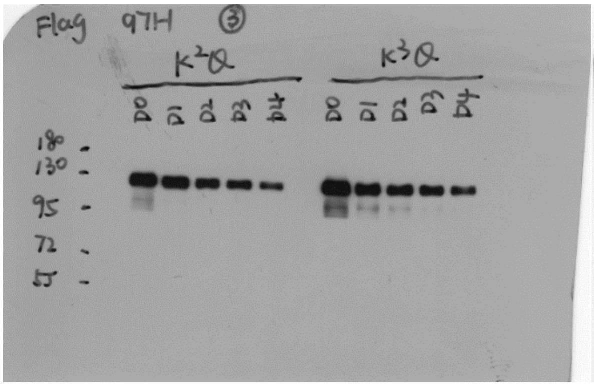
Flag 97H ③
K²Q
K³Q
D0 D1 D2 D3 D4
180
130
95
72
55

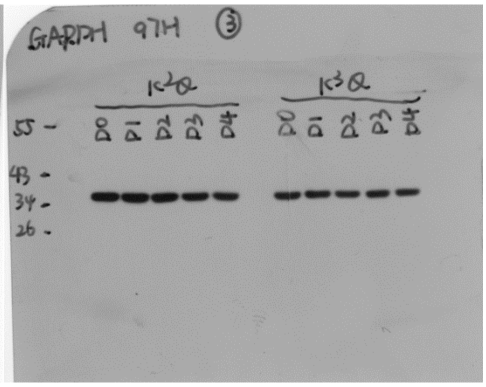
GAPDH 97H ③
K²Q
K³Q
D0 D1 D2 D3 D4
55
43
34
26

3KQ

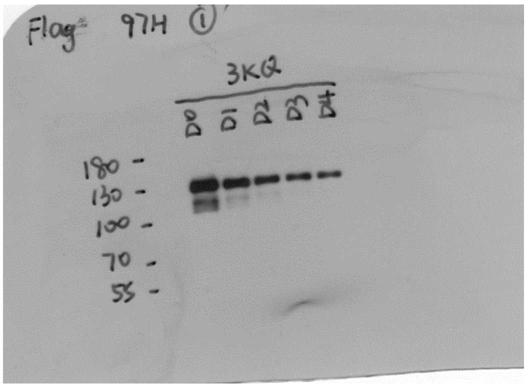
Flag 97H ①
3KQ
D0 D1 D2 D3 D4
180
130
100
70
55

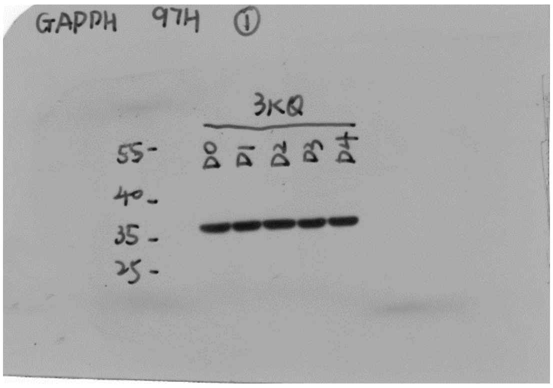
GAPDH 97H ①
3KQ
D0 D1 D2 D3 D4
55
40
35
25

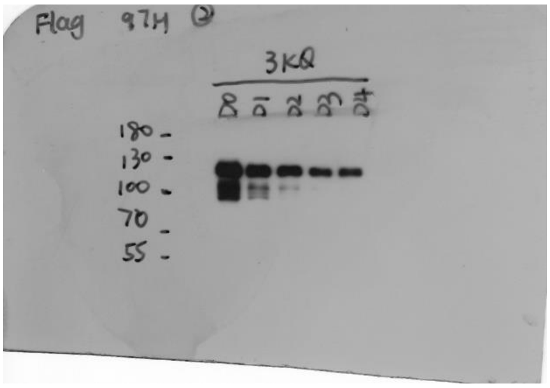
Flag 97H ②
3KQ
D0 D1 D2 D3 D4
180
130
100
70
55

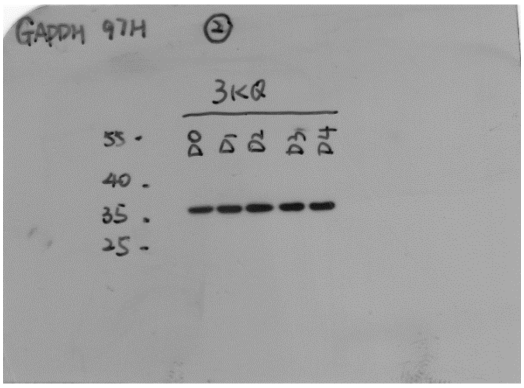
GAPDH 97H ②
3KQ
D0 D1 D2 D3 D4
55
40
35
25

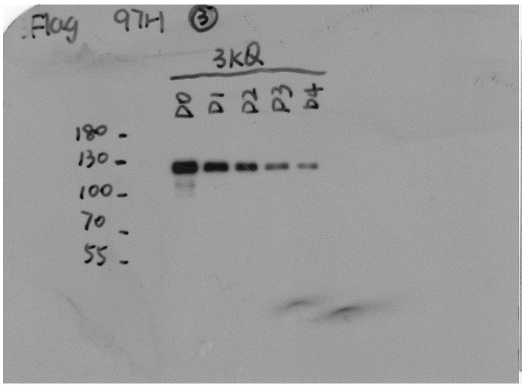
Flag 97H ③
3KQ
D0 D1 D2 D3 D4
180
130
100
70
55

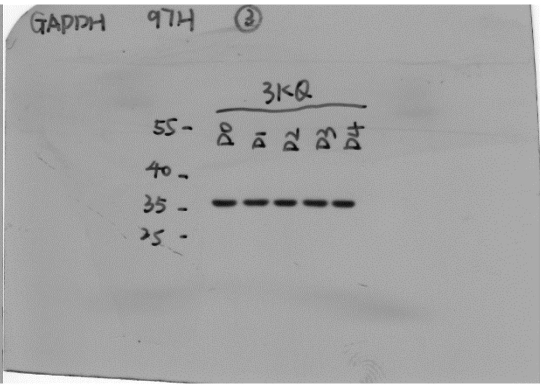
GAPDH 97H ③
3KQ
D0 D1 D2 D3 D4
55
40
35
25

Figure S5F PLC/PRF/5

WT+K1Q

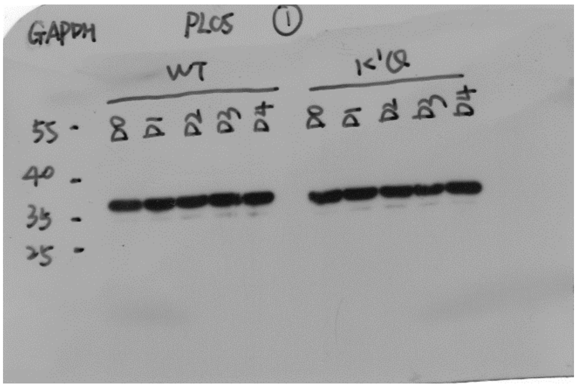

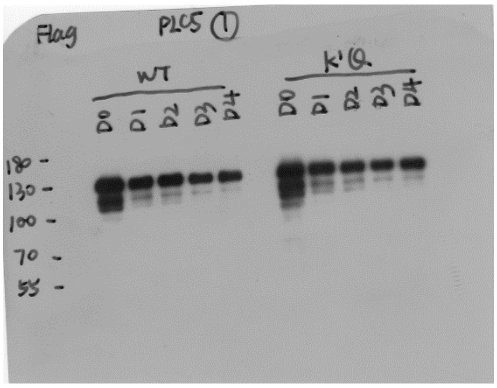

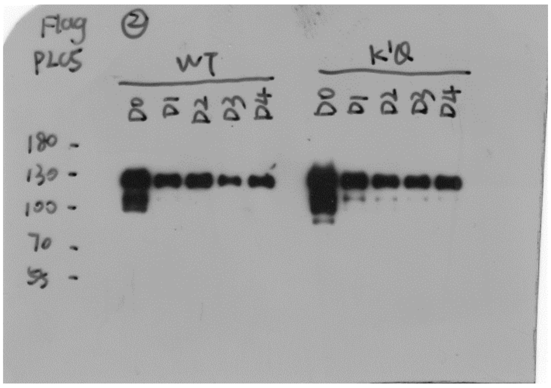

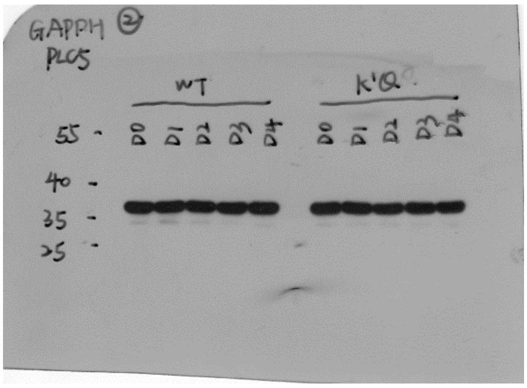

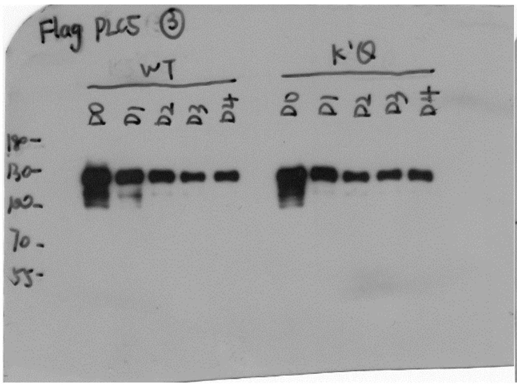

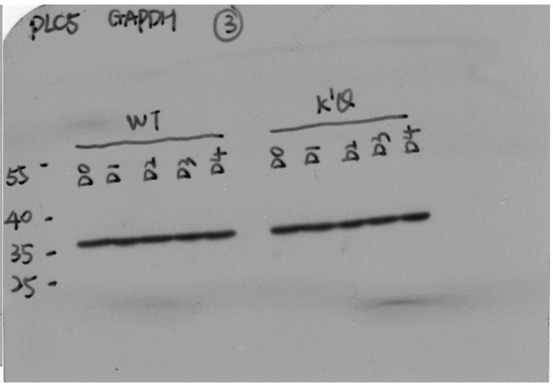

K2Q+K3Q

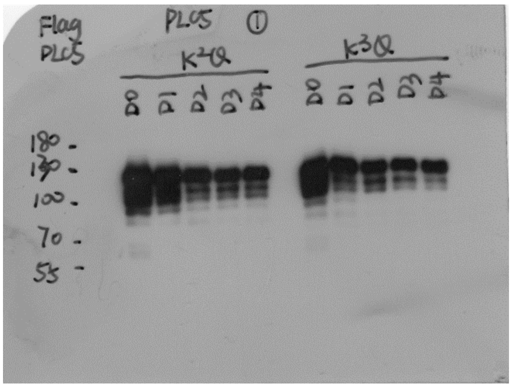

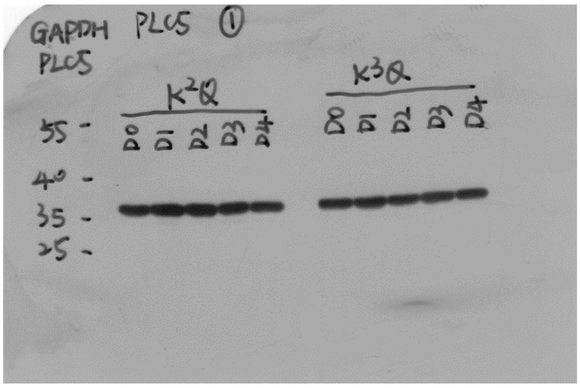

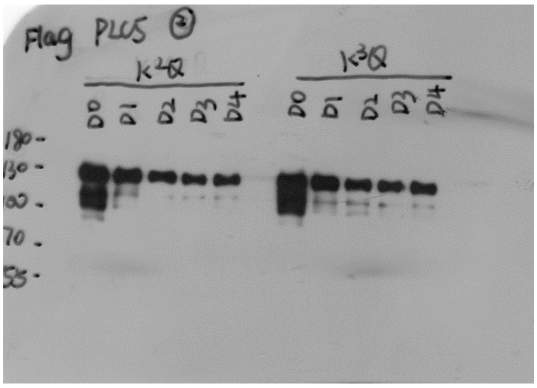
Flag PLC5 ②
K²Q
K³Q
D0 D1 D2 D3 D4+
D0 D1 D2 D3 D4+
180
130
100
70
55

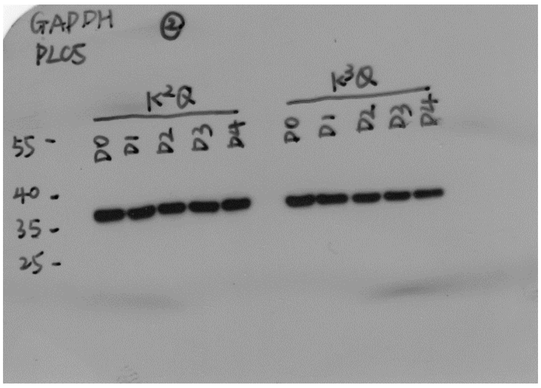
GAPDH ②
PLC5
K²Q
K³Q
D0 D1 D2 D3 D4+
D0 D1 D2 D3 D4+
55
40
35
25

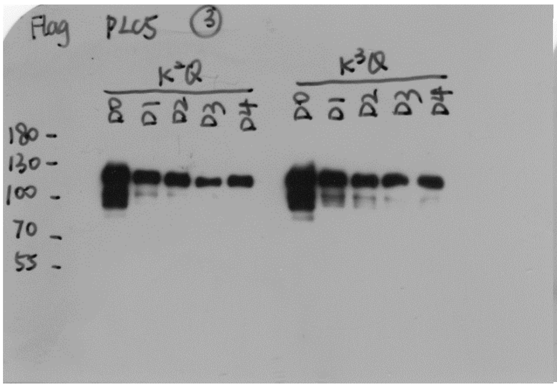
Flag PLC5 ③
K²Q
K³Q
D0 D1 D2 D3 D4+
D0 D1 D2 D3 D4+
180
130
100
70
55

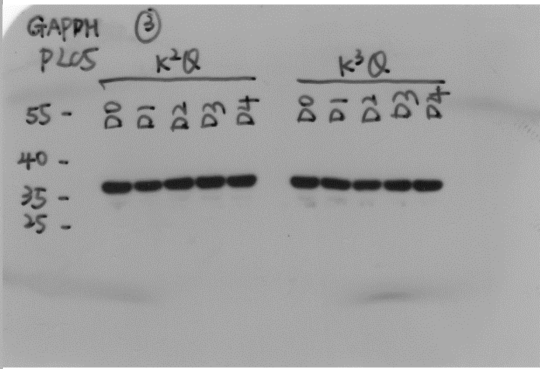
GAPDH ③
PLC5
K²Q
K³Q
D0 D1 D2 D3 D4+
D0 D1 D2 D3 D4+
55
40
35
25

3KQ

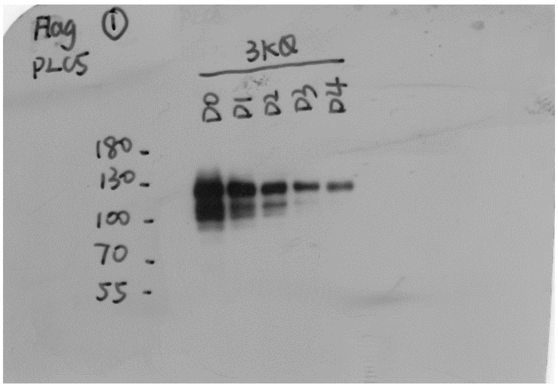
Flag ①
PLC5
3KQ
D0 D1 D2 D3 D4+
180
130
100
70
55

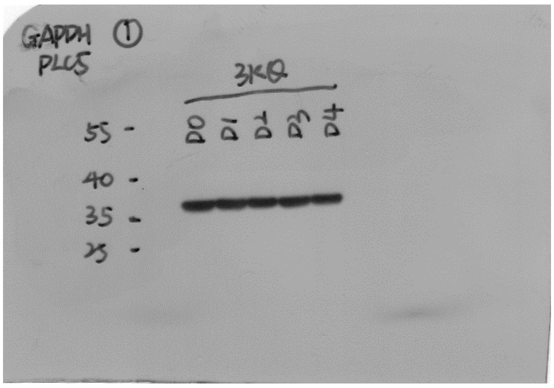
GAPDH ①
PLC5
3KQ
D0 D1 D2 D3 D4+
55
40
35
25

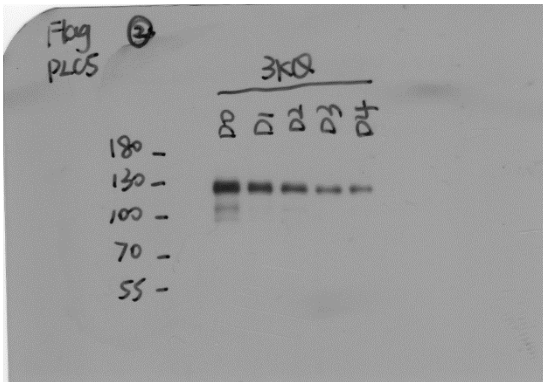
Flag ②
PLC5
3KQ
D0 D1 D2 D3 D4+
180
130
100
70
55

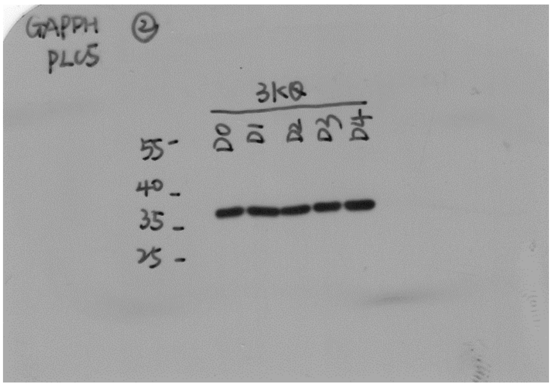
GAPDH ②
PLC5
3KQ
D0 D1 D2 D3 D4+
55
40
35
25

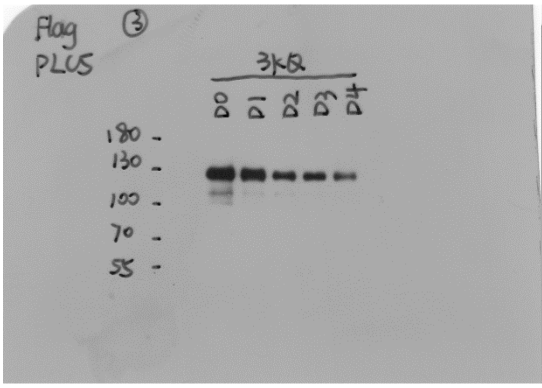
Flag ③
PLC5
3KQ
D0 D1 D2 D3 D4
180
130
100
70
55
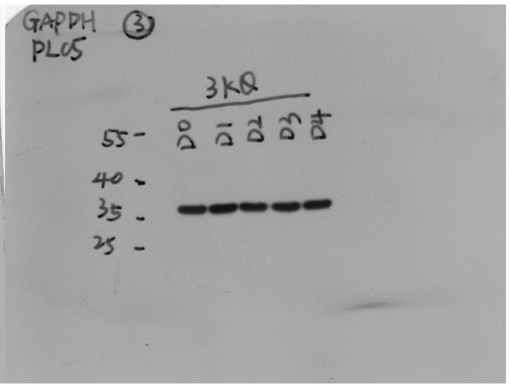
GAPDH ③
PLC5
3KQ
D0 D1 D2 D3 D4
55
40
35
25

Figure S5G MHCC97H HDAC2 KO

WT+K1R

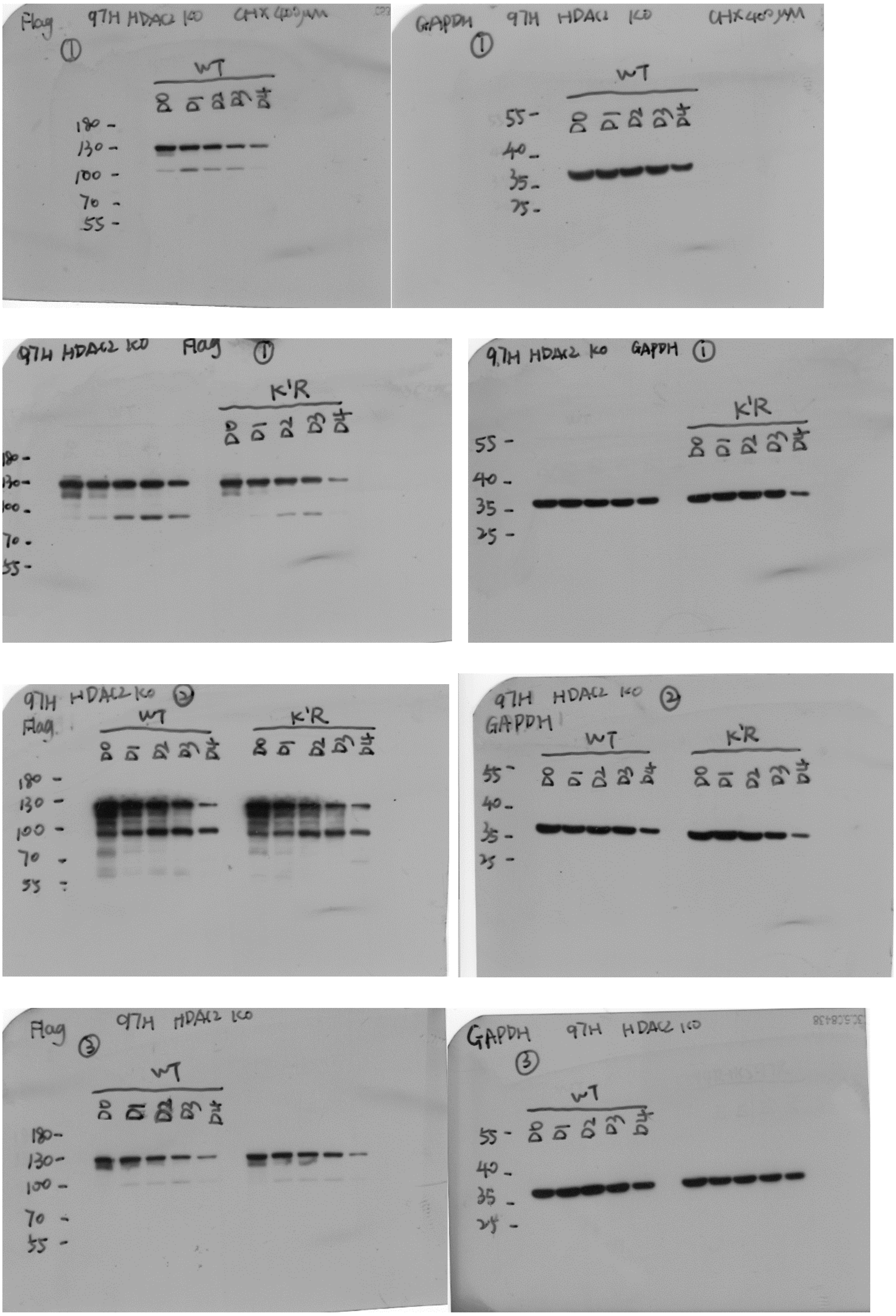

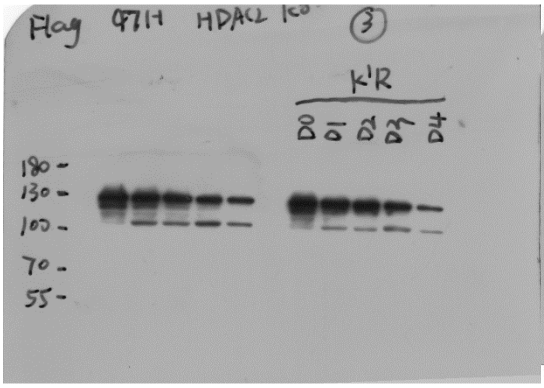

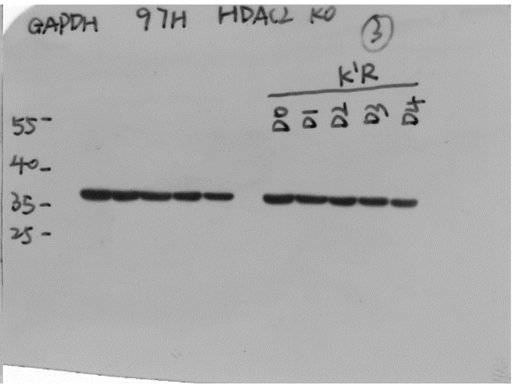

K2R+K3R

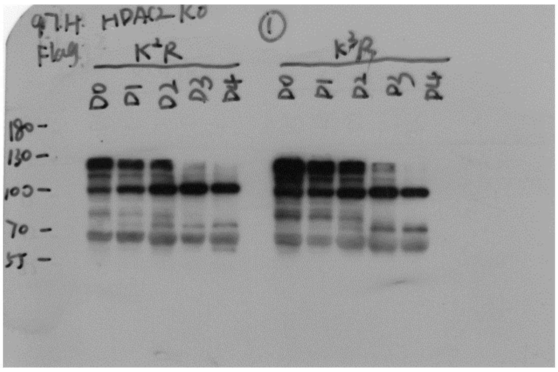

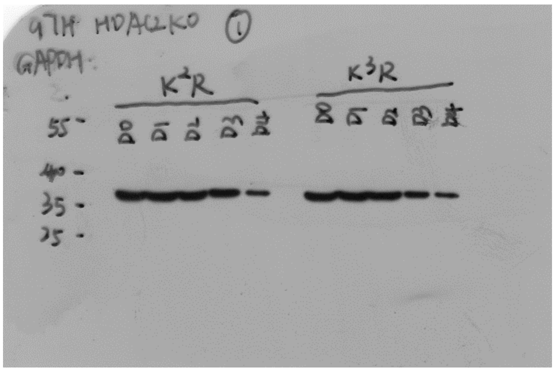

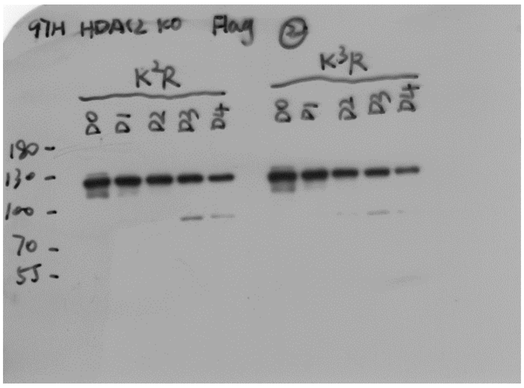

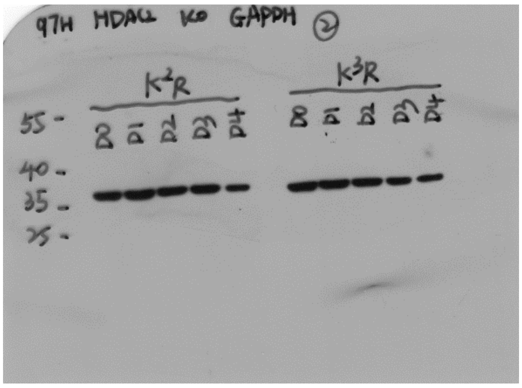

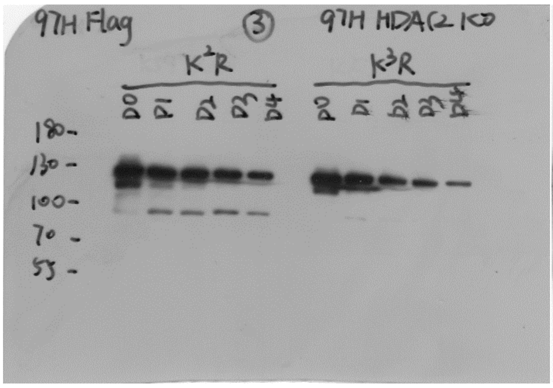

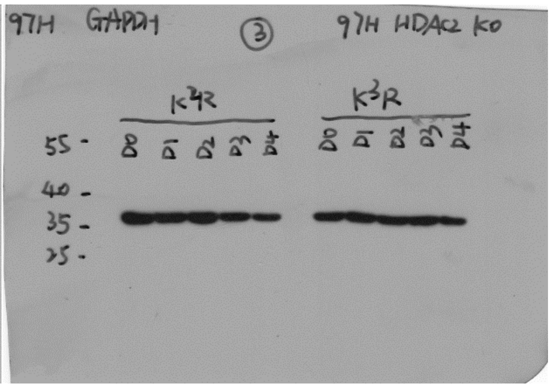

3KR

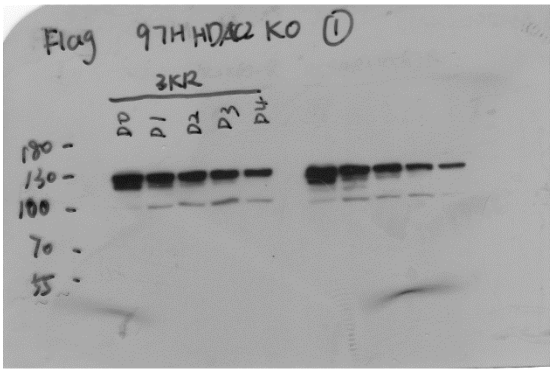
Flag 97H HDAC2 KO ①
3KR
D0 D1 D2 D3 D4
180
130
100
70
55

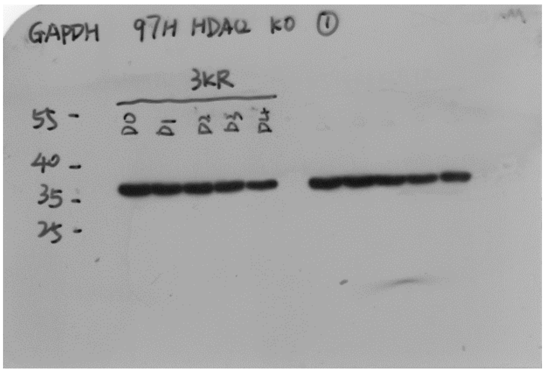
GAPDH 97H HDAC2 KO ①
3KR
D0 D1 D2 D3 D4
55
40
35
25

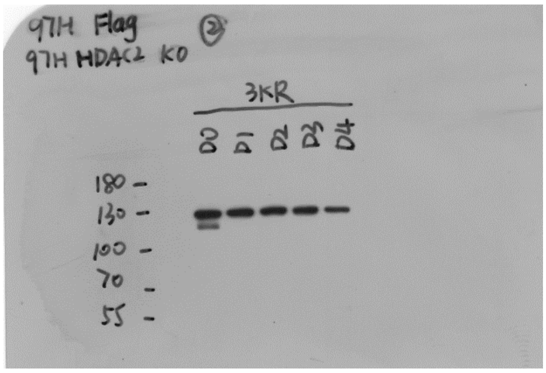
97H Flag ②
97H HDAC2 KO
3KR
D0 D1 D2 D3 D4
180
130
100
70
55

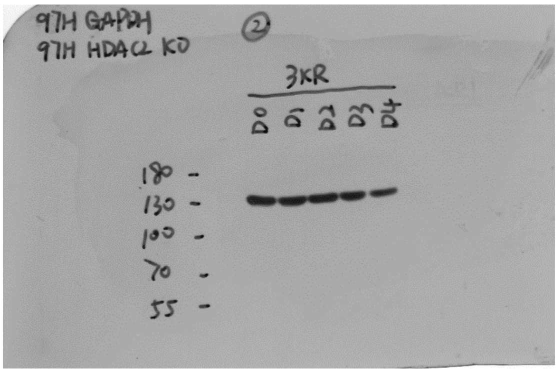
97H GAPDH ②
97H HDAC2 KO
3KR
D0 D1 D2 D3 D4
180
130
100
70
55

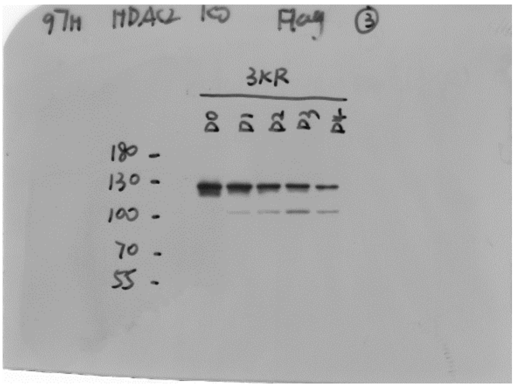
97H HDAC2 KO Flag ③
3KR
D0 D1 D2 D3 D4
180
130
100
70
55

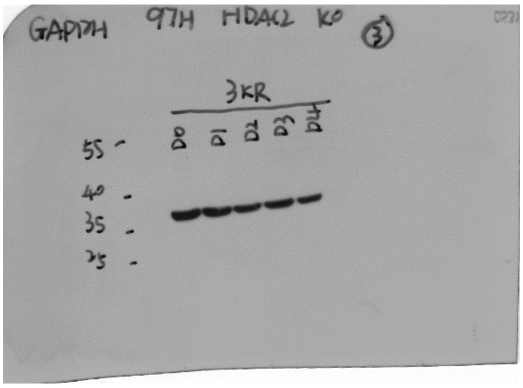
GAPDH 97H HDAC2 KO ③
3KR
D0 D1 D2 D3 D4
55
40
35
25

Figure S5H PLC/PRF/5 HDAC2 KO

WT+K1R

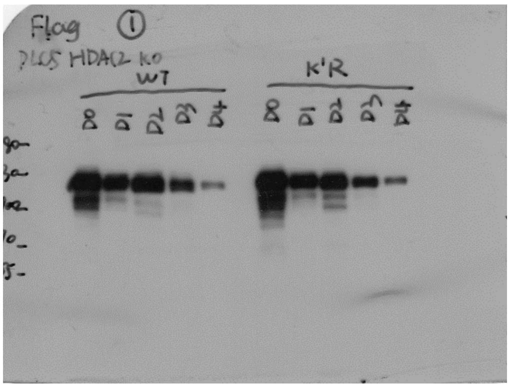

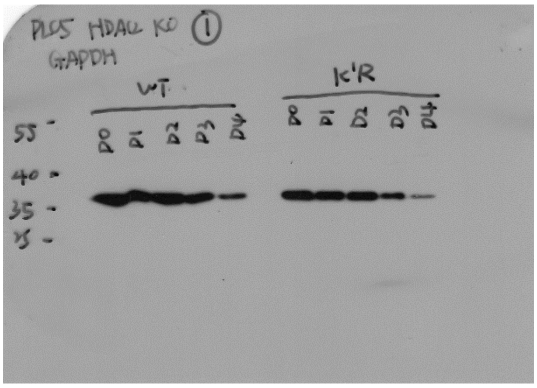

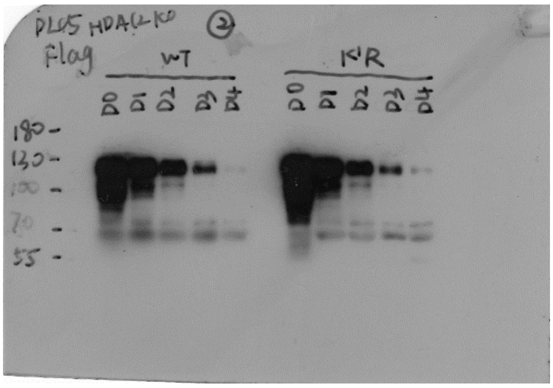

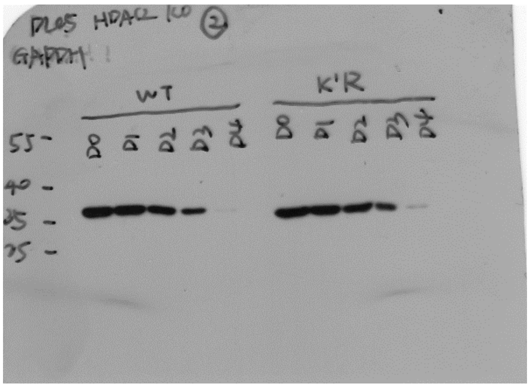

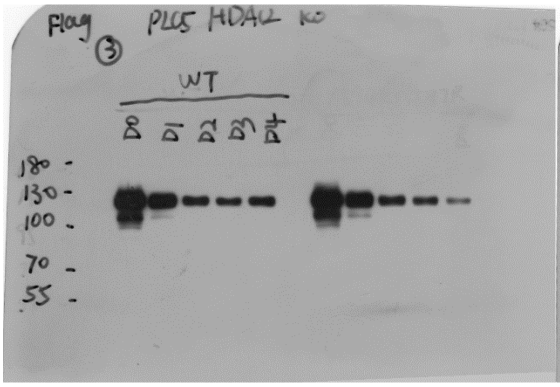

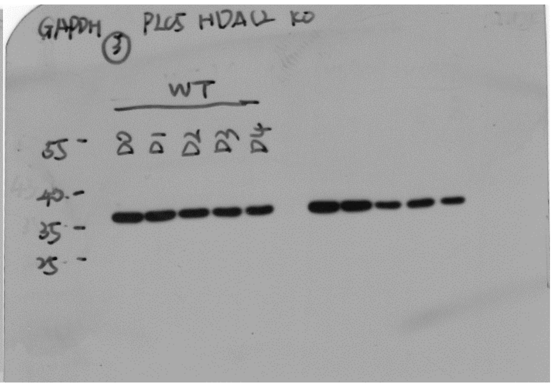

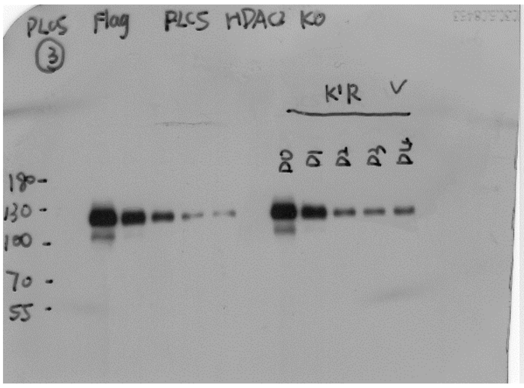

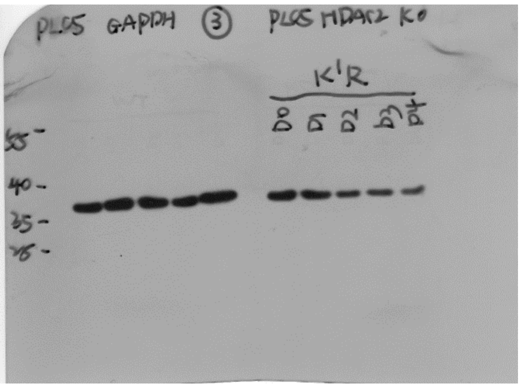

K2R+K3R

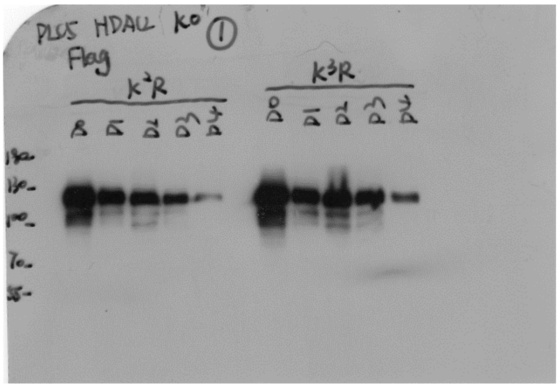

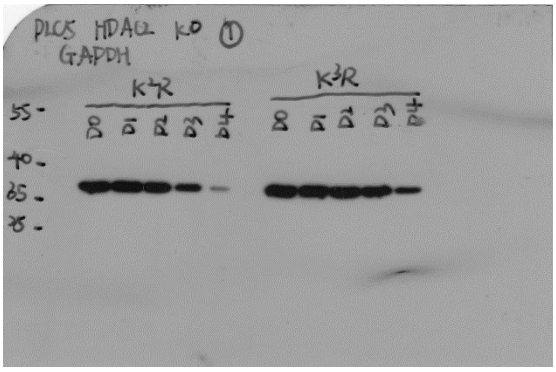

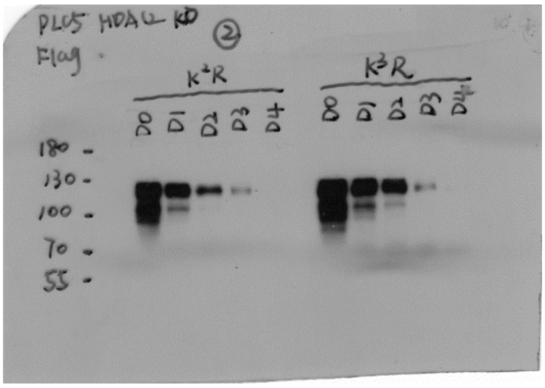

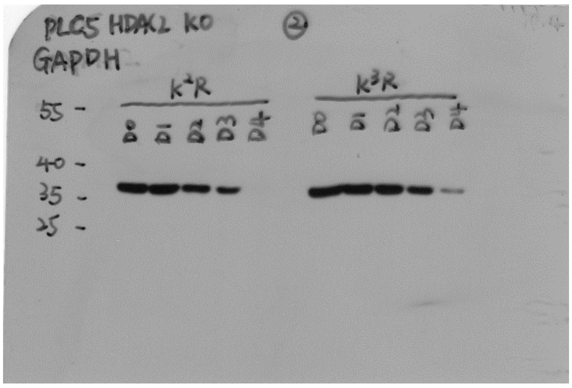

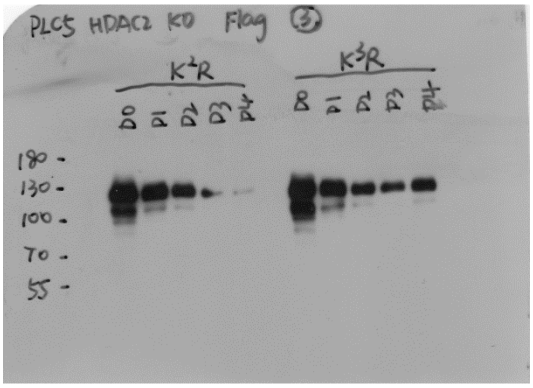

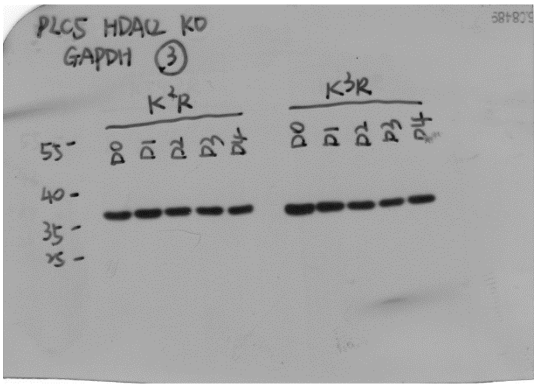

3KR

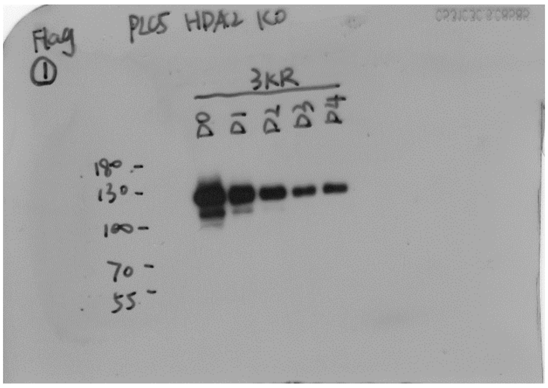

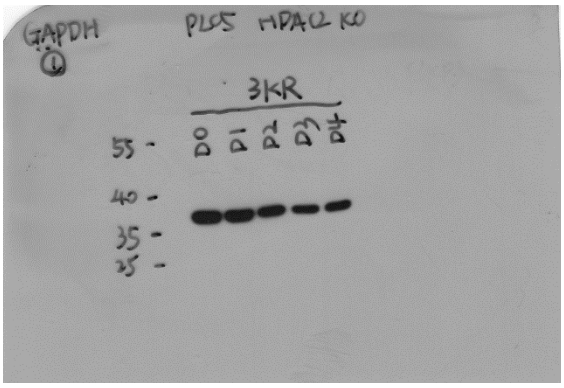

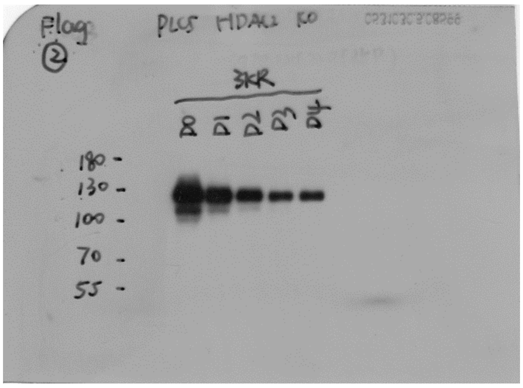
Flag
②
PLC5 HDAC2 KO
3KR
D0 D1 D2 D3 D4
180
130
100
70
55

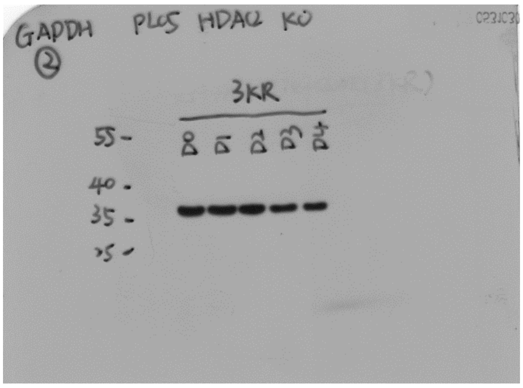
GAPDH
②
PLC5 HDAC2 KO
3KR
D0 D1 D2 D3 D4
55
40
35
25

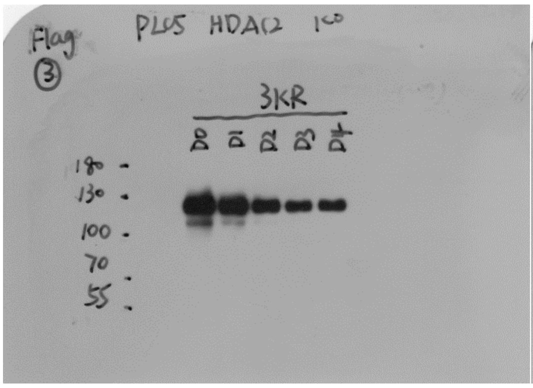
Flag
③
PLC5 HDAC2 KO
3KR
D0 D1 D2 D3 D4
180
130
100
70
55

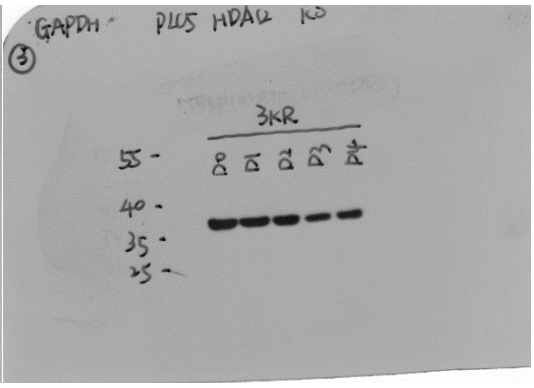
GAPDH
③
PLC5 HDAC2 KO
3KR
D0 D1 D2 D3 D4
55
40
35
25

Figure S6B MHCC97H

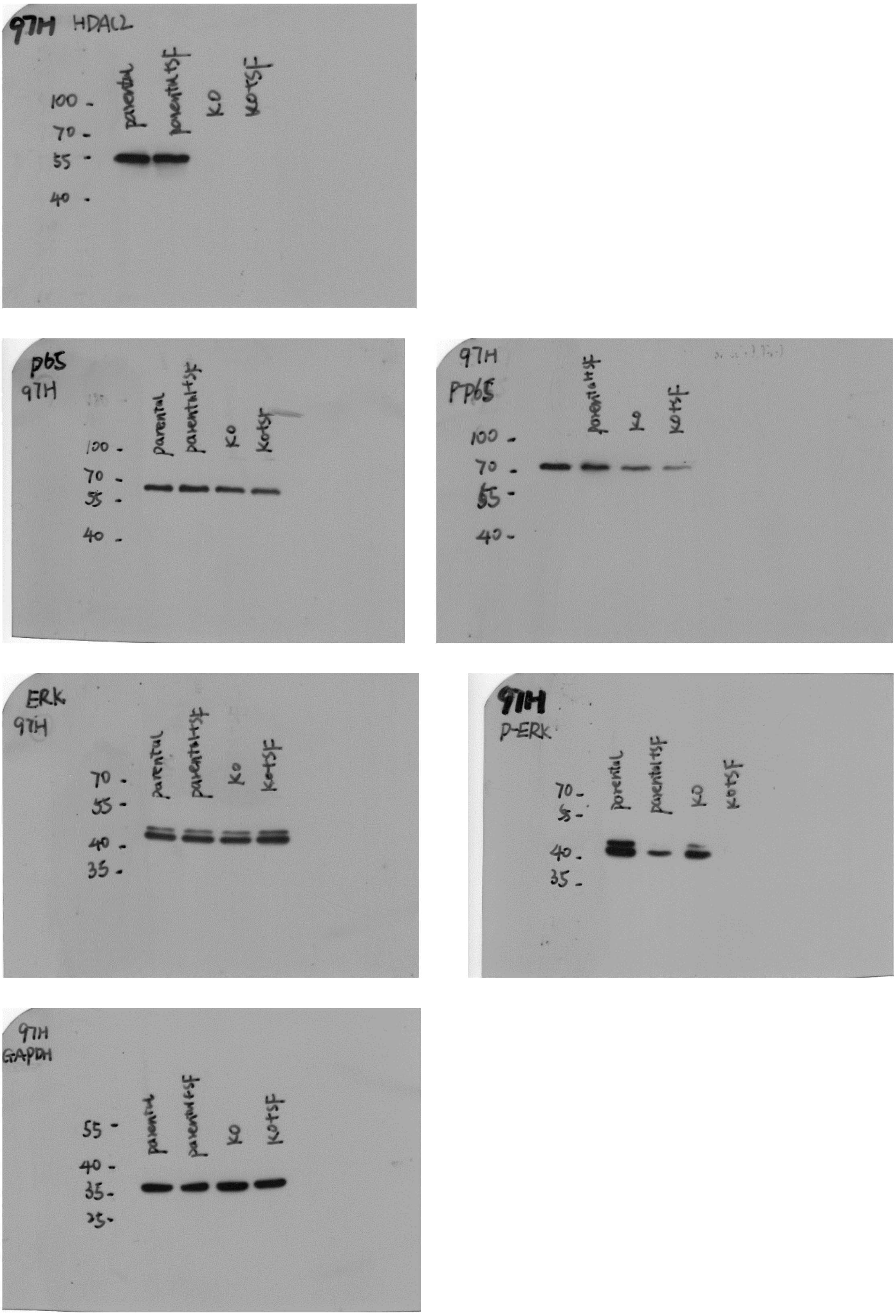

Figure S6B PLC/PRF/5

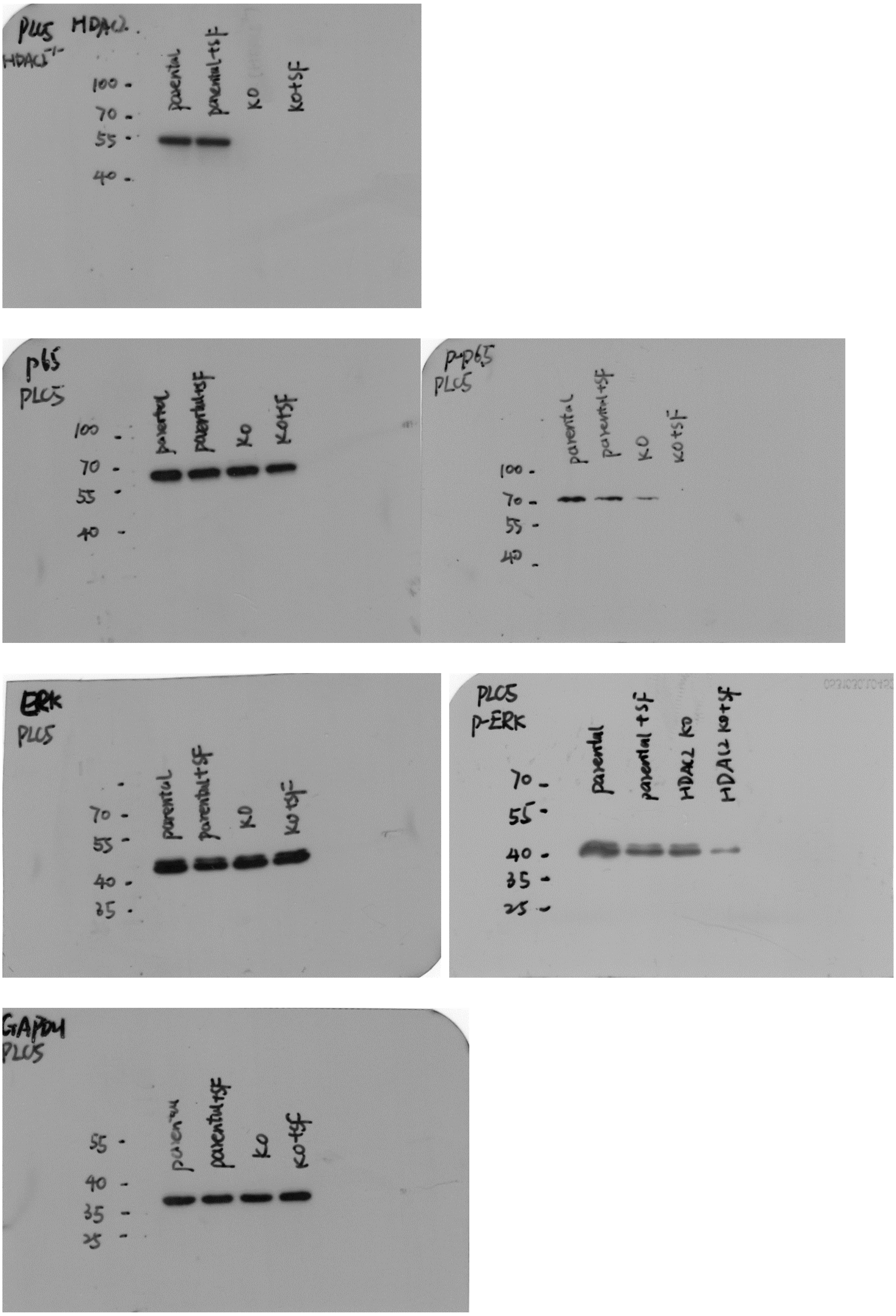

Figure S6C Huh7

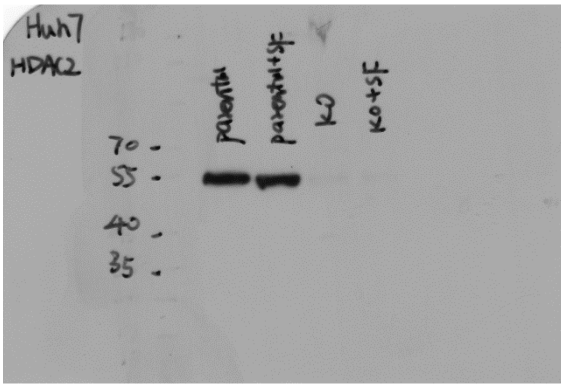

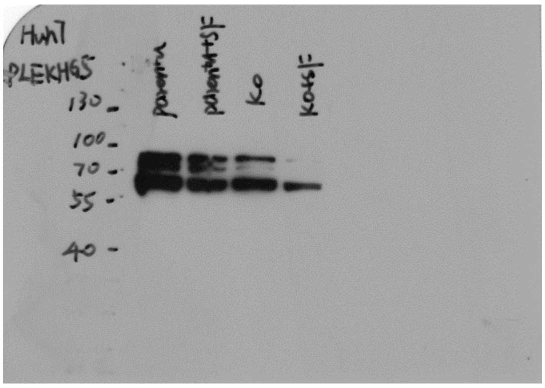

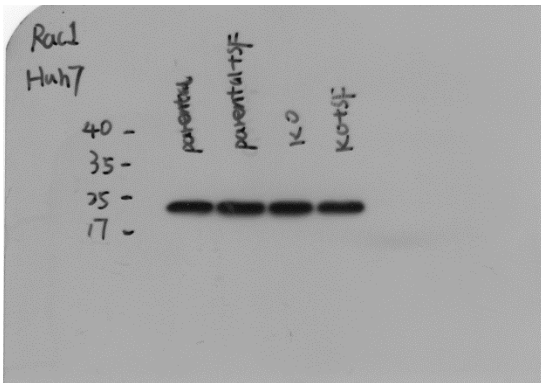

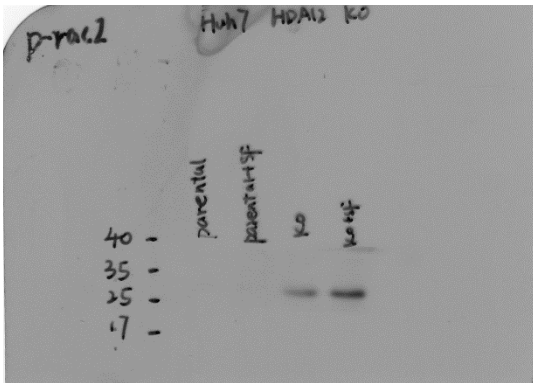

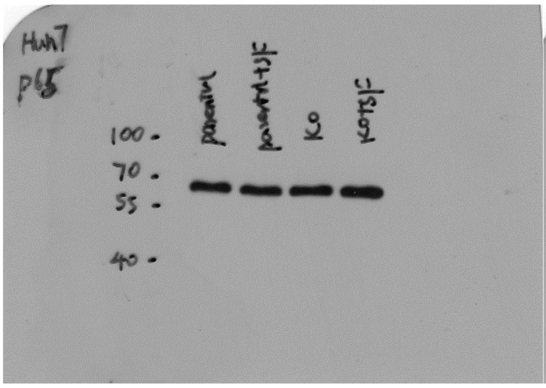

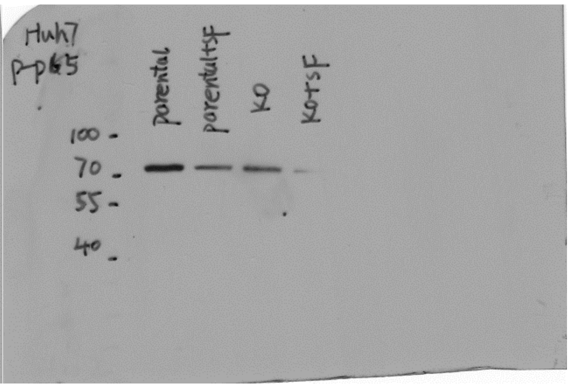

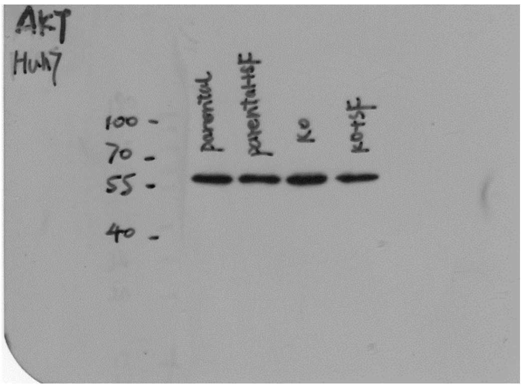

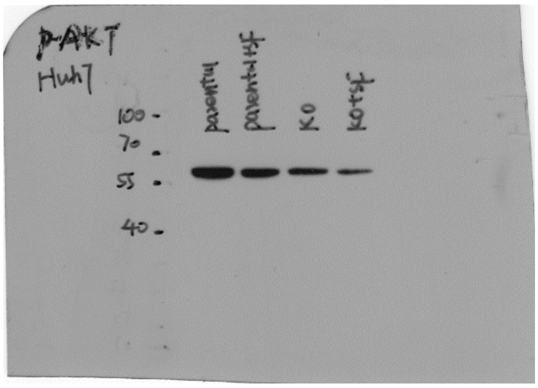

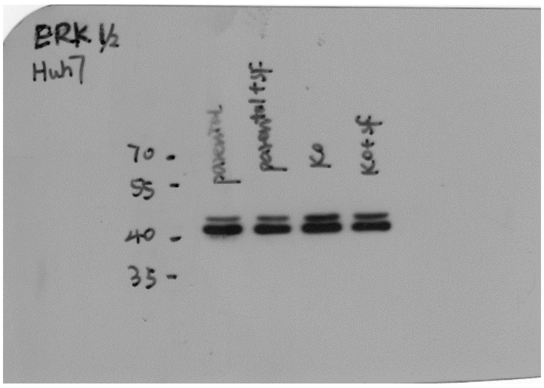
ERK 1/2
Huh7
70
55
40
35
parental
parental+SF
KO
KO+SF

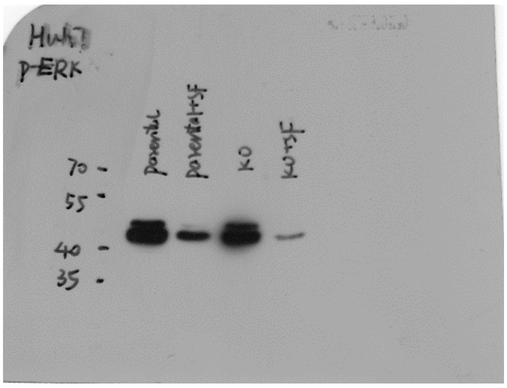
Huh7
p-ERK
70
55
40
35
parental
parental+SF
KO
KO+SF

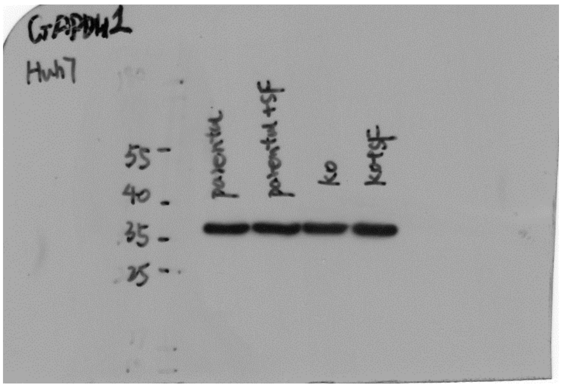
GAPDH1
Huh7
55
40
35
25
parental
parental+SF
KO
KO+SF

Figure S6D

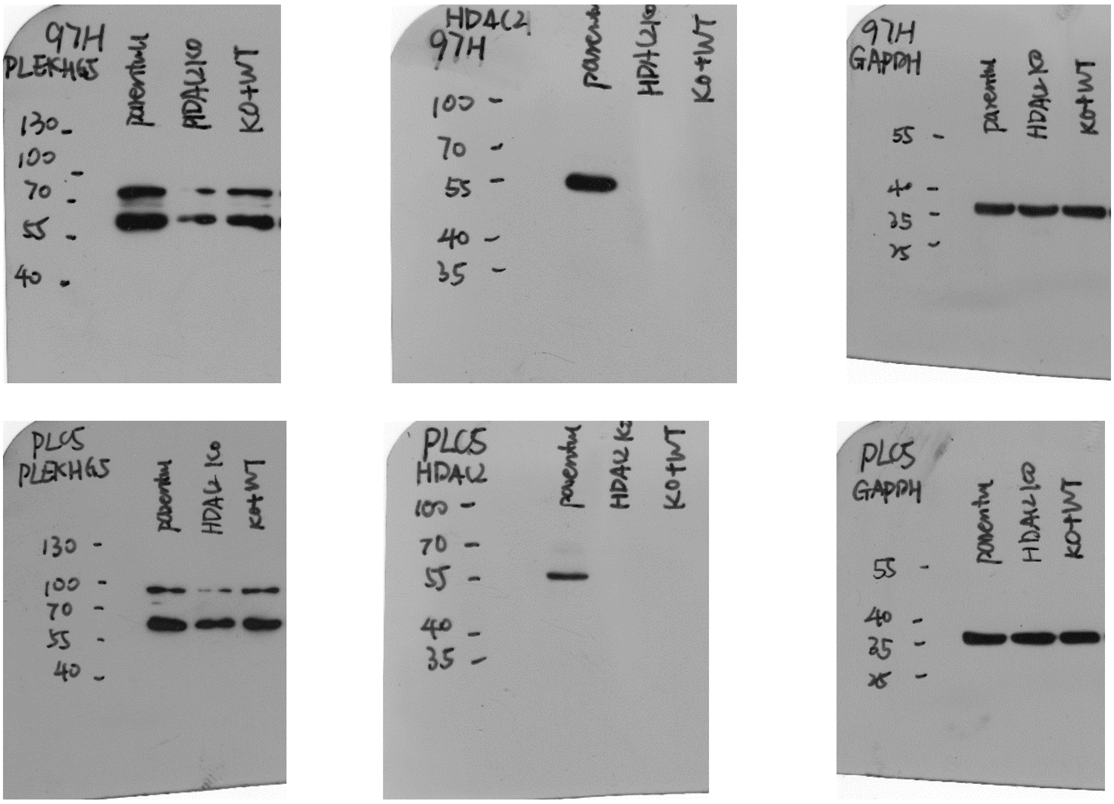
97H
PLEKHG5
HDAC2
GAPDH
PLC5
parental
HDAC2 KO
KO+WT
130
100
70
55
40
35
25

Figure S6G

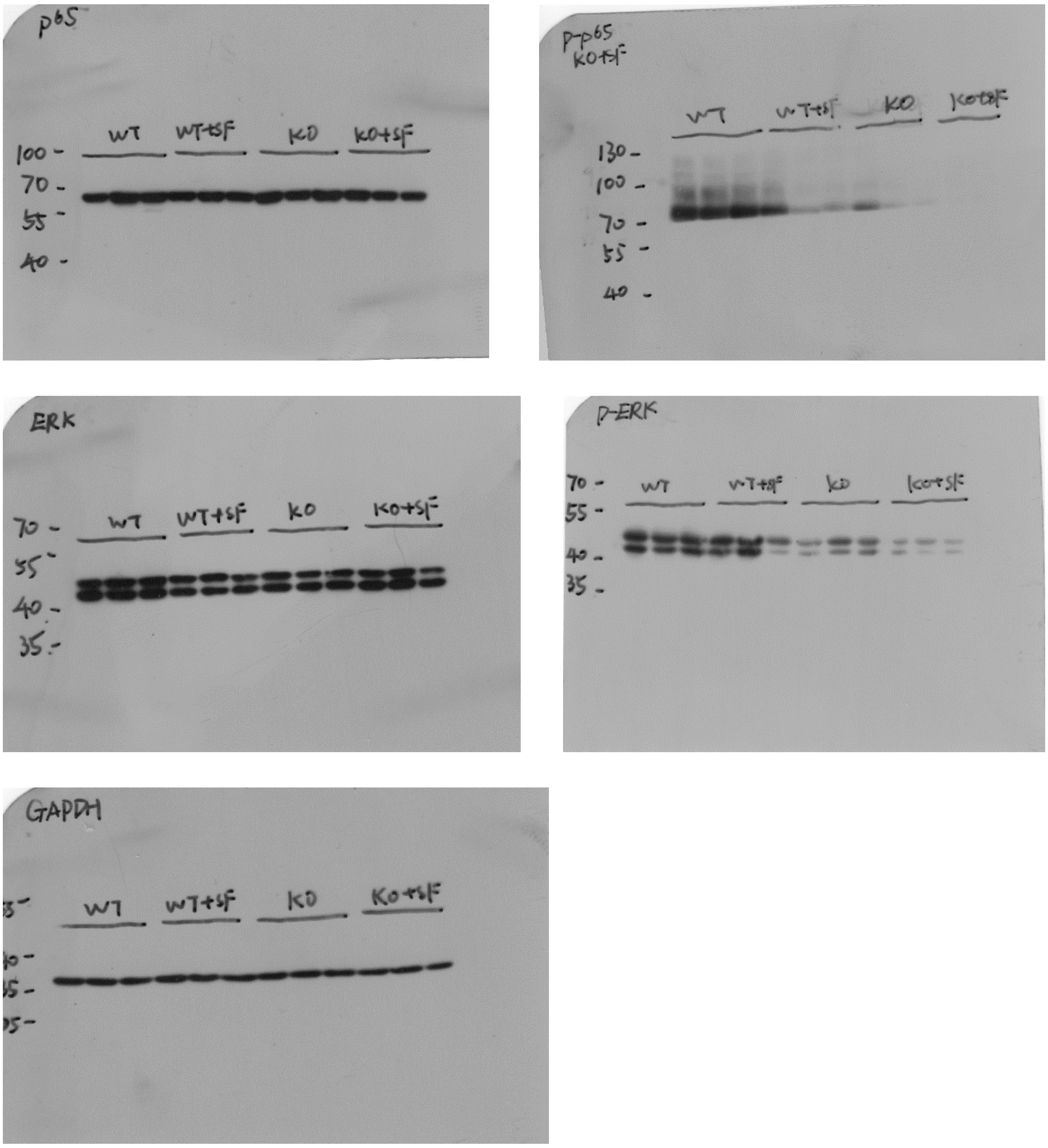

Figure S7D MHCC97H

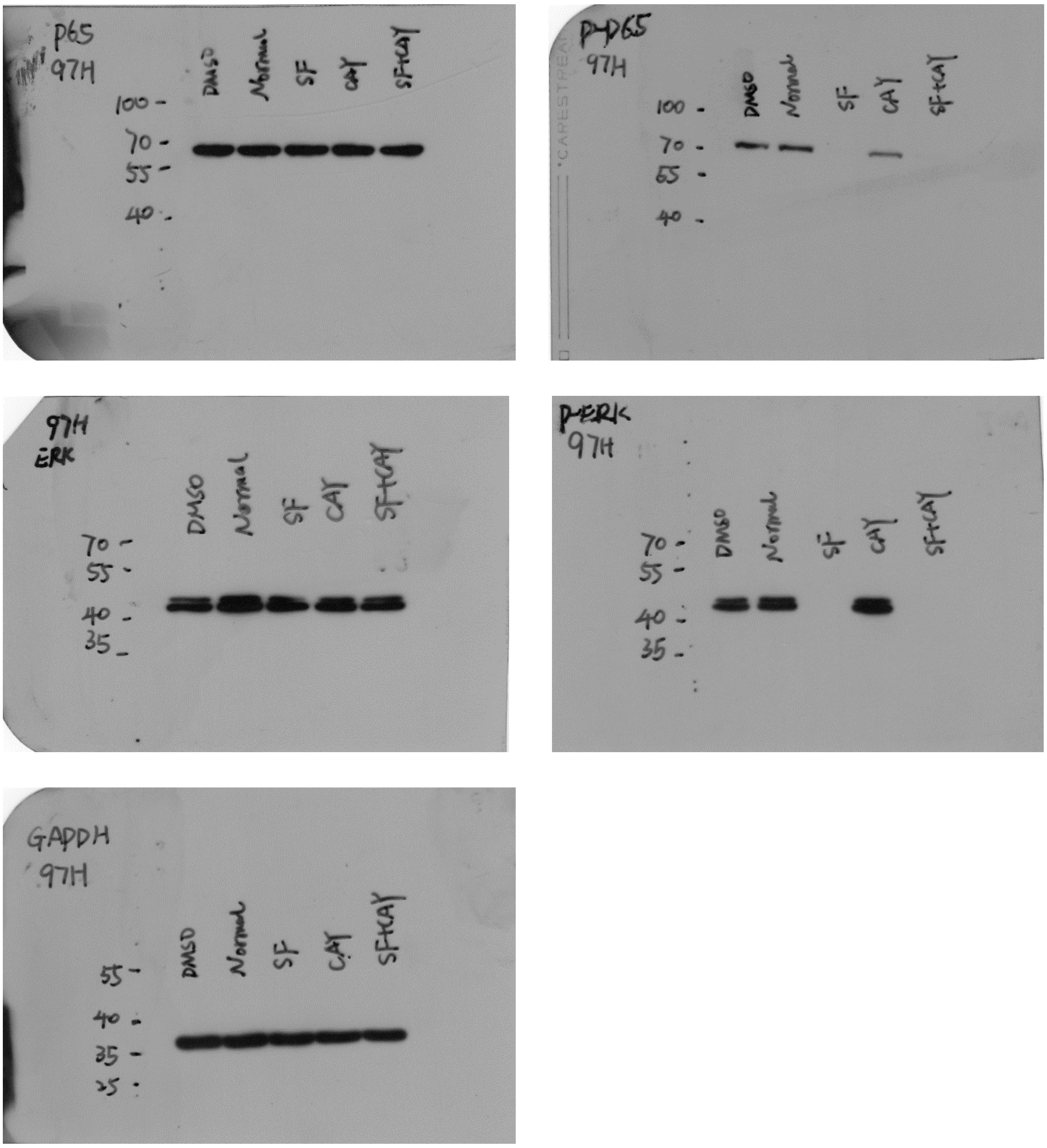

Figure S7D MHCC97H SR

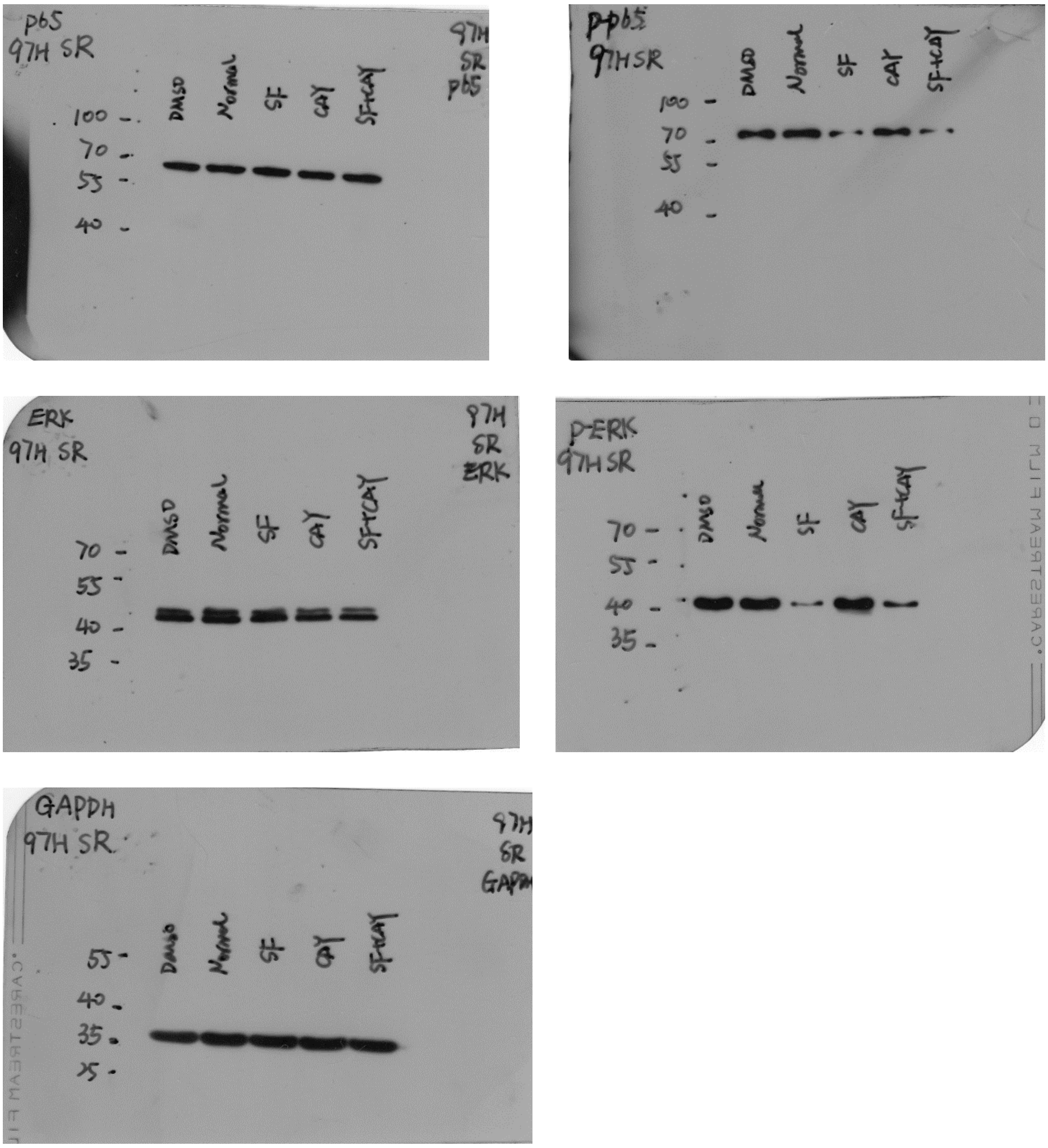

Figure S7D PLC/PRF/5

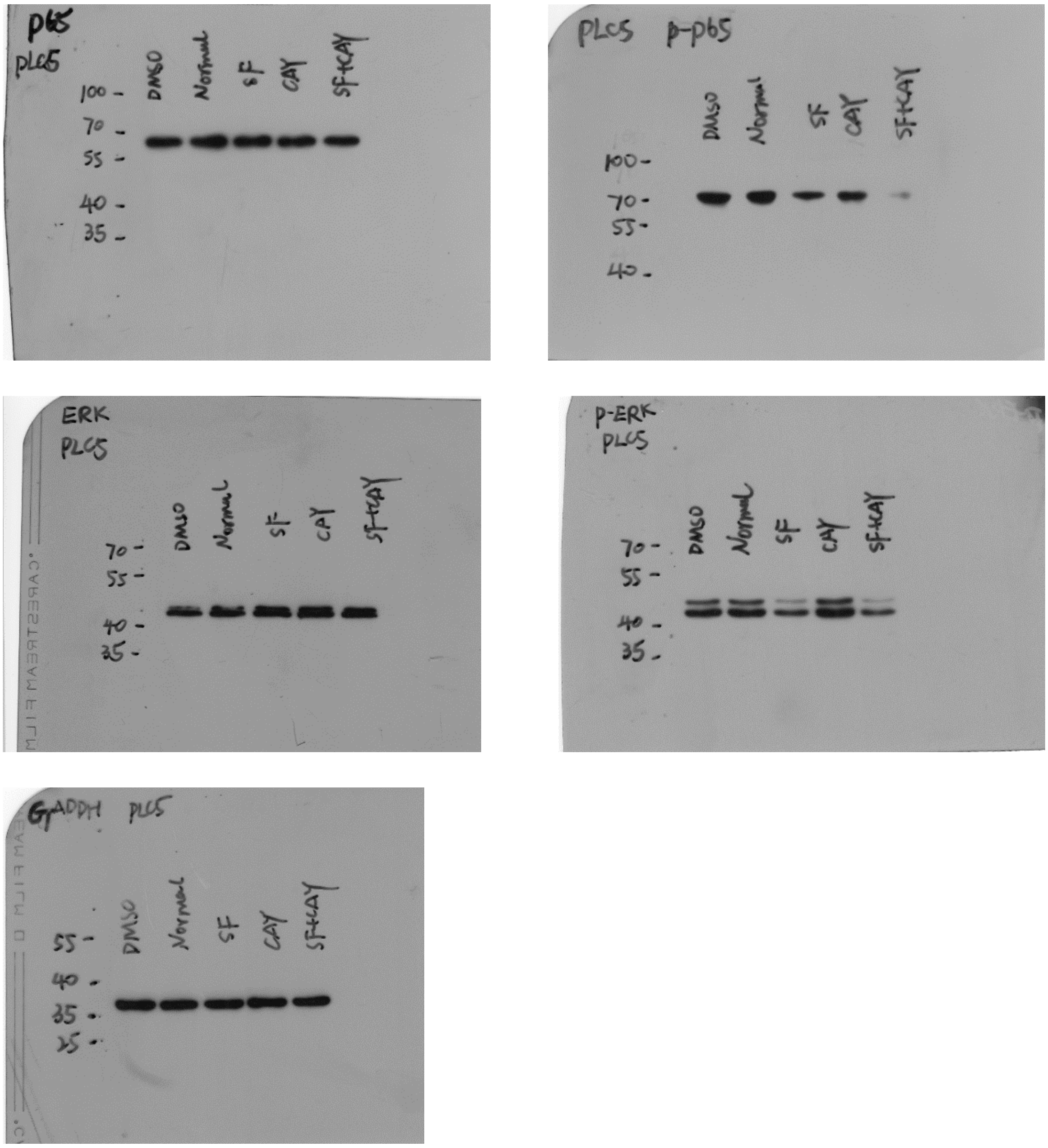

Figure S7D PLC/PRF/5 SR

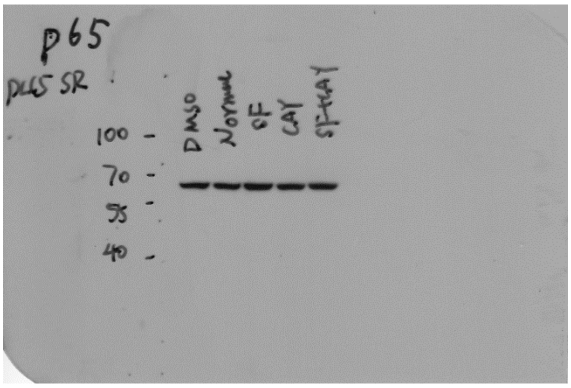

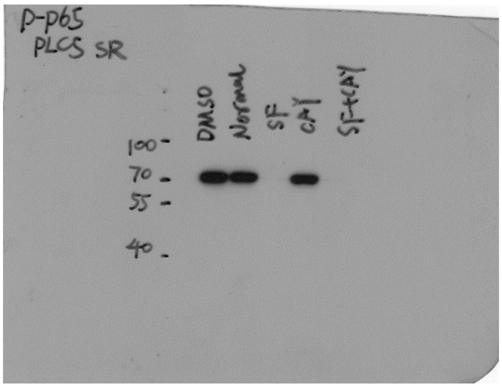

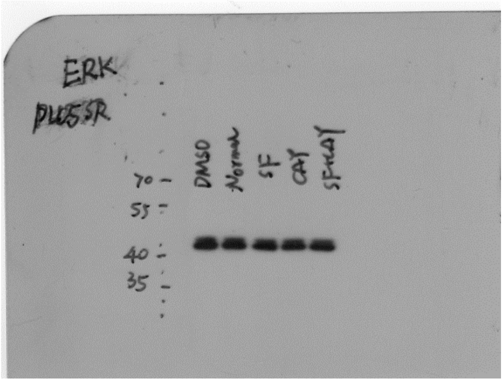

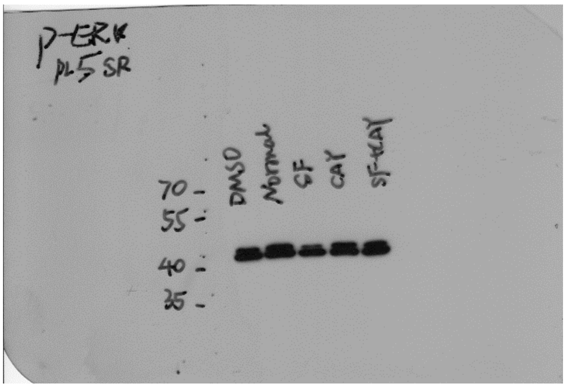

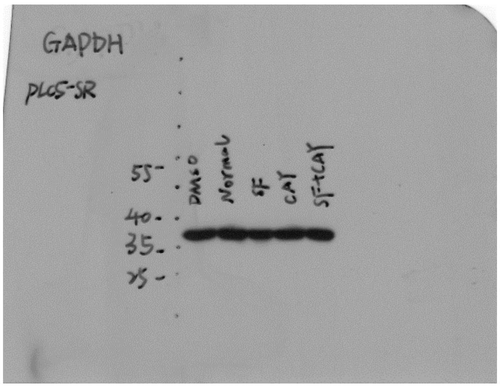

Figure S7D Huh7

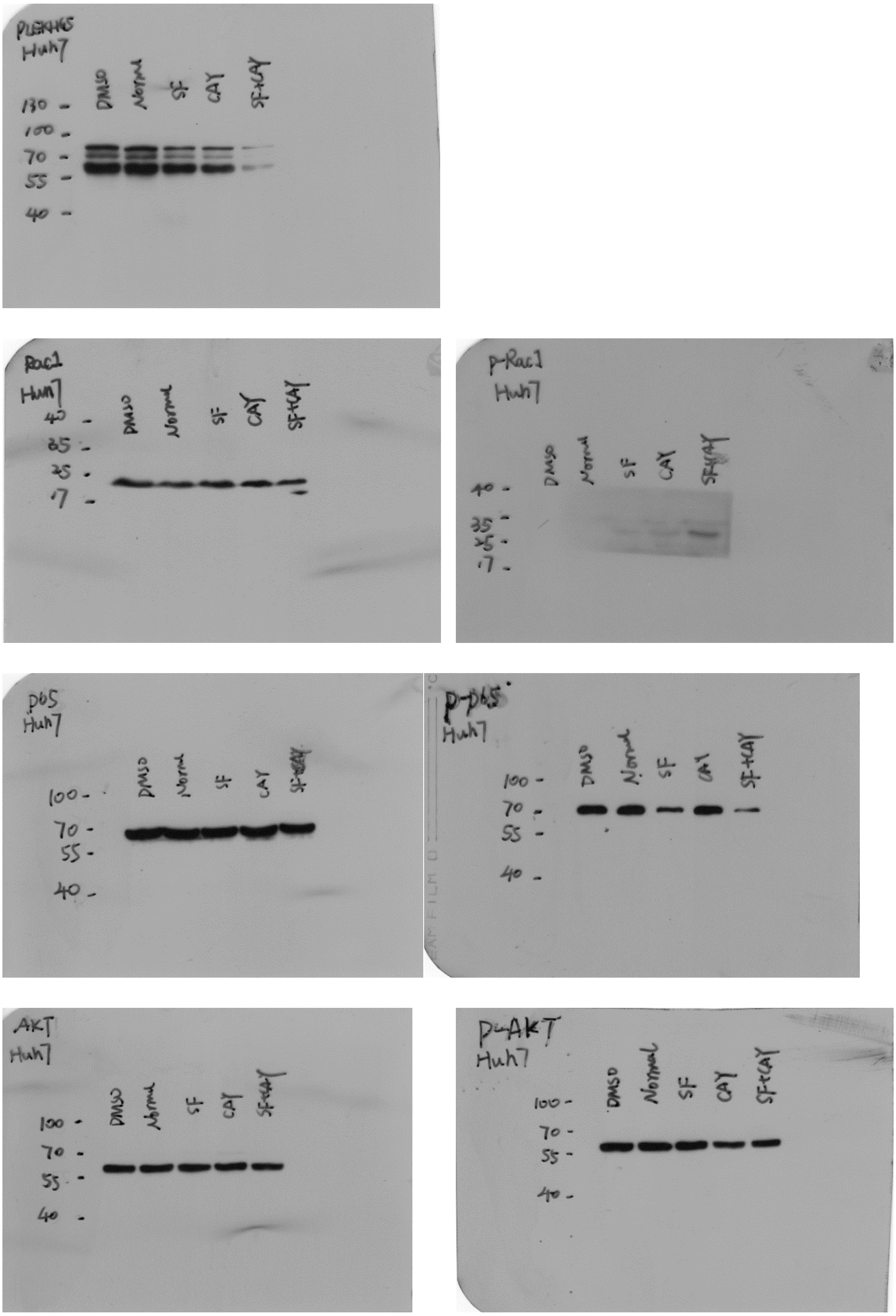

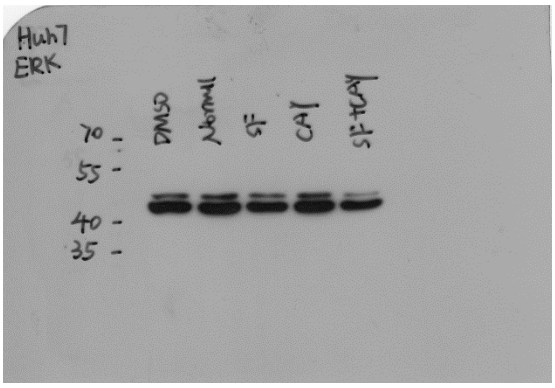
Huh7
ERK
70
55
40
35
DMSO
Normal
SF
CAY
SF+CAY

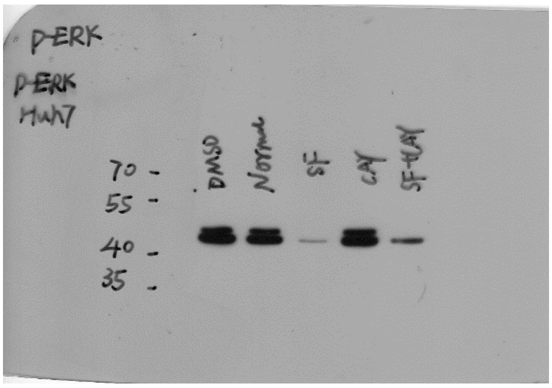
p-ERK
p-ERK
Huh7
70
55
40
35
DMSO
Normal
SF
CAY
SF+CAY

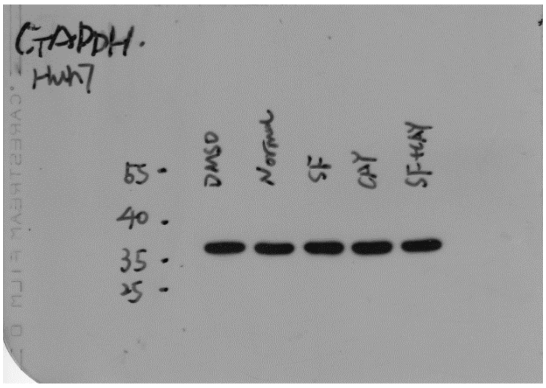
GAPDH.
Huh7
55
40
35
25
DMSO
Normal
SF
CAY
SF+CAY

Figure S7F MHCC97H SR-CDX

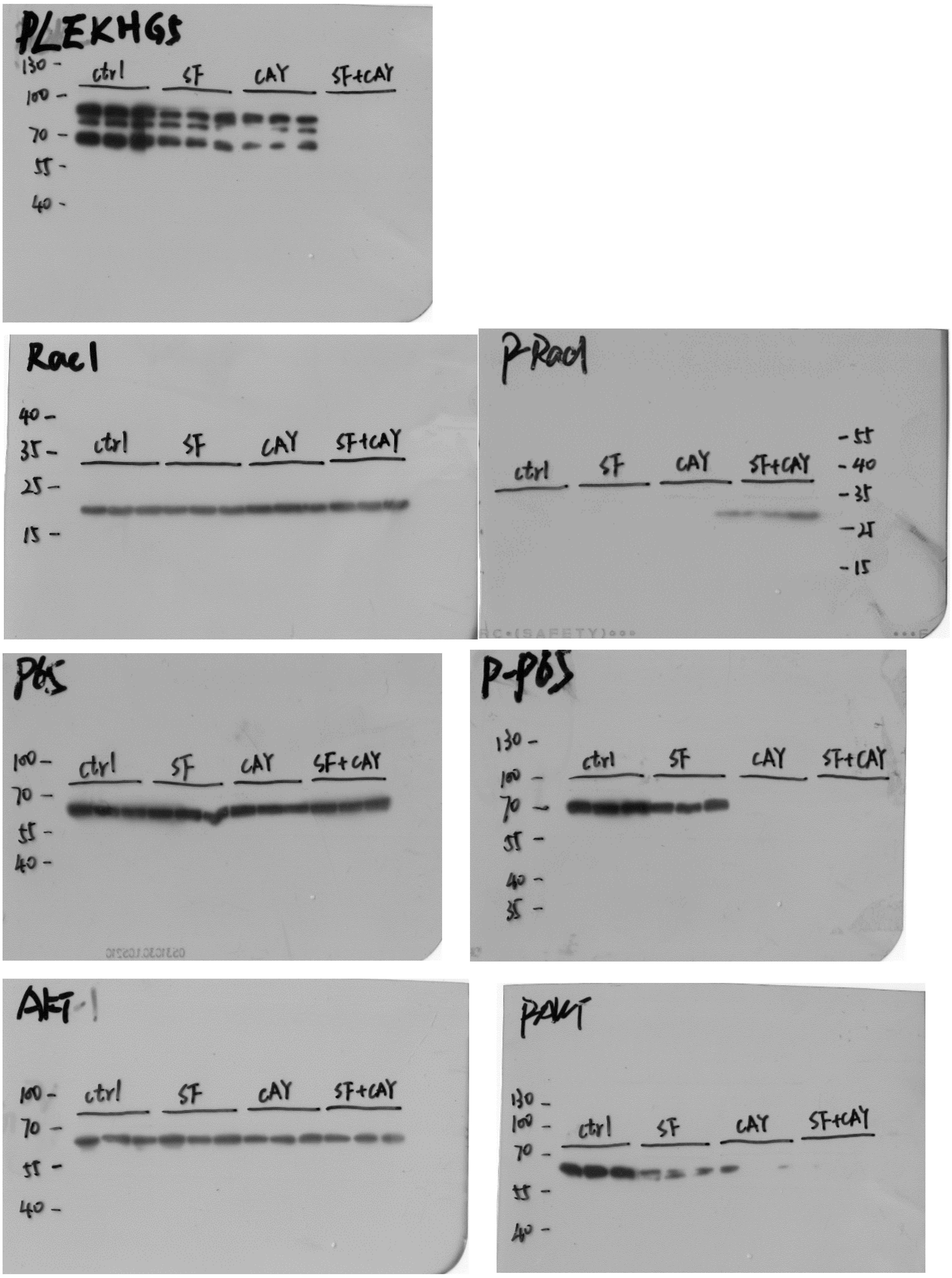

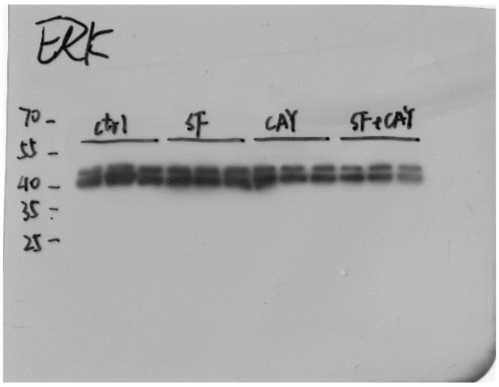
ERK
70
55
40
35
25
ctrl
SF
CAY
SF+CAY

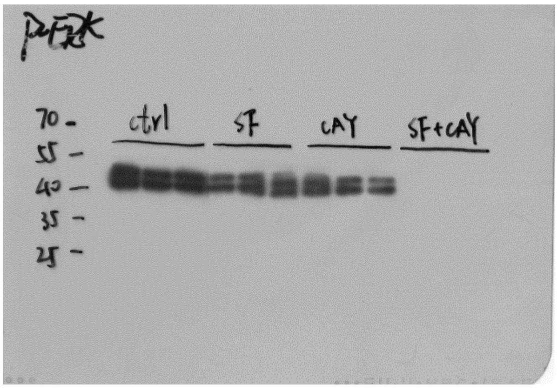
P-ERK
70
55
40
35
25
ctrl
SF
CAY
SF+CAY

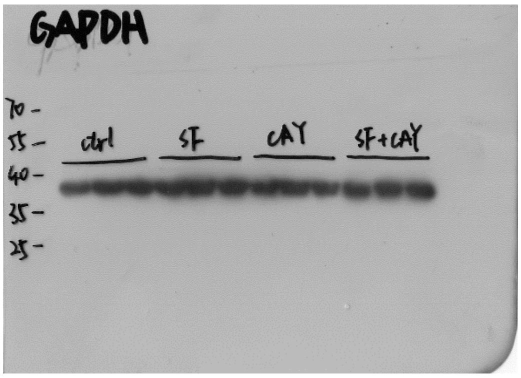
GAPDH
70
55
40
35
25
ctrl
SF
CAY
SF+CAY
